# ﻿Diverse metabolites with anti-psoriasis potential from different fermentations of the fungicolous fungus *Xylarialongipes* HFG1018

**DOI:** 10.3897/imafungus.16.153522

**Published:** 2025-08-12

**Authors:** Zhen-Zhu Zhao, Yan Wang, Xiao-Yu Wang, Hui Chen, Zhen-Zhen Wang, Jing-Kun Wang, Le-Le Wang, Ming-Jun Shen, Xin Pang, Wei-Sheng Feng

**Affiliations:** 1 School of Pharmacy, Henan University of Chinese Medicine, Zhengzhou 450046, China Henan University of Chinese Medicine Zhengzhou China; 2 Academy of Chinese Medical Sciences, Henan University of Chinese Medicine, Zhengzhou 450046, China Henan University of Chinese Medicine Zhengzhou China

**Keywords:** Chemical investigation, different fermentation conditions, HaCaT cells, immunosuppression, psoriasis, *Xylarialongipes*, *Xylariaceae*

## Abstract

The genus *Xylaria* is a promising source of bioactive compounds. This study examined the diversity of secondary metabolites (SMs) in *Xylarialongipes* under three fermentation conditions, resulting in the isolation of 14 new SMs and 27 known ones. The structures, including absolute configurations, were determined using NMR spectroscopy, HRESIMS analysis, and computational methods (ECD, ¹³C NMR data, and optical rotation). A comprehensive library of SMs was established, enabling metabolomics-wide association studies that identified culture conditions as a key factor influencing SM production. This compound library also facilitates the determination of absolute configurations for diplosporins by analyzing *J* values and CD trends. Anti-proliferative tests against induced T/B lymphocytes and HaCaT cells revealed that over half of the compounds exhibited significant inhibitory activity, with compounds **2**, **15**, and **32** reducing IFN-*γ* secretion. Compound **32** demonstrated promising anti-psoriatic effects by inhibiting NF-*κ*B p65 phosphorylation in HaCaT cells. This initial systematic chemical study of *X.longipes* under different conditions provides insights into structure–activity relationships.

## ﻿Introduction

Fungi from the genus *Xylaria* are primarily saprophytic, though some exhibit parasitic behavior. They thrive in forest ecosystems, often near ant colonies or in symbiotic relationships with plants (U’Ren et al. 2016). These unique ecological niches endow *Xylaria* species with distinctive features (Chen et al. 2024). Research has identified terpenoids (Chen et al. 2020a, 2020b, 2024), sterols (Chen et al. 2024), alkaloids (Chen et al. 2024), cyclopeptides (Chen et al. 2024; Li et al. 2011; Wu et al. 2011), and polyketides (Chen et al. 2024; Masahiko et al. 2014; Ibrahim et al. 2014) as the main chemical constituents produced by *Xylaria* species. Pharmacological studies have shown that extracts or compounds from *Xylaria* display a broad range of biological activities, including antioxidant, antibacterial, antitumor, enzyme inhibitory, and immunosuppressive effects, highlighting the genus’s significant research value and potential applications (Chen et al. 2024).

It is widely recognized that many microbial gene clusters remain silent under standard fermentation conditions. These “silent” or “cryptic” biosynthetic gene clusters may encode novel natural products, representing a vast reservoir of potential drug candidates. The “one strain many compounds” (OSMAC) approach, pioneered by Professor Zeeck and colleagues, involves altering medium composition and other parameters to activate these silent genes, thereby inducing the production of novel secondary metabolites (SMs) (Bode et al. 2002; Pan et al. 2019). This strategy has proven highly effective in unlocking the biosynthesis of cryptic natural products, yielding novel compounds with significant potential (Pfütze et al. 2024; Zhang et al. 2015).

**Figure 1.**
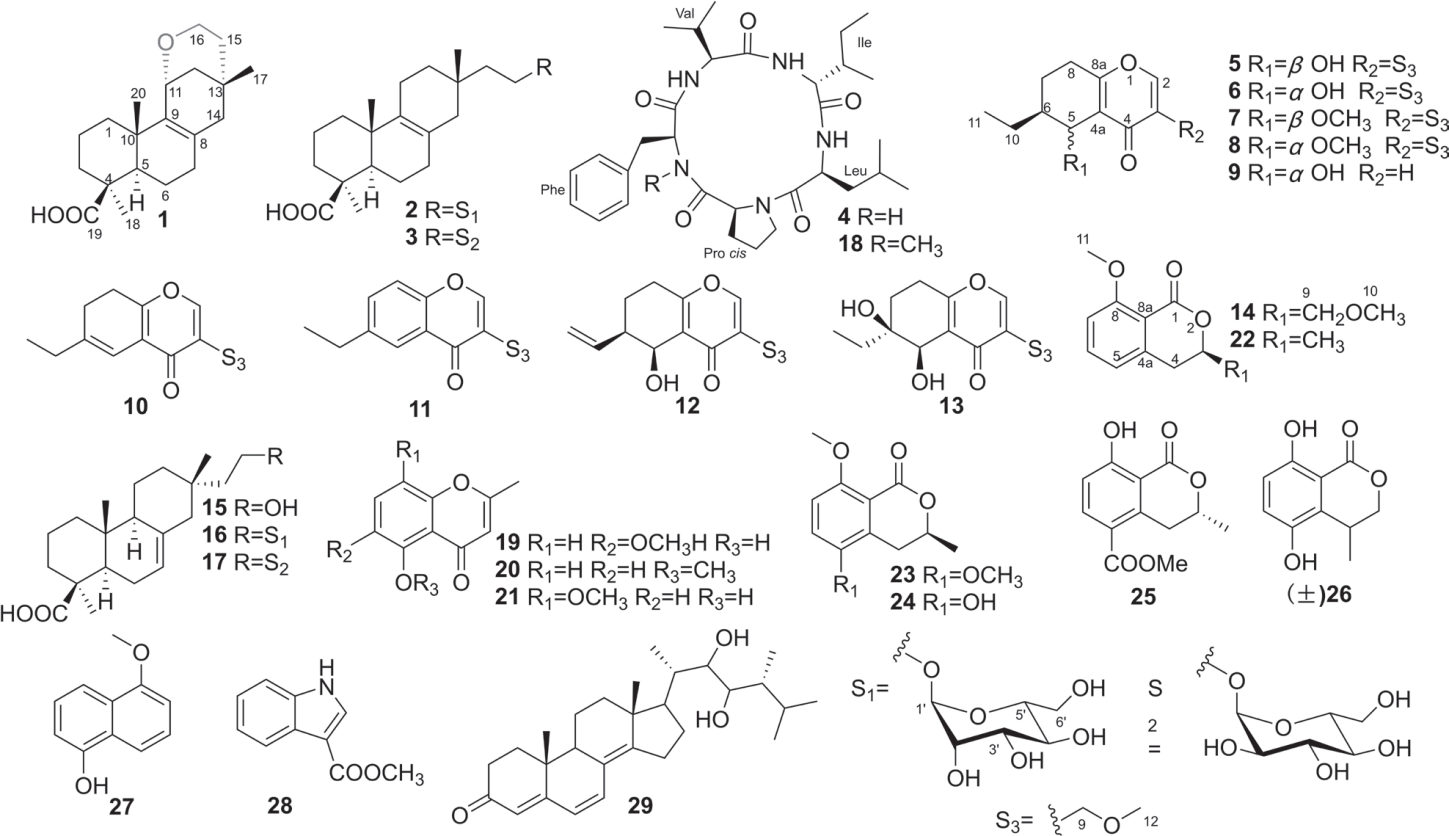
Chemical structures of **1**–**29** from the rice fermentation of *X.longipes* HFG1018.

**Figure 2.**
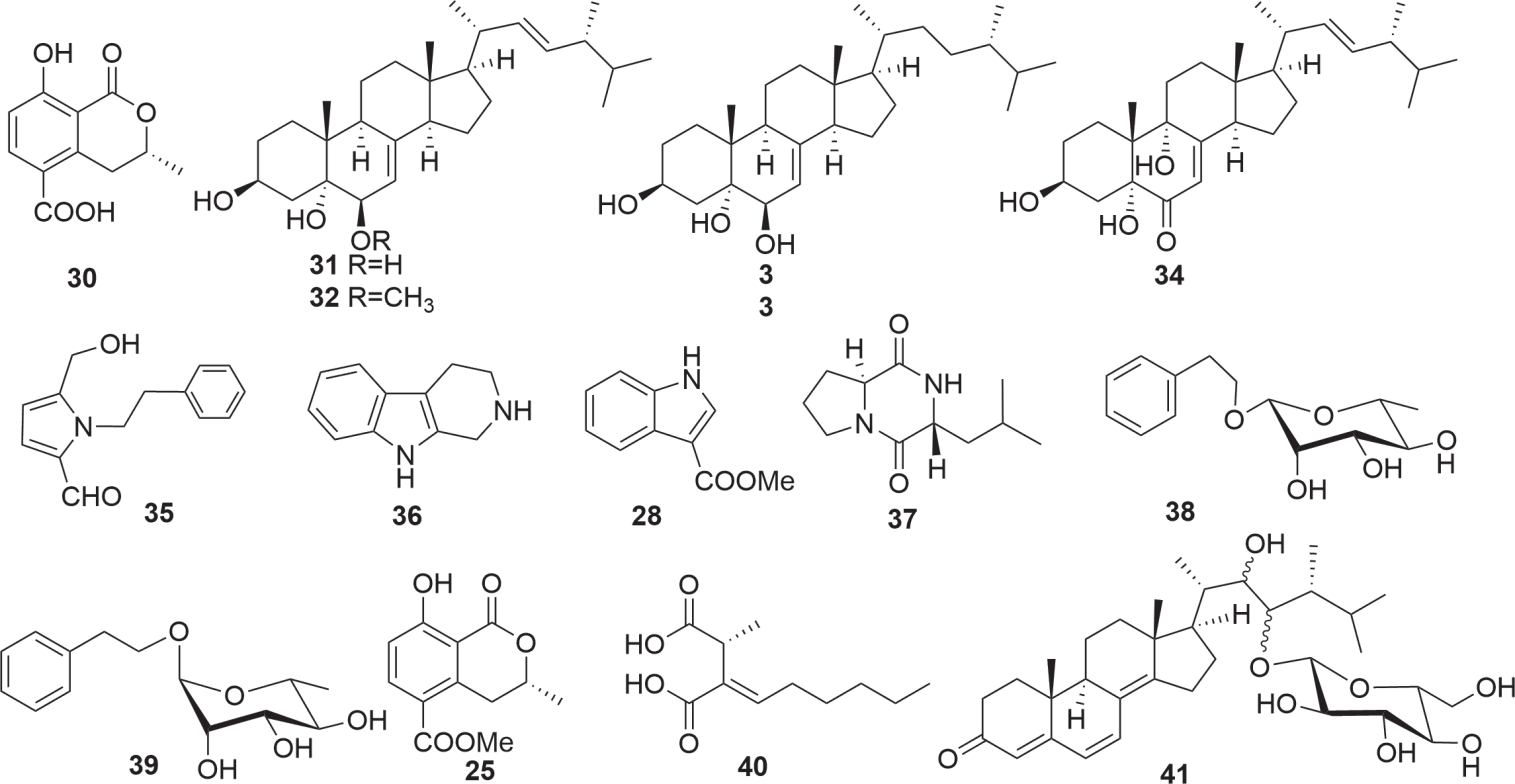
Chemical structures of **30**–**34** (PDB) and **25**, **28**, **35**–**41** (GPY) from liquid fermentations of *X.longipes* HFG1018.

*Xylarialongipes* HFG1018, isolated from the medicinal fungus *Fomitopsisbetulina* (Bull.) K. Cui, M.L. Han & Y.C. Dai (*Fomitopsidaceae*), is a typical ectomycorrhizal fungus within the family *Xylariaceae*. Fungicolous fungi (FF), having co-evolved with their hosts through long-term natural selection, offer valuable insights into host–fungus interactions through the study of their SMs (Sun et al. 2019). Previous research on *X.longipes* HFG1018 led to the isolation of a series of nor-pimarane diterpenes and two novel bicyclo[2.2.2]octane diterpene skeletons with immunosuppressive properties from its liquid fermentation (Chen et al. 2020a, 2020b). To further explore its chemical diversity, three distinct fermentation systems were employed in this study, resulting in the isolation of 14 new compounds—including three pimarane-type diterpenes (**1–3**), one pentapeptide (**4**), nine 6-ethyl-4*H*-chromen-4-one derivatives (diplosporins) (**5–13**), and one isochromone (**14**)—alongside 27 known compounds (Figs 1, 2). Notably, all new SMs were obtained from rice fermentation. Among these, compound **1** represents the first example of a 6/6/6/6-fused tetracyclic pimarane; compound **4** is a rare pentapeptide; and compound **9** is a novel nordiplosporin.

Psoriasis is an almost incurable skin disease characterized by excessive immune response and over-proliferation of keratinocytes. Antiproliferative assays against induced T/B lymphocytes (immune cells) and a keratinocyte cell line (HaCaT) revealed that several compounds exhibit promising anti-psoriasis activity. This study details the isolation, structural elucidation, and bioactivity evaluation of these metabolites, contributing to the expanding knowledge of *Xylaria* species and their potential applications.

## ﻿Methods and materials

### ﻿General experimental procedures

Optical UV spectra were recorded using a Shimadzu UV-2401PC UV–visible spectrophotometer (Shimadzu, Kyoto, Japan). IR spectra (in MeOH) were measured on a Bruker Tensor 27 spectrometer (Bruker, Karlsruhe, Germany). Optical rotations were measured using the APIV polarimeter (Rudolph Research Analytical, Hackettstown, NJ, USA). All NMR spectra were acquired on a Bruker Avance III 500 MHz spectrometer (Bruker Corporation, Karlsruhe, Germany). High-resolution electrospray ionization mass spectra (HRESIMS) were recorded on an AB Sciex TripleTOF 6600 system (AB SCIEX, Framingham, MA, USA). Macroporous adsorption resin (Cangzhou Bon Adsorber Technology Co., Ltd., Cangzhou, China), Sephadex LH-20 (Amersham Biosciences, Uppsala, Sweden), and silica gel (Qingdao Haiyang Chemical Co., Ltd., Qingdao, China) were used for column chromatography (CC). Medium-pressure liquid chromatography (MPLC) was performed using an FL-H050G MPLC system (Agela Technologies, Tianjin, China) with either an Agela ODS flash column (C18, 120 g, 40–60 mm, Tianjin, China) or MCI gel (CHP20/P120, Mitsubishi Chemical Corporation, Yokohama, Japan). Preparative high-performance liquid chromatography (prep. HPLC) was carried out using a SEP LC-52 system equipped with a YMC-pack ODS-A column (250 × 10 mm i.d., 5 μm, 4 mL/min; or 250 × 20 mm i.d., 5 μm, 7 mL/min; YMC, Tokyo, Japan) and an MWD UV detector (Separation Technology Co., Ltd., Beijing, China). All solvents were of analytical or chromatographic grade (TJshield Fine Chemicals Co., Ltd., Tianjin, China). All elution systems are described in terms of volume ratio. Concanavalin A (ConA), lipopolysaccharide (LPS, *Escherichiacoli* 055:B5), CCK-8, and RPMI 1640/DMEM media were purchased from Solarbio Life Sciences (Beijing, China). Fetal bovine serum (FBS) was obtained from HyClone Laboratories (Utah, USA).

### ﻿Fungal material and taxonomy

#### ﻿Sample collection and morphological characterization

The fungus *Xylarialongipes* HFG1018 (*Xylariaceae*) was isolated from the wood-rotting fungus *Fomitopsisbetulina* (Bull.) K. Cui, M.L. Han & Y.C. Dai (*Fomitopsidaceae*). The fruiting bodies of *F.betulina* (Bull.) K. Cui, M.L. Han & Y.C. Dai were harvested from Changbai Mountain National Nature Reserve, Jilin Province, China, in 2012 and identified by the *ITS* sequence (GenBank accession No. PQ844712). *Xylarialongipes* was identified by combined analysis of the *β*-tubulin gene sequence (*TUB2*, GenBank accession No. MN583256), the internal transcribed spacer (*ITS*, GenBank accession No. PV523290), and the RNA polymerase II second-largest subunit gene (*rpb2*, GenBank accession No. PV558833). The *TUB2* gene was previously obtained and deposited in GenBank (Chen et al. 2020a, 2020b), while the *ITS* sequence and *rpb2* gene were obtained and deposited in GenBank for the first time in this study. The strain *X.longipes* (No. HFG1018) is preserved at the Mushroom Bioactive Natural Products Research Group, South-Central University for Nationalities (Principal Investigator: Professor Liu Ji-Kai). This strain was later gifted by Professor Liu and is also currently maintained at the Department of Traditional Chinese Medicine and Natural Medicinal Chemistry, Henan University of Chinese Medicine (preservation number: HFG1018).

Pure cultures were maintained at 25 °C for 1–4 weeks on oatmeal agar (OA) medium. Mycelium stained with methylene blue was examined for microscopic features using a biological microscope. Micro-characteristics were observed under an ML11-II biomicroscope (Guangzhou Mingmei Optoelectronic Technology Co., Ltd., China) and photographed using an iPhone 13 Pro camera.

#### ﻿Sequence alignments and phylogenetic analyses

All obtained sequences were deposited in GenBank (Table 1). The sequences were analyzed using the BLAST tool in the NCBI GenBank database to identify highly similar sequences. Single sequences (*ITS*, *TUB2*, and *rpb2*) were aligned using the Clustal W algorithm in MEGA v.12 and manually adjusted for maximum sequence similarity. Multiple sequences were concatenated using PhyloSuite software (Zhang et al. 2020). Phylogenetic trees were constructed using the concatenated sequences (*ITS*, *TUB2*, and *rpb2*) in MEGA v.12 with the neighbor-joining (NJ) and maximum-likelihood (ML) methods. The multi-locus phylogenetic tree included the fungal isolate studied here and closely related species of the genus *Xylaria*, with *Engleromycessinensis* and *Collodisculabambusae* used as outgroup taxa. Clade stability was evaluated with 1,000 bootstrap replicates.

**Table 1.** GenBank accession numbers in this study.

Species	Culture collection	Country	*ITS*	*rpb2*	*TUB2*
*Xylarialongipes*	CBS 148.73	–	MH860649.1	KU684280.1	KU684204.1
*Xylariaallantoidea*	HAST 94042903	China	GU324743	GQ848356	GQ502692
*Xylariaregalis*	HAST 920	India	GU324745	GQ848358	GQ502694
*Xylariamontagnei*	HAST, JF 495	Martinique	GU322455	GQ848337	GQ495948
*Xylariaberteri*	JDR 256	United States	GU324750	GQ848363	GQ502698
*Xylariaberteri*	90112623	China	GU324749	GQ848362	AY951763
*Xylariazonghuangii*	GMB1131	China	OR469030	OR753878	OR485623
*Xylariazonghuangii*	GMB1132	China	OR469027	OR753879	OR485624
*Xylariatongrenensis*	GMB1114	China	OR469015	OR887303	OR485609
*Xylariatongrenensis*	GMB1169	China	OR469016	OR887304	OR485610
*Xylariapseudocubensis*	GMB1089	China	OR468997	OR887282	OR485588
*Xylariapseudocubensis*	GMB0775	China	OR468999	OR887283	OR485589
*Xylariacubensis*	JDR 860	United States	GU991523	GQ848365	GQ502700
Xylariacf.castorea	HAST 91092303	China	GU324752	GQ853019	GQ502704
*Xylariacastorea*	PDD600	New Zealand	GU324751	GQ853018	GQ502703
*Xylariashuqunii*	GMB1105	China	OR469012	OR887299	OR485603
*Xylariashuqunii*	GMB1106	China	OR469011	OR887300	OR485604
*Engleromycessinensis*	CNUCC 200801	China	MZ622699	MZ622186	MZ622188
*Xylariafeejeensis*	HAST, JF 565	Martinique	GU322452	GQ848334	GQ495945
*Xylariafeejeensis*	JDR 180	Thailand	GU322453	GQ848335	GQ495946
*Xylariafeejeensis*	HAST 92092013	China	GU322454	GQ848336	GQ495947
*Xylariabawanglingenshs*	GMB1023	China	OR468975	OR753861	OR477223
*Xylariabawanglingenshs*	GMB1162	China	OR468976	OR753862	OR477224
*Xylariafrustulosa*	HAST 92092010	China	GU322451	GQ844838	GQ495944
*Xylariafrustulosa*	HAsT,JF 771	Guadeloupe	GU322450	GQ844837	GQ495943
*Xylariabadia*	HAST 95070101	China	GU322446	GQ844833	GQ495939
*Collodisculabambusae*	GZ 62	China	KP054279	KP276675	KP276674

### ﻿Fermentation, extraction, and isolation

#### ﻿Rice fermentation

Rice fermentation was conducted following established protocols. The glucose peptone yeast (GPY) medium used for fermenting *Xylarialongipes* contained glucose (5%), peptone from porcine meat (0.15%), yeast powder (0.5%), KH_2_PO_4_ (0.05%), and MgSO_4_ (0.05%). Two bottles of GPY liquid fermentation medium (350 mL/bottle), inoculated with *X.longipes*, were incubated in a shaker at 24 °C and 150 rpm for 7 days in the dark. Separately, rice and distilled water (H_2_O) were mixed at a mass ratio of 1:1.3 (total rice weight: 20 kg) and distributed into 500 mL flasks, which were then autoclaved. The cultured liquid mycelium was transferred into the sterilized flasks and incubated in the dark for 40 days.

After fermentation, the rice substrate was transferred to an extraction tank and subjected to ultrasound-assisted extraction with 95% ethanol for 3 hours per cycle. This process was repeated three times. The ethanol solution containing metabolites was concentrated under reduced pressure until only water remained. The aqueous concentrate was then extracted four times with an equal volume of ethyl acetate (EtOAc). The EtOAc layer was concentrated under reduced pressure to yield 233.9 g of crude extract. This crude extract was further fractionated using macroporous adsorption resin column chromatography (CC) with a gradient of EtOH–H_2_O (20:80, 40:60, 60:40, 80:20, 100:0), resulting in nine fractions (A–I).

Fractions B–D (68.3, 14.8, and 4.2 g, respectively) and fraction F (27.9 g) were further separated using MPLC. Fractions B–D were processed on an MCI gel column, while fraction F was separated on a C18-ODS column, both employing a gradient elution of methanol (MeOH) in H_2_O (20:80, 40:60, 60:40, 80:20, 100:0). This yielded four to six subfractions for each fraction (B1–B4, C1–C5, D1–D7, and F1–F6, respectively).

Subfraction B2 was subjected to Sephadex LH-20 column chromatography using MeOH as the eluent, resulting in six parts (B2a–B2f). Compounds **5** (286.8 mg, *t*_R_ = 20.7 min) and **6** (57.0 mg, *t*_R_ = 20.0 min) were purified from fraction B2d using preparative HPLC with a gradient of acetonitrile (MeCN)/H_2_O (15:85→100:0, 50 min, 4 mL/min).

Subfraction C1 was fractionated into three parts (C1a–C1c) via Sephadex LH-20 (MeOH). Fraction C1a was further separated using Sephadex LH-20 (acetone), yielding four subfractions (C1a1–C1a4). Fraction C1a1 was separated by prep. HPLC (MeCN/H_2_O 35:65→80:20→100:0, 0→50→60 min, 4 mL/min) to give **23** (16.5 mg, *t*_R_ = 14.7 min) and **25** (347.9 mg, *t*_R_ = 13.0 min). Fraction C1a2 was eluted on a silica gel CC with an isocratic elution of petroleum ether–acetone (1:1) to afford four components (C1a2a–C1a2d). Compounds **7** (61.1 mg, *t*_R_ = 17.3 min) and **11** (5.1 mg, *t*_R_ = 28.2 min) were purified from fraction C1a2a using prep. HPLC (MeCN/H_2_O 20:80→70:30, 60 min, 4 mL/min). Compounds **22** (2.9 mg, *t*_R_ = 30.8 min) and **14** (1.5 mg, *t*_R_ = 31.9 min) were isolated from fraction C1a2c via prep. HPLC with an isocratic elution of MeCN/H_2_O (30:70, 4 mL/min). Fraction C1a2d was separated via prep. HPLC (MeOH/H_2_O 50:50→100:0, 30 min, 8 mL/min), yielding three components (C1a2d1–C1a2d3). Compounds **26** (7.4 mg, *t*_R_ = 16.5 min), **9** (6.7 mg, *t*_R_ = 36.9 min), and **12** (17.4 mg, *t*_R_ = 41.1 min) were obtained from fraction C1a2d1.

Fraction C2 was separated on silica gel CC with an isocratic elution of petroleum ether–acetone (1:1), yielding two components (C2a and C2b). Compounds **10** (58.6 mg, *t*_R_ = 28.8 min), **7** (7.3 mg, *t*_R_ = 29.7 min), and **24** (43.6 mg, *t*_R_ = 39.2 min) were purified from fraction C2a using prep. HPLC (MeCN/H_2_O 25:75→100:0, 50 min, 4 mL/min).

Fraction D5 was separated using MPLC equipped with an MCI column, employing a gradient elution of MeOH in H_2_O (20:80, 40:60, 60:40, 80:20, 100:0), resulting in seven subfractions (D5a–D5g). Fractions D5b and D5d were purified using Sephadex LH-20 (MeOH), yielding three (D5b1–D5b3) and four (D5d1–D5d4) components. Compound **19** (4.7 mg, *t*_R_ = 23.8 min) was obtained from fraction D5b3 via prep. HPLC (MeCN/H_2_O 30:70→100:0, 50 min, 4 mL/min). Compound **13** (2.1 mg, *t*_R_ = 22.5 min) was obtained from fraction D5d1 via prep. HPLC (MeCN/H_2_O 34:66→58:42, 30 min, 4 mL/min).

Fractions F1 to F4 were subjected to Sephadex LH-20 (MeOH) chromatography, yielding three or four components each (F1a–F1c, F2a–F2c, F3a–F3c, and F4a–F4d). Compound **20** (30.2 mg, *t*_R_ = 28.5 min) was isolated from fraction F1b using prep. HPLC (MeCN/H_2_O 15:85→40:60, 70 min, 3 mL/min). Compounds **21** (34.3 mg, *t*_R_ = 35.0 min) and **28** (2.4 mg, *t*_R_ = 37.6 min) were obtained from fraction F2c via prep. HPLC with an isocratic elution of MeCN/H_2_O (30:70, 3 mL/min). Compound **27** (3.4 mg, *t*_R_ = 24.7 min) was obtained from fraction F3c using prep. HPLC (isocratic elution of MeOH/H_2_O 71:29, 7 mL/min). Compound **3** (4.9 mg, *t*_R_ = 35.1 min) was isolated from fraction F4b via prep. HPLC (MeCN/H_2_O 20:80→60:40, 55 min, 4 mL/min).

Fraction F5 was separated using MPLC equipped with an MCI gel column by a gradient eluent of MeOH in H_2_O (20:80, 40:60, 60:40, 80:20, 100:0), yielding seven subfractions (F5a–F5g). Fractions F5d, F5f, and F5g were dealt with Sephadex LH-20 (MeOH), resulting in three or five components each (F5d1–F5d3, F5f1–F5f5, and F5g1–F5g5). Compounds **16** (141.8 mg, *t*_R_ = 10.6 min), **17** (72.2 mg, *t*_R_ = 43.9 min), and **2** (16.6 mg, *t*_R_ = 44.6 min) were obtained from fraction F5d2 via prep. HPLC (isocratic elution of MeCN/H_2_O 42:58, 4 mL/min). Compound **4** (7.4 mg, *t*_R_ = 24.5 min) was obtained from fraction F5f1 using prep. HPLC (MeCN/H_2_O 38:62 → 70:30, 40 min, 4 mL/min). Compound **15** (8.7 mg, *t*_R_ = 16.5 min) was purified from fraction F5f4 using prep. HPLC (MeCN/H_2_O 62:38, 4 mL/min). Compound **18** (41.5 mg, *t*_R_ = 9.8 min) was isolated from fraction F5g2 using prep. HPLC (MeCN/H_2_O 60:40→100:0, 20 min, 4 mL/min). Compound **29** (2.3 mg, *t*_R_ = 12.5 min) was obtained from fraction F5g3 using prep. HPLC (MeCN/H_2_O 60:40→100:0, 20 min, 4 mL/min). Finally, compound **1** (1.3 mg, *t*_R_ = 17.5 min) was purified from fraction F5g5 using prep. HPLC (60:40→100:0, 20 min, 4 mL/min).

#### ﻿Liquid fermentation of potato dextrose broth (PDB)

The culture medium for fermenting *X.longipes* was prepared using potato (200 g), peptone from porcine meat (1.0 g), dextrose (20.0 g), KH_2_PO_4_ (3.0 g), and MgSO_4_ (1.5 g) dissolved in deionized H_2_O (1.0 L). A total of 20 L of the culture medium was inoculated with the *X.longipes* strain and incubated at 24 °C with shaking at 150 rpm on rotary shakers for 28 days in a dark environment. After 7 days of fermentation, 3-methyl-2-butenol was added to the broth at a 400 mg/L concentration.

Following fermentation, the 20.0 L broth was separated into supernatant and mycelia by centrifugation. The supernatant was concentrated under reduced pressure, and the resulting residue was partitioned between ethyl acetate (EtOAc) and H_2_O. Simultaneously, the mycelia were extracted three times with ethanol (EtOH). The EtOH extract was evaporated and similarly partitioned between EtOAc and H_2_O four times. The combined EtOAc layers yielded a total weight of 24 g.

The EtOAc extract underwent MPLC using a C18-ODS column, eluted by a gradient eluent of MeOH in H_2_O (20:80, 40:60, 60:40, 80:20, 100:0), yielding five fractions (A–E). Fractions B (5.6 g) and D (9.7 g) were separated over silica gel CC eluted with petroleum ether–acetone (2:1, 1:1, 0:1), giving twelve (B1–B12) and eight fractions (D1–D8). Compound **30** (178.6 mg, *t*_R_ = 27.9 min) was purified from fraction B2 via prep. HPLC (MeCN/H_2_O 34:66→69:31→100:0, 0→35→45 min, 4 mL/min).

Subfraction D4 was further purified by preparative prep. HPLC using a gradient eluent (MeCN/H_2_O 80:20→100:0, 45 min, 4 mL/min) to afford **31** (4.1 mg, *t*_R_ = 27.9 min) and **33** (1.3 mg, *t*_R_ = 40.5 min). Fraction D2 was subjected to prep. HPLC (MeCN/H_2_O 51:49→95:5, 35 min, 4 mL/min) gives twelve components (D2a–D2l). Compounds **34** (1.2 mg, *t*_R_ = 38.4 min) from fraction D2e (MeCN/H_2_O 60:40→100:0, 45 min, 4 mL/min) and **32** (4.0 mg, *t*_R_ = 43.5 min) from fraction D2h (MeCN/H_2_O 80:20→100:0, 45 min, 4 mL/min) via prep. HPLC.

#### ﻿Liquid fermentation of glucose peptone yeast (GPY)

A total of 45.0 L of the GPY culture medium was inoculated with *X.longipes* strain and was then incubated at 24 °C and 150 rpm on rotary shakers for 28 days in a dark environment. After fermentation for 7 days, 3-methyl-2-butenol was added (400 mg/L).

After a procedure similar to PDB fermentation, 45.0 L of GPY fermentation and mycelium gave an EtOAc layer (41.5 g). The EtOAc extract underwent macroporous adsorption resin CC eluted with an EtOH/H_2_O gradient (20:80, 40:60, 60:40, 80:20, 100:0), yielding four fractions (A–D). Fraction C (3.4 g) was separated on MPLC (C18-ODS column) and eluted with a MeOH/H_2_O gradient (20:80, 40:60, 60:40, 80:20, 100:0), yielding eight fractions (C1–C8). Fractions C3 (MeOH/H_2_O, 20:80→100:0, 30 min, 7 mL/min) and C6 (MeOH/H_2_O 50:50→100:0, 45.0 min, 7 mL/min) were further separated over prep. HPLC to provide eight (C3a–C3h) or three fractions (C6a–C6c), respectively. Compounds **37** (2.6 mg, *t*_R_ = 8.7 min) from fraction C3d (MeCN/H_2_O 22:78, 4 mL/min), **39** (4.6 mg, *t*_R_ = 14.5 min) from fraction C3h (MeCN/H_2_O 20:80→50:50, 30 min, 4 mL/min), **38** (2.4 mg, *t*_R_ = 10.4 min) from fraction C6b (MeCN/H_2_O 35:65→65:35→100:0, 0→35→50 min, 3 mL/min), and **41** (5.5 mg, *t*_R_ = 28.9 min) from fraction C6c (MeCN/H_2_O 35:65→65:35→100:0, 0→35→50 min, 4 mL/min) were purified by prep. HPLC. Fraction C5 was separated on MPLC (MCI gel column) eluted with a MeOH/H_2_O gradient (20:80, 40:60, 60:40, 80:20, 100:0), yielding six fractions (C5a–C5f). Compounds **36** (9.7 mg, *t*_R_ = 8.7 min) from fraction C5c (MeCN/H_2_O15:85→70:30→100:0, 0→30→60 min, 4 mL/min), **40** (4.4 mg, *t*_R_ = 35.3 min) from fraction C5e (MeCN/H_2_O 35:65→60:40→100:0, 0→60→80 min, 4 mL/min), and **28** (2.4 mg, *t*_R_ = 38.2 min)/**35** (2.4 mg, *t*_R_ = 51.2 min) from fraction C5g (MeCN/H_2_O 28:72→28:72→100:0, 0→60→70 min, 4 mL/min) were obtained via prep. HPLC. Fraction D (2.6 g) was subjected to silica gel CC, using a petroleum ether–acetone elution system (2:1, 1:1, 0:1), giving six fractions (D1–D6), and then subfraction D1 was dealt with prep. HPLC (MeCN/H_2_O 40:60→75:25→100:0, 0→30→45 min, 4 mL/min) to give **25** (4.5 mg, *t*_R_ = 23.5 min).

### ﻿Calculations of ^13^C NMR, ECD, and optical rotation

Conformational analysis of the candidate structures (compounds **1**, **5**, **6**, **12**–**14**) was performed using the MMFF94s force field. The conformers with a distribution greater than 1% were further optimized at the B3LYP/6-31G(d,p) level of theory in Gaussian 16. The optimized conformers within 3 kcal/mol of the global minimum were selected for additional ^13^C NMR (**1**, **12**, **13**), ECD (**1**, **5**, **6**, **12**, **13**), and optical rotation calculations (**14**). All details were listed in the Suppl. material 1.

The ^13^C NMR calculation. Gauge-independent atomic orbital (GIAO) calculations for the shielding values of candidates were accomplished by the Ground State method at the b3lyp/6-31G(d,p) level in CHCl_3_ with the CPCM model in Gaussian 16 (Frisch et al. 2016). The calculated NMR data of these conformers were averaged according to the Boltzmann distribution theory and their relative Gibbs free energies. The correlation coefficient (*R*^2^) and mean absolute error (MAE) were calculated to evaluate the deviations between the experimental and calculated results. An in-house Excel-based program processed the calculation results.

ECD calculation. The theoretical calculations of ECD were performed using the time-dependent density functional theory (TDDFT) at the B3LYP/6-31G(d,p) level in MeOH with the CPCM model in Gaussian 16. The calculated ECD curve was generated using SpecDis 1.70.1. The ECD curves of the enantiomers were generated by the function “enantiomeric ECD” in SpecDis (Bruhn et al. 2012).

Optical rotation calculation. The theoretical calculations of optical rotation were performed using the Ground State method at B3LYP/6-311G+(d,p) level in MeOH with the CPCM model in Gaussian 16. The average value of 3*R*-**14** was weighted using Excel.

### ﻿Bioassays

#### ﻿Assays of immunosuppressive activity, lymphocyte viability, and antiproliferative activity against HaCaT cells

As previously described, the immunosuppressive activity, lymphocyte viability, and antiproliferative activity against HaCaT cells were evaluated using the CCK-8 method (Zhao et al. 2023, 2024).

In the immunosuppressive and lymphocyte viability, the primary tested concentration of all compounds was 20.0 *μ*M, and dexamethasone (Dex) (*c* = 2.0 *μ*M) was used as a positive control. In the antiproliferative activity against HaCaT cells, the primary tested concentration of all compounds was 40 *μ*M, and the positive control was methotrexate (MTX) (*c* = 40.0 *μ*M). When the inhibition rates are greater than 50%, the IC_50_ values of active compounds will be further tested and calculated. All experiments were performed in triplicate.

#### ﻿Cytokine analysis by ELISA of induced T cells

The mononuclear cell suspensions (2 × 10^5^ cells/well) were cultured with ConA (5 *μ*g/mL) in 96-well plates, and indicated concentrations of compounds **2**, **15**, and **32** were added simultaneously. After a 48-hour culture period, cytokines in the supernatants were quantified using mouse IFN-*γ*, IL-2, and IL-17A ELISA kits (Mabtech, Sweden), following the manufacturer’s protocol (Jing et al. 2021; Zhao et al. 2024).

#### ﻿EdU proliferation assay

EdU fluorescence labeling for cell proliferation of induced T/B lymphocytes (2 × 10^5^ cells/well) and HaCaT cells (1 × 10^4^ cells/well) of active compounds **10**, **19**, **23**, **32**, **38**, and **41** (final concentration 10 or 20 *μ*M) was performed as previously reported (Biram and Shulman 2020; Zhao et al. 2024). EdU (HF488) (the Click-iT™ EdU Flow Cytometry Assay Kit, APExBIO, USA) was added 24 h (final concentration 50 *μ*M, T and B cells) and 4 h (final concentration 10 *μ*M, HaCaT cells) before harvesting the cells. All procedures were performed according to the manufacturer’s instructions for the click reaction. EdU-positive cells and proliferating phase cells were analyzed with a fluorescence microscope.

### ﻿Immunofluorescence protocol (cell climbing slides)

The immunofluorescence protocol was performed using the reported method (Zhao et al. 2024). HaCaT cells (2.0 × 10^5^ cells/well) were plated onto 6-well plates with glass coverslips and adhered overnight (12 h) before compound addition. After treatment with active compounds **10** (final concentration 31 *μ*M) and **32** (final concentration 27 *μ*M) for 36 h, the supernatant was removed, and the cells were washed twice with PBS, fixed with 4% paraformaldehyde for 15 min, and then washed with PBS three times. After blocking, incubated with NF-*κ*B p65 (p65)/phosphorylated NF-*κ*B p65 (p-p65) antibodies and secondary antibodies (Servicebio, China). Then, cells were counterstained with 4,6-diamino-2-phenylindole (DAPI) for 5 min. After the PBS washing, fluorescent seal liquid was added, and the plate was monitored under an imaging system (Pannoramic MIDI, 3Dhistech, Hungary). The nucleus is blue, and positive cells are green (p-p65) or red (p65). The immunofluorescence areas for each indicator were analyzed in ImageJ.

### ﻿Statistics and reproducibility

GraphPad Prism 8.0 software was used for statistical data analysis, which was expressed as 'X ± s. One-way ANOVA was used to compare the differences between groups. *P* < 0.05 or *P* < 0.01 was considered statistically significant.

#### ﻿Spectroscopic data of compounds

The ^1^H, ^13^C, 2D NMR, HR-ESI-MS spectra, and computational details of compounds **1**-**14** were listed in the Suppl. material 1.

#### ﻿11*α*,16-Epoxy-isopimar-8(9)-en-19*β*-oic acid (1)

White powder; $[\alpha {]}_{D}^{23}$ +12.2 (*c* 0.01, MeOH); UV (MeOH) *λ*_max_ (log *ε*): 201 (4.41), 214 (4.09) nm; IR (MeOH) *ν*_max_ cm^-1^: 3338, 2945, 1690, 2833, 1449, 1114, 1027; ^1^H NMR (500 MHz, CDCl_3_) data see Table 2; ^13^C NMR (125 MHz, CDCl_3_) data see Table 2; HR-ESI-MS *m/z* 319.2269 [M + H]^+^ (calcd 319.2268 for C_26_H_31_O_3_^+^).

**Table 2.** ^1^H (500 MHz) and ^13^C NMR (125 MHz) data of **1** (CDCl_3_).

pos.	*δ*_C_	*δ*_H_
1	36.0	2.23, m; 1.84*
2	19.3	1.84*; 1.52, m
3	37.8	2.19, br.d (13.9); 1.04, ddd (13.9, 13.9, 4.5)
4	43.7	
5	53.3	1.40, d (12.2)
6	20.5	2.00, m; 1.85, m
7	32.0	2.10, m; 1.90, m
8	134.7	
9	133.3	
10	38.2	
11	66.9	4.30, dd (2.5, 2.5)
12	40.1	1.71, dd (11.6, 3.5); 1.30, dd (11.6, 3.5)
13	27.6	
14	44.3	1.81, m, 2H
15	40.9	1.50, m; 1.19, m
16	58.5	3.61, ddd (13.1, 11.4, 3.1); 3.51, dd (11.4, 5.8)
17	31.8	0.92, s
18	28.7	1.27, s
19	181.2	
20	18.2	0.85, s

* overlapped

#### ﻿16-*α*-*D*-Mannopyranosyloxyisopimar-8(9)-en-19-oic acid (2)

Colorless oil; $[\alpha {]}_{D}^{23}$ +27.2 (*c* 0.06, MeOH); UV (MeOH) *λ*_max_ nm (log *ε*): 201 (3.91), 241 (3.58); IR (MeOH) *ν*_max_ cm^−1^: 3374, 2934, 1697, 1645, 1024; ^1^H NMR (500 MHz, DMSO-*d*_6_) data see Table 3; ^13^C NMR (125 MHz, DMSO-*d*_6_) data see Table 3; HR-ESI-MS *m/z* 505.2773 [M + Na]^+^ (calcd 505.2772 for C_26_H_42_O_8_Na^+^).

**Table 3.** ^1^H (500 MHz) and ^13^C NMR (125 MHz) data of **2** and **3** (DMSO-*d*_6_, *J* in Hz).

**pos.**	**2**		**3**	
	***δ*_C_**	***δ*_H_**	***δ*_C_**	***δ*_H_**
1	36.7	1.75, m; 1.01, m	36.6	1.75, m; 1.03, m
2	19.3	1.74, m; 1.42, m	19.3	1.76 *; 1.41, m
3	37.3	2.02, m; 0.97, m	37.3	2.03, m; 0.99, m
4	42.9		42.9	
5	52.6	1.29, d (11.8)	52.6	1.29, d (11.5)
6	20.5	1.85 *; 1.72, m	20.5	1.86 *; 1.72, m
7	32.8	1.85*; 1.80, m	32.8	1.86 *; 1.81, m
8	124.8		124.8	
9	135.0		135.0	
10	37.7		37.7	
11	20.3	1.90, m; 1.83, m	20.4	1.90, m; 1.83, m
12	34.1	1.44, m; 1.17, m	34.0	1.45, m; 1.16, m
13	30.2		30.0	
14	43.8	1.65, d (17.9); 1.60 d (17.9)	43.9	1.65, d (18.0); 1.60 d (18.0)
15	36.2	1.47, m; 1.34, m	36.4	1.49, m; 1.36, m
16	63.2	3.67*; 3.32*	63.6	3.65, m; 3.32, m
17	27.2	0.89, s	27.2	0.87, s
18	28.3	1.15, s	28.3	1.13, s
19	178.9		178.6	
20	17.5	0.85, s	17.5	0.84, s
1'	99.8	4.55, brs	98.6	4.58, d (3.4)
2'	70.4	3.55, brs	71.9	3.16, dd (9.3, 3.4)
3'	71.1	3.43*	73.3	3.37, t (9.3)
4'	67.0	3.36, t (9.3)	70.3	3.05, t (9.3)
5'	74.1	3.28, m	72.8	3.33*
6'	61.3	3.64, dd (11.6, 2.1);	61.0	3.60, brd (11.4);
		3.44, dd (11.6, 6.3)		3.43, dd (11.6, 3.0)

* overlapped

#### ﻿16-*α*-*D*-Glucopyranosyloxyisopimar-8(9)-en-19-oic acid (3)

White powder; $[\alpha {]}_{D}^{23}$ +88.9 (*c* 0.05, MeOH); UV (MeOH) *λ*_max_ nm (log *ε*): 201 (3.88), 242 (3.55); IR (MeOH) *ν*_max_ cm^−1^: 3288, 2933, 1696, 1457, 1034; ^1^H NMR (500 MHz, DMSO-*d*_6_) data see Table 3; ^13^C NMR (125 MHz, DMSO-*d*_6_) data see Table 3; HR-ESI-MS *m/z* 505.2770 [M + Na]^+^ (calcd 505.2772 for C_26_H_42_O_8_Na^+^)

#### ﻿Cyclo(*L*-Phe-*L*-Val-*D*-Ile-*L*-Leu-*L*-Pro) (4)

White powder; $[\alpha {]}_{D}^{23}$ −64.4 (*c* 0.03, MeOH); UV (MeOH) *λ*_max_ nm (log *ε*): 209 (3.72), 244 (3.12), 307 (2.90); IR (MeOH) *ν*_max_ cm^−1^: 3275, 2960, 1634, 1541, 1445; ^1^H NMR (500 MHz, DMSO-*d*_6_) data see Table 4; ^13^C NMR (125 MHz, DMSO-*d*_6_) data see Table 4; HR-ESI-MS *m/z*: 568.3499 [M−H]^−^ (calcd 568.3504 for C_31_H_46_O_5_N_5_^−^).

**Table 4.** ^1^H (500 MHz) and ^13^C NMR (125 MHz) data of **4** (DMSO-*d*_6_, *J* in Hz).

Amino acid	pos.	*δ*_C_	*δ*_H_
*L*–phenylalanine	CO	170.5	
	*α*	54.2	4.37, dd (15.5, 7.9)
	*β*	36.6	2.84, m
	*γ*	137.5	
	*ortho*	129.2	7.17, m
	*meta*	128.0	7.24, m
	*para*	126.2	7.24, m
	NH		8.42, d (7.9)
*L*–valine	CO	171.4	
	*α*	58.2	3.83, t (9.2)
	*β*	26.1	1.94, m
	*γ*	19.7 18.4	0.80, d (5.0); 0.62, d (6.6)
	NH		8.35, d (9.2)
*D*–isoleucine	CO	170.2	
	*α*	55.7	4.19, dd (8.5, 7.1)
	*β*	37.8	1.52, m
	*γ*	25.6	1.27, m; 1.01, m
	*σ*	11.5	0.83, d (6.2)
	*β*–CH_3_	14.4	0.72, d (6.9)
	NH		7.07, d (8.5)
*L*–leucine	CO	168.8	
	*α*	47.6	4.61, dd (14.5, 8.6)
	*β*	41.5	1.51, m; 1.41, m
	*γ*	24.4	1.35, m
	*σ*	22.7 22.4	0.85, d (5.4) 0.82, d (5.3)
	NH		8.34, d (8.6)
*L*–proline	CO	170.4	
	*α*	59.9	4.67, d (7.8)
	*β*	32.0	1.94, m; 1.61, m
	*γ*	20.9	1.65, m; 1.41, m
	*σ*	45.6	3.28, m

#### ﻿Diplosporin A (5)

Yellow oil; $[\alpha {]}_{D}^{23}$ +47.9 (*c* 0.15, MeOH); UV (MeOH) *λ*_max_ nm (log *ε*): 211 (5.58), 251 (5.59); IR (MeOH) *ν*_max_ cm^−1^: 3396, 2937, 1660, 1611, 1456, 1106, 1032; ^1^H NMR (500 MHz, CDCl_3_) data see Table 5; ^13^C NMR (125 MHz, CDCl_3_) data see Table 6; HR-ESI-MS *m/z* 261.1097 [M + Na]^+^ (calcd 261.1103 for C_13_H_18_O_4_Na^+^).

**Table 5.** ^1^H (500 MHz) data of **5**−**9** (*J* in Hz).

pos.	5^a^	6^b^	7^b^	8^b^	9^b^
2	7.99, brs	7.79, brs	7.72, brs	7.71, brs	7.69, d (5.7)
3					6.30, d (5.7)
5	4.83, d (2.2)	4.59, d (7.7)	4.51, d (1.7)	4.27, d (7.6)	4.57, d (7.7)
6	1.37, m	1.64, m	1.32, m	1.97, m	1.63, m
7	1.77, m	2.03, m; 1.45, m	1.81, ddd (12.9, 12.2, 6.7); 1.72, m	2.15, m; 1.75, m	2.03, m; 1.46, m
8	2.66, m	2.66, m; 2.55, m	2.63, dd (18.6, 6.7); 2.56, dd (11.4, 7.0)	2.48, m	2.64, m; 2.54, dt (18.2, 4.8)
9	4.31, d (13.7); 4.27, d (13.7)	4.33, d (13.5); 4.29, d (13.5)	4.34, d (13.0); 4.30, d (13.0)	4.35, d (13.6); 4.27, d (13.6)	
10	1.63, m; 1.41, m	1.85, m; 1.28, m	1.64, m; 1.44, m	1.21, m; 1.13, m	1.84, m; 1.28, m
11	1.04, t (7.3)	0.98, t (7.3)	1.00, t (7.4)	0.97, t (7.4)	0.98, t (7.5)
12	3.40, s	3.44, s	3.44, s	3.44, s	
13			3.54, s	3.54, s	

^a^CD_3_OD; ^b^ CDCl_3_

**Table 6.** ^13^C NMR (125 MHz) data of **5**−**13**.

pos.	5^a^	6^b^	7^b^	8^b^	9^b^	10^b^	11^b^	12^b^	13^b^
2	155.2	152.8	152	152	155	151.3	153.5	152.6	152.7
3	126.0	125.2	125.3	125.4	116.5	125.5	121.4	125.2	125.1
4	179.2	179.5	177.6	177.6	180.3	174.5	177.2	178.2	178.6
4a	125.2	123.5	123.6	121.0	125.0	120.1	123.8	123.5	122.1
5	62.3	69.0	70.8	73.4	69.0	111.4	124.0	64.3	68.8
6	42.0	41.5	41.2	36.4	41.4	140.4	141.5	42.3	72.4
7	22.7	23.3	21.8	19.8	23.3	26.9	134.0	21.6	27.9
8	28.8	27.0	27.9	23.3	27.0	26.2	118.1	27.2	24.8
8a	168.8	165.6	166.6	166.0	165.7	162.4	155.1	165.6	165.6
9	67.0	66.1	66.5	66.5		66.6	66.6	66.2	66.2
10	25.3	25.3	24.6	22.0	24.5	29.8	28.5	138.2	29.3
11	11.9	11.3	12.1	12.2	11.3	12.1	15.7	116.1	6.8
12	58.9	59.2	59.1	59.2		59.1	59.0	59.2	59.2
13			60.2	58.1					

^a^CD_3_OD; ^b^ CDCl_3_

#### ﻿Diplosporin B (6)

White powder; $[\alpha {]}_{D}^{23}$ +41.3 (*c* 1.21, MeOH); UV (MeOH) *λ*_max_ nm (log *ε*): 213 (3.90), 254 (3.91); IR (MeOH) *ν*_max_ cm^−1^: 3418, 2947, 1658, 1592, 1457, 1108, 1030; ^1^H NMR (500 MHz, CDCl_3_) data see Table 5; ^13^C NMR (125 MHz, CDCl_3_) data see Table 6; HR-ESI-MS *m/z* 261.1093 [M + Na]^+^ (calcd 261.1103 for C_13_H_18_O_4_Na^+^).

#### ﻿Diplosporin C (7)

Yellow powder; $[\alpha {]}_{D}^{23}$ +11.8 (*c* 0.37, MeOH); UV (MeOH) *λ*_max_ nm (log *ε*): 222 (3.76), 245 (3.77); IR (MeOH) *ν*_max_ cm^−1^: 2934, 2876, 2828, 1660, 1620, 1435, 1088; ^1^H NMR (500 MHz, CDCl_3_) data see Table 5; ^13^C NMR (125 MHz, CDCl_3_) data see Table 6; HR-ESI-MS *m/z* 275.1253 [M + Na]^+^ (calcd 275.1259 for C_14_H_20_O_4_Na^+^).

#### ﻿Diplosporin D (8)

Yellow oil; $[\alpha {]}_{D}^{23}$ −25.6 (*c* 0.10, MeOH); UV (MeOH) *λ*_max_ nm (log *ε*): 212 (3.75), 253 (3.74); IR (MeOH) *ν*_max_ cm^−1^: 2928, 1662, 1622, 1435, 1308, 1197, 1086, 949; ^1^H NMR (500 MHz, CDCl_3_) data see Table 5; ^13^C NMR (125 MHz, CDCl_3_) data see Table 6; HR-ESI-MS *m/z* 253.1435 [M + H]^+^ (calcd 253.1440 for C_14_H_21_O_4_^+^).

#### ﻿Diplosporin E (9)

Yellow powder; $[\alpha {]}_{D}^{23}$ +40.7 (*c* 0.04, MeOH); UV (MeOH) *λ*_max_ nm (log *ε*): 210 (3.63), 251 (3.70); IR (MeOH) *ν*_max_ cm^−1^: 3364, 2960, 1650, 1600, 1438, 1144, 1020; ^1^H NMR (500 MHz, CDCl_3_) data see Table 5; ^13^C NMR (125 MHz, CDCl_3_) data see Table 6; HR-ESI-MS *m/z* 217.0838 [M + Na]^+^ (calcd 217.0841 for C_11_H_14_O_3_Na^+^).

#### ﻿Diplosporin F (10)

Yellow oil; UV (MeOH) *λ*_max_ nm (log *ε*): 229 (3.86), 282 (3.73); IR (MeOH) *ν*_max_ cm^−1^: 1659, 1636, 1605, 1451, 1202, 1114; ^1^H NMR (500 MHz CDCl_3_) data see Table 7; ^13^C NMR (125 MHz, CDCl_3_) data see Table 6; HR-ESI-MS *m/z* 243.0988 [M + Na]^+^ (calcd 243.0997 for C_13_H_16_O_3_Na^+^).

**Table 7.** ^1^H (500 MHz) data of **10**−**13** (CDCl_3_, *J* in Hz).

No.	10	11	12	13
2	7.71, s	7.95, s	7.78, s	7.78, s
5	6.37, s	8.03, d (2.2)	4.93, brs	4.62, s
6	/	/	2.39, m	/
7	2.39, t (9.2); 2.75, t (9.2)	7.51, dd (8.6, 2.2)	2.05, m; 1.85, m	1.96, m; 1.80, m
8	2.75, t (9.2)	7.38, d (8.6)	2.67, dd (6.0, 3.4); 2.62, dd (10.3, 6.2)	2.81, m; 2.51, m
9	4.34, brs	4.43, d (1.0)	4.33, brd (13.5); 4.29, brd (13.5)	4.33, brd (13.6); 4.29, brd (13.6)
10	2.17, dd (7.3, 7.1)	2.75, q (7.6)	6.08, m	1.89, m; 1.58, m
11	1.09, t (7.4)	1.28, t (7.6)	5.22, dd (1.3,1.3); 5.19, dd (5.3, 1.3)	1.04, t (7.5)
12	3.46, s	3.48, s	3.46, s	3.45, s

#### ﻿Diplosporin G (11)

Yellow powder; UV (MeOH) *λ*_max_ nm (log *ε*): 205 (3.57), 227 (3.70), 251 (3.35), 310 (3.09); IR (MeOH) *ν*_max_ cm^−1^: 2966, 2932, 1647, 1615, 1483, 11452, 1313, 1099, 828; ^1^H NMR (500 MHz, CDCl_3_) data see Table 7; ^13^C NMR (125 MHz, CDCl_3_) data see Table 6; HR-ESI-MS *m/z* 219.1018 [M + H]^+^ (calcd 219.1021 for C_13_H_15_O_3_^+^).

#### ﻿Diplosporin H (12)

Yellow powder; $[\alpha {]}_{D}^{23}$ +74.1 (*c* 0.16, MeOH); UV (MeOH) *λ*_max_ nm (log *ε*): 223 (3.69), 242 (3.70); IR (MeOH) *ν*_max_ cm^−1^: 1708, 1647, 1598, 1476, 1243, 1084, 1025; ^1^H NMR (500 MHz, CDCl_3_) data see Table 7; ^13^C NMR (125 MHz, CDCl_3_) data see Table 6; HR-ESI-MS *m/z* 259.0940 [M + Na]^+^ (calcd 259.0941 for C_13_H_16_O_4_Na^+^).

#### ﻿Diplosporin I (13)

Yellow oil; $[\alpha {]}_{D}^{23}$ −35.4 (*c* 0.01, MeOH); UV (MeOH) *λ*_max_ nm (log *ε*): 213 (3.74), 253 (3.74); IR (MeOH) *ν*_max_ cm^−1^: 3377, 1653, 1582, 1033, 991; ^1^H NMR (500 MHz, CDCl_3_) data see Table 7; ^13^C NMR (125 MHz, CDCl_3_) data see Table 6; HR-ESI-MS *m/z* 255.1223 [M + H]^+^ (calcd 255.1227 for C_13_H_19_O_5_^+^).

#### ﻿(*R*)-8-methoxy-3-(methoxymethyl)isochroman-1-one (14)

White powder; $[\alpha {]}_{D}^{23}$ +96.2 (*c* 0.02, MeOH); UV (MeOH) *λ*_max_ nm (log *ε*): 209 (3.72), 244 (3.12), 307 (2.90); IR (MeOH) *ν*_max_ cm^−1^: 1708, 1647, 1598, 1476, 1243, 1084, 1025; ^1^H NMR (500 MHz, CDCl_3_) data see Table 8; ^13^C NMR (125 MHz, CDCl_3_) data see Table 8; HR-ESI-MS *m/z* 223.0974 [M + H]^+^ (calcd 223.0965 for C_12_H_15_O_4_^+^).

**Table 8.** ^1^H (500 MHz) and ^13^C NMR (125 MHz) data of **14** (CDCl_3_, *J* in Hz).

No.	*δ*_C_	*δ*_H_
1	162.1	
3	76.3	4.55, m
4	31.5	2.90, dd (16.1, 2.9); 3.06, dd (16.1, 11.6)
4a	141.7	
5	119.5	6.82, d (8.0)
6	134.7	7.46, dd (8.0, 8.0)
7	111.1	6.92, d (8.0)
8	161.4	
8a	114.8	
9	73.6	3.62, dd (10.3, 5.4); 3.70, dd (10.3, 4.9)
10	59.7	3.44, s
11	56.3	3.94, s

## ﻿Results and discussion

### ﻿Identification of the strain

**Figure 3.**
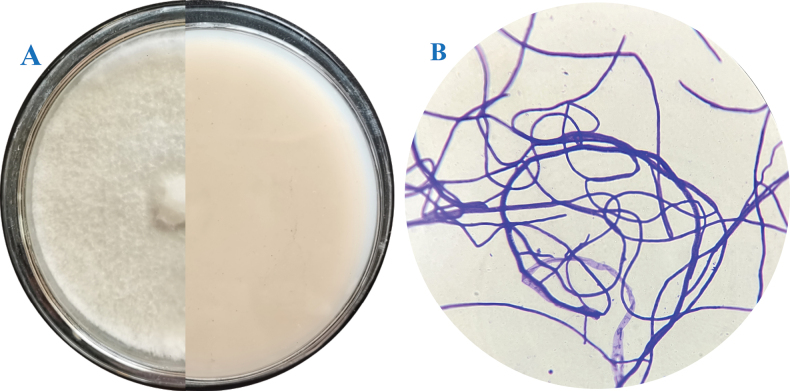
The phenotypical and morphological characters of *Xylarialongipes* HFG1018. **A** the colony; **B** methylene blue-stained mycelium (100 ×).

The strain of *Xylarialongipes* HFG1018 was identified through genetic analysis and morphological characterization. Colonies of *Xylarialongipes* HFG1018 were cultivated on OA at 25 °C for 4 weeks, reaching a diameter of 5 cm, with a white, velvety or inflorescence appearance, appressed with entire margins; the reverse side was white. No conidia were observed (Fig. 3). This fungus does not develop reproductive structures under culture conditions, which is consistent with the description in the reference (U’Ren et al. 2016).

**Figure 4.**
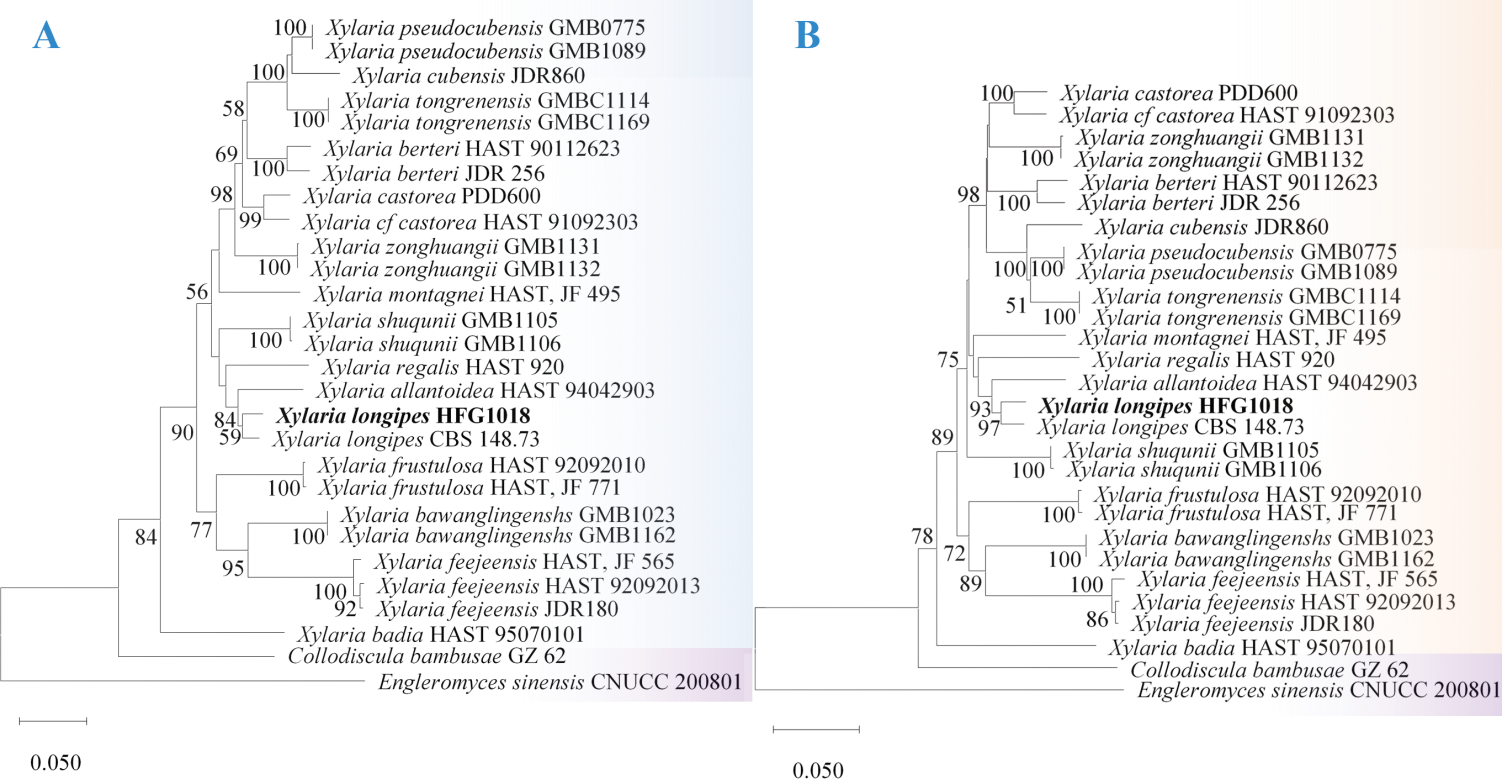
Maximum likelihood trees of *Xylaria* constructed using internal transcribed spacer (*ITS*), *β*-tubulin gene (*TUB2*), and DNA-directed RNA Polymerase II subunit 2 (*rpb2*) gene sequence data sets. Bootstrap support values of maximum likelihood (MLBP) above 50% are shown at the nodes. The tree was rooted to *Engleromycessinensis* CNUCC 200801. The strain *Xylarialongipes* described species are marked in bold. **A**ML tree; **B**NJ tree.

We conducted sequence analyses of the *TUB2*, *ITS*, and *rpb2* loci for molecular identification using the BLAST algorithm in NCBI. The results demonstrated the highest sequence homology with the corresponding gene sequences of *X.longipes*. The *TUB2* gene sequence of this strain had been previously characterized and deposited in GenBank (GenBank accession No. MN583256; Chen et al. 2020a, 2020b), while both the *ITS* region and *rpb2* gene sequence were newly determined in this study (GenBank accession Nos. PV523290 and PV558833, respectively). The *ITS* sequence exhibited 96.59% identity with *Xylariaellisii* (GenBank accession No. NR_172972.1; Ibrahim et al. 2020), confirming the genus-level classification of strain HFG1018 as *Xylaria*. Comparative sequence analysis revealed 96.4% and 97.28% identity for *TUB2* and *rpb2*, respectively, with reference sequences of *X.longipes* CBS 148.73 (GenBank accession Nos. KU684204.1 and KU684280.1; Franco et al. 2021; U’Ren et al. 2016) (Suppl. material 1: table S1). Phylogenetic reconstruction using MEGA v.12 software (employing both maximum likelihood and neighbor-joining methods) consistently placed isolate HFG1018 in a well-supported clade with the reference strain *X.longipes* CBS 148.73 (Fig. 4). These molecular analyses conclusively identify isolate HFG1018 as *X.longipes*.

### ﻿Structure elucidation of previously undescribed fungal metabolites (1–14)

This study identified 41 secondary metabolites from the ethyl acetate (EtOAc) fractions of three fermentations of *X.longipes*. Among these, compounds **1**–**14** were found to be previously undescribed derivatives (Figs 1, 2).

**Figure 5.**
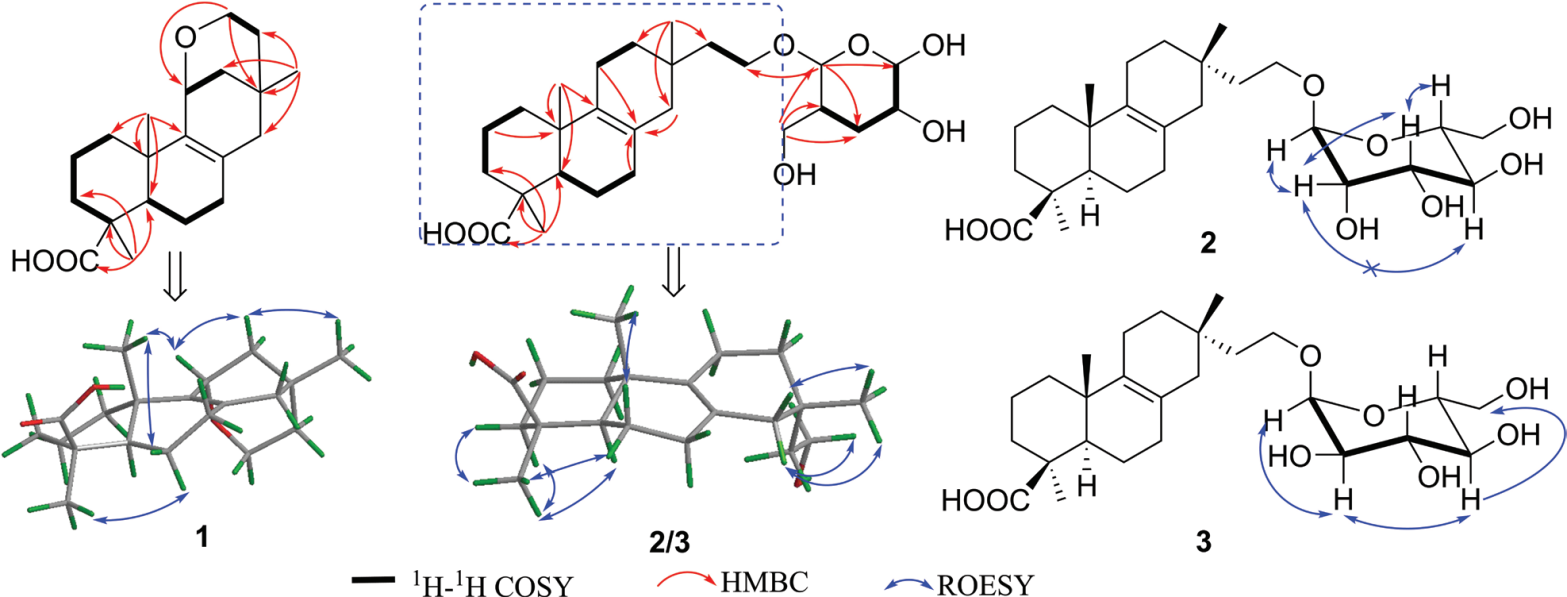
Key 2D NMR correlations of **1**–**3**.

Compound **1** was isolated as a white powder, and its molecular formula was determined to be C_26_H_30_O_3_ based on HRESIMS data, showing an ion at *m/z* 319.2269 [M + H]⁺ (calculated 319.2268 for C_26_H_31_O_3_^+^), indicating six degrees of unsaturation. The maximum UV absorption wavelength is 214 nm, indicating the absence of a large conjugated system in the structure of **1**. An obvious strong IR absorption at 1027 cm^-1^ indicates the existence of an ether bond. The ^1^H NMR spectrum exhibited signals for three methyl singlets (*δ*_H_ 0.85, 0.92, 1.27) and three protons attached to oxygenated carbons (*δ*_H_ 4.30, 3.61, 3.51) (Table 2). The ¹³C NMR spectrum revealed twenty carbon signals, including three methyls (*δ*_C_ 18.2, 28.7, 31.8), eight sp³ methylenes (*δ*_C_ 19.3, 20.5, 32.0, 36.0, 37.8, 40.1, 40.9, 44.3), one sp^3^ oxygenated methylene (*δ*_C_ 58.5), one sp³ methine (*δ*_C_ 53.3), one sp³ oxygenated methine (*δ*_C_ 66.9), three sp^3^ quaternary carbons (*δ*_C_ 27.6, 38.2, 43.7), a tetrasubstituted double bond (*δ*_C_ 133.3, 134.7), and one carbonyl (*δ*_C_ 181.2) (Table 2). The NMR data of **1** closely resemble those of the co-isolated 16-hydroxyisopimar-7-en-19-oic acid (**15**) (Wu et al. 2011), suggesting that **1** is an isopimarane-type diterpenoid (Fig. 1). The presence of a carboxyl group (COOH-19) and an oxymethylene group (CH_2_O-16) was confirmed by HMBC correlations (CH_3_-18 to C-19) and ¹H-¹H COSY correlations (H_2_-15 to H_2_-16), consistent with the isopimarane family from *Xylaria* species (Shiono et al. 2009; Wang et al. 2021; Zhou et al. 2021). HMBC correlations (H_3_-20 to C-9) and ^1^H-^1^H COSY correlations (H-11 to H_2_-12) indicated an oxygenated carbon at C-11 (*δ*_C_ 66.9) and a double bond at C-8(9) (Fig. 5). While three rings, one carboxyl, and one double bond accounted for five degrees of unsaturation, the remaining degree required an additional ring, a ring being formed through dehydration condensation. Among the possible positions (OH-16, OH-11, and COOH-19), COOH-19 was ruled out due to its distance from both OH-16 and OH-11, as well as its typical chemical shift (*δ*_C_ 181.2) for a carboxyl group (Chen et al. 2020b; Shiono et al. 2009; Zhou et al. 2021). Thus, it was proposed that the ring forms between OH-11 and OH-16. This was supported by a key HMBC correlation (H-16 to C-11) (Fig. 5) and further confirmed by a ^13^C NMR comparison with those of xylarinorditerpene I, an 18-nor-isopimarane containing a cyclohexyl ether between C-11 and C-13, which was previously isolated from the same fungus. (Chen et al. 2020b). The chemical shifts of the oxygenated methine at C-11 [*δ*_C/H_ 66.9/4.30 (dd, *J* = 2.5, 2.5 Hz)] and the oxygenated methylene at C-16 [*δ*_C/H_ 58.5/3.61 (ddd, *J* = 13.1, 11.4, 3.1 Hz); 3.51 (dd, *J* = 11.4, 5.8 Hz)] in compound **1** matched those of xylarinorditerpene I [*δ*_C/H_ 66.4/4.34 (dd, *J* = 2.5, 2.5 Hz), -CHO-11; *δ*_C/H_ 58.6/3.67 (ddd, *J* = 13.2, 11.6, 2.9 Hz) and 3.54 (dd, *J* = 11.6, 6.1 Hz), -CH_2_O-16].

**Figure 6.**
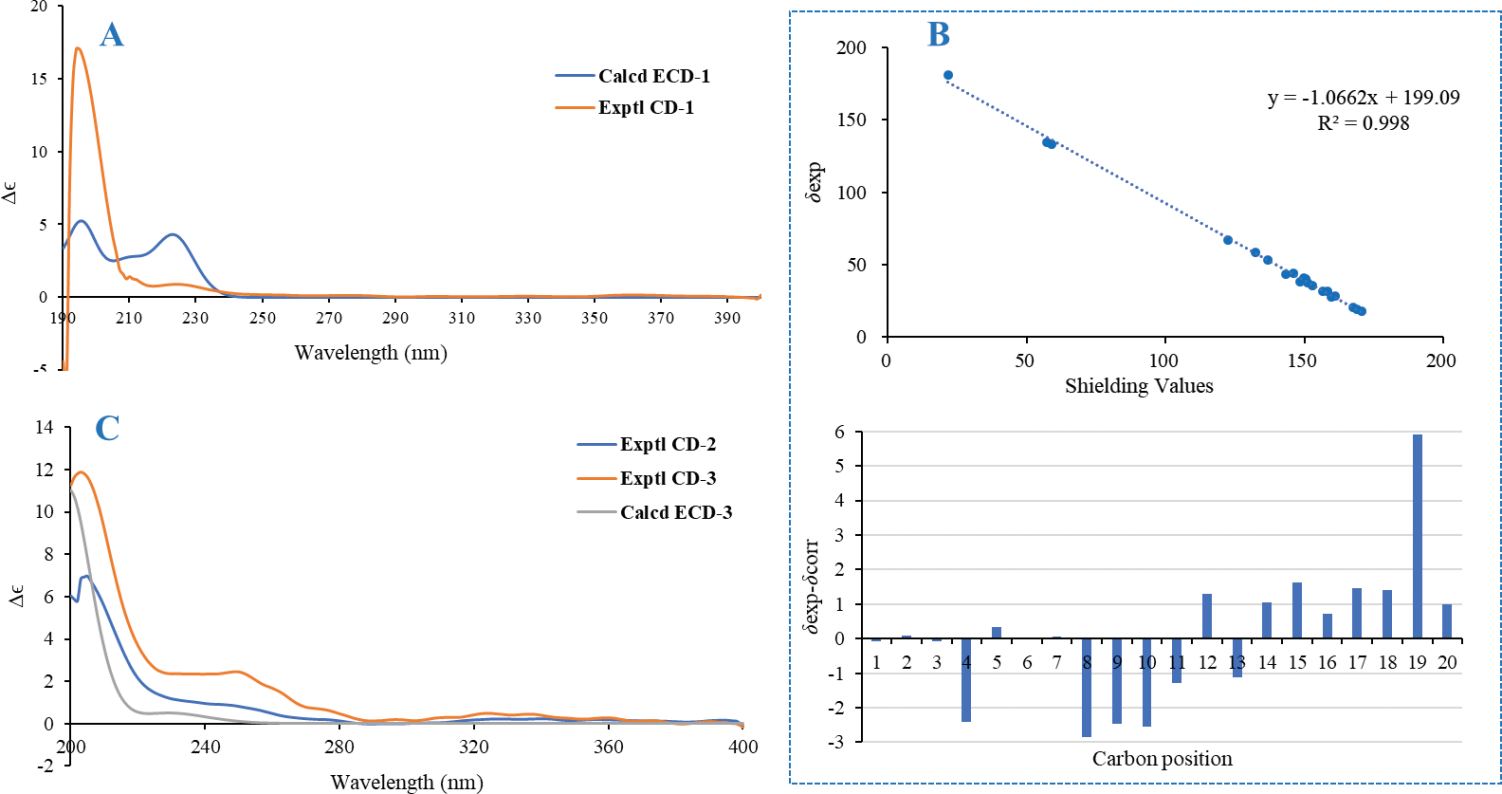
**A** comparison of the calculated ECD spectrum for **1** (*σ* = 0.22 eV and UV shift 5 nm) with its experimental CD spectrum in MeOH; **B** comparison of the calculated ^13^C NMR data for **1** with its experimental spectrum in CDCl_3_; **C** comparison of the calculated ECD spectrum for **2** and **3** (*σ* = 0.30 eV and UV shift 7 nm) with their experimental CD spectra in MeOH.

The relative configuration of **1** was determined using ROESY data (Fig. 5). The cross-peaks of H_3_-18/H-5 and H_3_-18/H-6*α* (*δ*_H_ 2.00) indicated their *α*-orientation. In contrast, cross-peaks of H_3_-20/H-11, H_3_-20/H-6*β* (*δ*_H_ 1.85), H-11/H-12*β* (*δ*_H_ 1.71), and H-12*β*/H_3_-17 suggested an 11*β*-H and a 17*β*-CH_3_. Finally, the absolute configuration and structural validity of **1** were confirmed by comparing experimental ECD and ^13^C NMR data with calculated spectra using the Gaussian 16 program. The calculated results matched well with the experimental data (Fig. 6A, B), assigning **1** as 11*α*,16-epoxy-isopimar-8(9)-en-19*β*-oic acid.

Compound **2** was isolated as a colorless oil. Its molecular formula, C_26_H_42_O_8_, matched that of co-isolate **16**, indicating six degrees of unsaturation. The ^1^H NMR data of **2** (Table 3) and HSQC spectrum exhibited characteristic resonances of three methyl singlets (*δ*_H_ 0.85, 0.89, and 1.15). Additionally, the distinctive signal of an anomeric proton at *δ*_H_ 4.55 (1H, brs), alongside eight protons ranging from *δ*_H_ 3.28 to 3.64, suggested the presence of a hexose unit and an oxygenated methylene group. The ^13^C NMR (Table 3) and DEPT spectra confirmed the presence of twenty-six carbon atoms, assigned as three methyls, ten methylenes (one oxygenated at *δ*_C_ 63.2), one methine, three sp^3^ quaternary carbon atoms, two olefinic quaternary carbons (*δ*_C_ 124.8 and 135.0), a carbonyl (*δ*_C_ 178.9), and six carbon atoms from the glycosyl moiety. Overall, the NMR data of **2** closely resembled those of 16-*α*-D-mannopyranosyloxyisopimar-7-en-19-oic acid (**16**) (Shiono et al. 2009). The only difference between **2** and **16** was the location of the double bond. HMBC correlation from H_3_-20 to C-9 indicated that the double bond was located at C-8(9) in **2** instead of C-7(8) in **16**. The ROESY correlations of H_3_-18/H-5, H_3_-18/H_2_-3, H_3_-18/H-6*α* (*δ*_H_ 1.85), and H_3_-20/H-6*β* (*δ*_H_ 1.72) showed that H_3_-18 was *α*-oriented while H_3_-20 was *β*-oriented. Moreover, ROESY correlations of H_3_-17/H_2_-14 (*δ*_H_ 1.65, 1.60) and H-16*β* (*δ*_H_ 3.67)/H-14α (*δ*_H_ 1.65) were observed, suggesting that H_3_-17 was *β*-oriented (Fig. 5).

According to the literature (Shiono et al. 2009; Wang et al. 2021; Zhou et al. 2021), only two pyranoses consisting of isopimarane from the *Xylaria* species have been identified: *α*-D-mannose and *α*-D-glucose, which can be assigned easily by comparing NMR analysis. In this work, compounds **2**, **16**, and **17** were obtained simultaneously, with the weights of **16**, which has an *α*-D-mannose moiety, and **17**, which has an *α*-D-glucose moiety, being larger than that of **2**. Their NMR data, measured in DMSO-*d*_6_, were carefully analyzed. The ^13^C NMR data of the pyranoses in **2** were nearly identical to those of **16**, with the largest deviation (Δ_max_) of 0.05 ppm at C5’ (Suppl. material 1: fig. S90), indicating that the pyranose in **2** is *α*-D-mannose. Additionally, observed ROESY correlations of H-1’/H-2’/H-3’/H-5’ and unobserved ROESY correlations of H-2’/H-4’ followed the configuration of *α*-D-mannose. Therefore, the structure of **2** was determined as 16-*α*-D-mannopyranosyloxyisopimar-8(9)-en-19-oic acid.

Compound **3** was isolated as a colorless oil. The HRESIMS spectra of **3** suggested a molecular formula of C_26_H_42_O_8_, identical to those of compounds **2**, **16**, and **17**. The ^1^H and ^13^C NMR data of **3** were extremely similar to those of **2**, except for the resonances of the glycosyl at C-1’−C-6’ (**2**: *δ*_C_ 99.8, 70.4, 71.1, 67.0, 74.1, 61.3; **3**: *δ*_C_ 98.6, 71.9, 73.3, 70.3, 72.8, 61.0) (Table 3). Further analysis of the 2D NMR data confirmed that compounds **2** and **3** shared the same planar structure but differed in glycosyl (Fig. 5). Considering the similar biosynthetic pathways, coupling constant (*J* value) of H-1’ (3.4 Hz), ROESY correlations (H-1’/H-2’/H-4’/H_2_-6’) (Fig. 3), and comparing the chemical shifts of C-1’−C-6’ of **3** to those of **17** [the Δ_max_ is 0.13 ppm (C1’)] (Suppl. material 1: fig. S90), this pyranose in **3** was deduced to be *α*-D-glucose. The remaining configurations of **3** were determined to be the same as those of **2** due to their identical key ROESY correlations (Fig. 5). Thus, the structure of **3** is 16-*α*-D-glucopyranosyloxyisopimar-8(9)-en-19-oic acid.

As illustrated above, compounds **2** and **3** exhibit identical planar structures and nearly identical configurations, with the only difference being the orientation of the 4’-OH group in the glycosyl moiety. Consequently, their circular dichroism (CD) spectra display similar trends to their calculated electronic circular dichroism (ECD) spectra. Only the ECD spectrum of **3** was calculated and compared with the experimental spectra of **2** and **3**. As shown in Fig. 6C, the diterpenoid moieties of **2** and **3** were determined to have the absolute configurations (5*R*, 9*S*, 10*R*, 13*S*).

**Figure 7.**
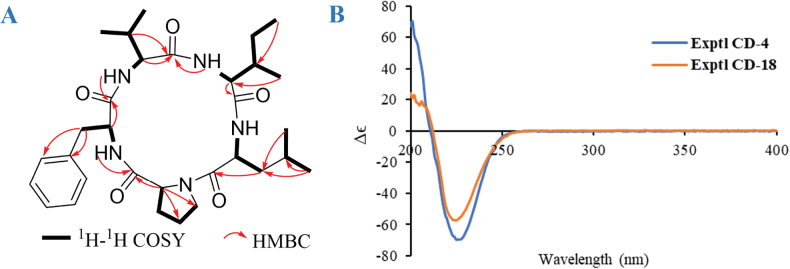
**A** key HMBC and ^1^H-^1^H COSY correlations of **4**; **B** comparison of the experimental CD spectra between **4** and **18** in MeOH.

Compound **4**, isolated as a colorless powder, had a molecular formula of C_31_H_46_O_5_N_5_ based on its HRESIMS and comprised 14 mass units (CH_2_) less than compound **18** (Wu et al. 2011). Analysis of the ^1^H, ^13^C, DEPT, and HSQC NMR spectroscopic data of **4** (Table 4) revealed the presence of three amide protons (*N*-H *δ*_H_ 7.07, 8.34, 8.35, and 8.42, respectively), six methyl groups (including five methyl singlets and one methyl triplet), six methylenes (including one *N*-methylene), eight methines (five of which are heteroatom-bonded), one monosubstituted benzene, and five carboxylic carbons. Analysis of the ^1^H and ^13^C NMR spectroscopic data of **4** (Table 4) revealed its structural features similar to those of **18**, except that *N*-H replaced the signals for the *N*-Me. These observations were confirmed by relevant ^1^H-^1^H COSY and HMBC correlations (Fig. 7). By using COSY and HSQC, the independent spin systems of the type X-CH(NH)-CH_2_-CH_2_-CH_2_-X’, X-CH(NH)-CH(CH_3_)_2_, X-CH(NH)-CH(CH_3_)-CH_2_-CH_3_, and X-CH(NH)-CH_2_-CH(CH_3_)_2_ were identified, indicating the presence of proline, valine, isoleucine, and leucine residues. The remaining independent spin system of the type X-CH(NH)-CH_2_-X’, together with the HMBC correlations from *β*H (Phe) to the *γ* and ortho carbons of the benzene and from H-*N* (*δ*_H_ 8.42) to CO (*δ*_C_ 170.5, Phe), established the phenylalanine (Phe) unit. Because only 10 of the calculated 11 degrees of unsaturation could be accounted for, it became clear that **4** is a cyclic peptide. Upon extensive analysis of these data, compound **4** was assigned as a cyclic pentapeptide containing one equivalent each of valine, leucine, isoleucine, phenylalanine, and proline. The carbonyl carbons within each residue were assigned from HMBC correlations between the C=O and their respective *α* and *N* protons (Table 4 and Fig. 7A), which established the complete amino acid sequence of **4** as cyclo-(-Phe-Pro-Leu-Ile-Val-). Since the optical rotation and experimental CD spectrum of **4**$[\alpha {]}_{D}^{23}$ −64.4) were quite similar to those of **18**$[\alpha {]}_{D}^{23}$ −46.8) (Wu et al. 2011) (Fig. 7B), it suggested that their amino acid compositions should be identical. However, this evidence remained insufficient. Therefore, a comparative analysis of the NMR data between **4** and **18** was further conducted. Given that NMR signals are closely associated with the atomic chemical environment, a high degree of consistency in NMR data would indicate that the two compounds share the same planar structure and relative configuration. The comparative results demonstrated that the ^1^H NMR and ^13^C NMR spectra of **4** and **18** (both measured in DMSO-*d*_6_) were remarkably similar. Only two notable discrepancies were observed in the ^13^C NMR spectra: Carbonyl carbon of *L*-proline: 170.4 ppm for **4***vs.* 172.5 ppm for **18** (Δ*δ* = 2.1). *α*-Carbon of *L*-phenylalanine (**4**) and *L*-*N*-methyl phenylalanine (**18**): 54.2 ppm and 56.5 ppm, respectively (Δ*δ* = 2.3), likely due to methylation-induced chemical shift variations. The remaining carbon signals showed chemical shift deviations of no more than 0.8 ppm, and the relevant data have been included in the Suppl. material 1: table S2). Integrating the CD spectra, optical rotation values, NMR data, and biosynthetic pathway analysis, **4** (7.4 mg) was deduced as the demethylated derivative of **18** (41.5 mg), and the *L* configuration for Phe, Val, Ile, Leu, Pro, and D configuration for Ile.

**Figure 8.**
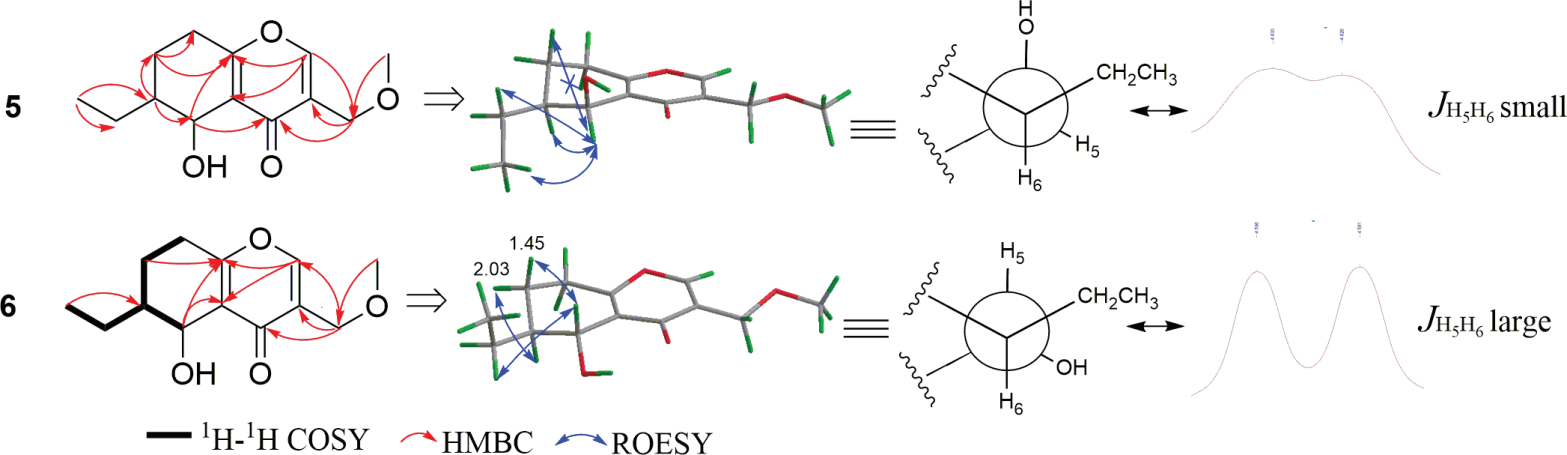
Key 2D NMR correlations, stereochemistry analysis, and ^1^H NMR spectral comparison of **5** and **6**.

**Figure 9.**
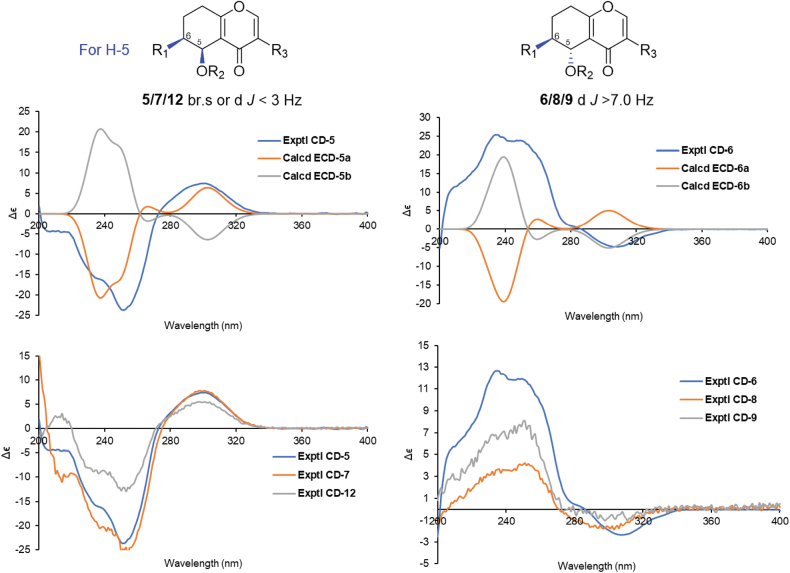
Comparison of the calculated ECD spectrum for **5** (σ = 0.20 eV, UV shift 7 nm), **6** (σ = 0.22 eV, UV shift 9 nm) with their experimental spectra in MeOH; empirical rules for configuration of diplosporins.

Compound **5** was isolated as a yellow oil. HRMS analysis confirmed its formula as C_13_H_18_O_4_ (indicating five double bond equivalents). Analysis of the ^1^H NMR spectrum of **5** (Table 5) identified 17 of the 18 protons, including an olefinic hydrogen (*δ*_H_ 7.99, brs), three hydrogens on oxygen carbons [*δ*_H_ 4.83 (d, *J* = 2.2 Hz), 4.31 (d, *J* = 13.7 Hz), 4.27 (d, *J* = 13.7 Hz)], five multiple protons (*δ*_H_ 1.37−2.66), a triplet methyl (*δ*_H_ 1.04), and a methoxy group (*δ*_H_ 3.40). The ^13^C NMR spectrum clearly showed all thirteen carbon atoms and was supported by the HRMS data. Analysis of the HSQC and ^13^C NMR data (Table 6) led to the assignment of two pairs of trisubstituted double bonds (*δ*_C_ 125.2, 126.0, 155.2, and 168.8), one conjugated methine (*δ*_C_ 179.2), four sp^3^ methylenes (*δ*_C_ 22.7, 25.3, 28.8, and 67.0), two sp^3^ methines (*δ*_C_ 42.0, 62.3), a methyl (*δ*_C_ 11.9), and a methoxy (*δ*_C_ 58.9). Since the 1D NMR data of **5**, including carbon types, chemical shifts, and the *J* values, were almost identical to those of (5*R*,6*R*) 6-ethyl-5-hydroxy-3-(methoxymethyl)-5,6,7,8-tetrahydro-4*H*-chromen-4-one (Masahiko et al. 2014), their planar structure and relative configuration were determined to be the same, which was further supported by HMBC (H_3_-11 to C-6/C-10, H_3_-12 to C-9, H-2 to C-9/C-4a/C-8a, H2-9 to C-3/C-5, H-5 to C-3, and H-6 to C-5) and ROESY correlations (H_2_-10/H-5/H-6, and H_3_-11/H-5) (Fig. 8). For compound **5**, the doublet of H-5 with a small *J* value (*J* = 2.2 Hz) was attributed to the *J*ae coupling of H-5 and H-6, as observed in Newman projection analysis (Fig. 8). Hence, the relative configuration of 5 is (5R*,6R*). To determine the absolute configuration of **5**, the ECD trends for two possible configurations, (5*S*,6*S*)-**5** (**5a**) and (5*R*,6*R*)-**5** (**5b**), were calculated in the Gaussian 16 program (Suppl. material 1). Based on the comparison of results, the ECD spectrum of **5a** matched the experimental one more closely than that of **5b** (Fig. 9). Finally, the structure of **5** was defined as (5*S*,6*S*) 6-ethyl-5-hydroxy-3-(methoxymethyl)-5,6,7,8-tetrahydro-4*H*-chromen-4-one and named diplosporin A.

Diplosporin B (**6**) was isolated as a white powder. It shares the same molecular formula (C_13_H_18_O_4_) as that of **5**, based on their HRESIMS spectra. The 1D NMR data of **5** and **6** (Tables 5, 6) were very similar, except for the resonances of CH-5 [**5**: *δ*_C/H_ 62.3/4.83 (d, *J* = 2.2 Hz); **6**: *δ*_C/H_ 69.0/4.59 (d, *J* = 7.7 Hz)]. Further analysis of the 2D NMR spectra of compound **6** (Fig. 8) indicated that it shared the same planar structure as compound **5**, while the NMR differences suggested an opposite configuration of C-5. Unlike compound **5**, the large coupling constant (*J* = 7.7 Hz) of H-5 in 6 was attributable to a *J*_aa_ coupling of H-5/H-6, based on Newman projection analysis (Fig. 8). Meanwhile, the ROESY correlations [H-5/H_2_-10, H-5/H-7*β* (*δ*_H_ 1.45), H-6/H-7*α* (*δ*_H_ 2.03)] showed that H-5 and H-6 were located on opposite sides. In other words, the relative configurations of C-5 and C-6 in **6** were 5R* and 6S*. To identify the absolute configuration of **6**, the ECD spectra for the two possible configurations, (5*R*,6*S*)-**6** (**6a**) and (5*S*,6*R*)-**6** (**6b**), were calculated. As shown in Fig. 9, the ECD spectrum of **6b** was more comparable to the experimental one than that of **6a**. Finally, the structure of **6** was defined as (5*S*,6*R*)-6-ethyl-5-hydroxy-3-(methoxymethyl)-5,6,7,8-tetrahydro-4*H*-chromen-4-one.

**Figure 10.**
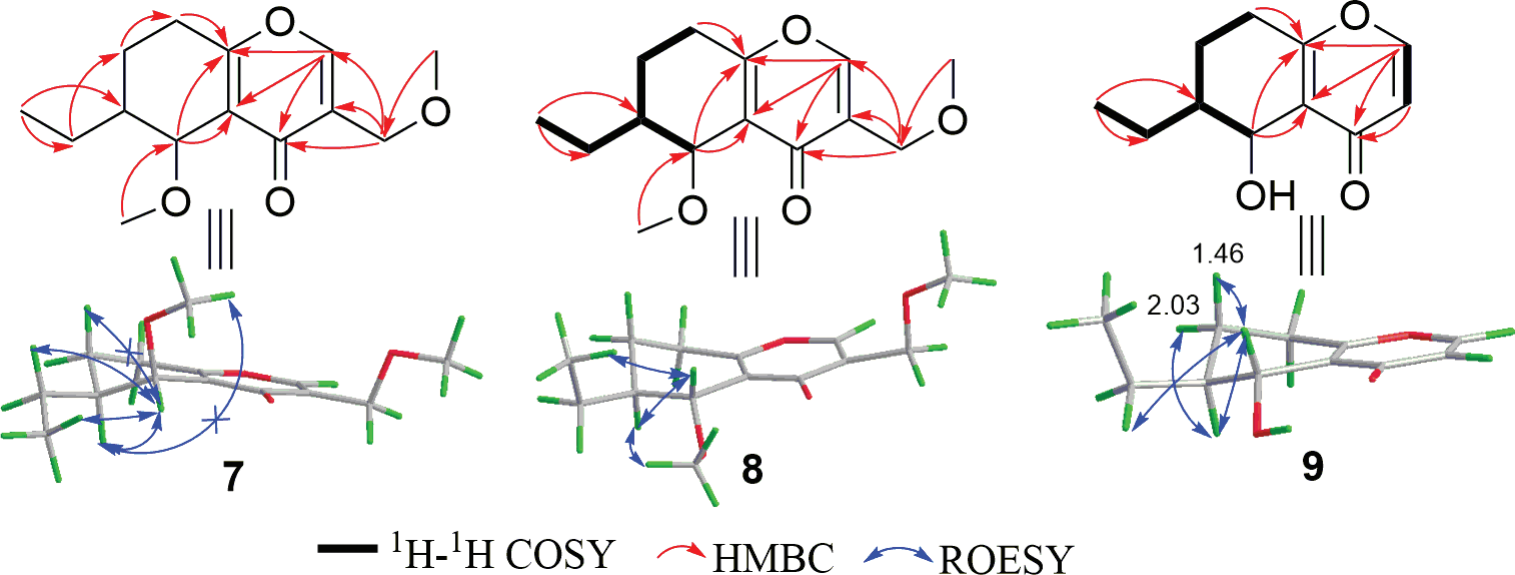
Key 2D NMR correlations of **7**–**9**.

Diplosporin C (**7**) was isolated as a yellow powder. Its molecular formula is C_14_H_20_O_4_ by HRESIMS analysis, bearing 14 mass units (CH_2_) more than **5**. Moreover, the general features of its NMR data (Tables 5, 6) closely resembled those of **5**, indicating that they had analogous structures. Further analysis of the NMR data between compounds **5** and **7** revealed that the main difference was the presence of an additional methoxy (*δ*_C/H_ 60.2/3.54) for **7**, which was substituted at C-5 based on the HMBC correlation from H_3_-13 to C-5 (Fig. 10). The relative configuration for **7** was determined by the JBCA of H-5 and ROESY analysis. The key ROESY correlations of H-6/H-5/H_2_-9 and a small coupling constant (1.7 Hz) suggested that the relative configuration was the same as **5**. Finally, the absolute configuration of compound **7** was confirmed as (5*S*,6*S*) by comparing its experimental CD curve with that of compound **5** (Fig. 9). Therefore, the structure of **7** was defined as (5*S*,6*S*) 6-ethyl-5-methoxy-3-(methoxymethyl)-5,6,7,8-tetrahydro-4*H*-chromen-4-one.

Diplosporin D (**8**) had the same molecular formula as **7**. Furthermore, compounds **7** and **8** exhibited similar signal patterns in the 1D NMR spectra (Tables 5, 6). The primary differences between **7** and **8** were the signals for CH-5 [**7**: *δ*_C/H_ 70.8/4.51 (d, *J* = 1.7 Hz); **8**: *δ*_C/H_ 73.4/4.27 (d, *J* = 7.6 Hz)] and CH-6 [**7**: *δ*_C/H_ 41.2/1.32 (m); **8**: δ_C/H_ 36.4/1.97 (m)], suggesting that they shared the same planar structure but had different relative configurations. Additionally, their CD trends were different (Fig. 9). These signals indicated that the absolute configuration of **8** was (5*S*,6*R*). Therefore, the structure of **8** was confirmed as (5*S*,6*R*) 6-ethyl-5-methoxy-3-(methoxymethyl)-5,6,7,8-tetrahydro-4*H*-chromen-4-one.

The molecular formula of diplosporin E (**9**) is C_11_H_14_O_3_ according to the HRESIMS result. The ^1^H NMR and ^13^C NMR data (Tables 5, 6) of **9** were similar to those of **6**, except for the presence of an additional olefinic methine [*δ*_C/H_116.5/6.30 (d, *J* = 5.7 Hz)] and the absence of 3-methoxymethyl, indicating that the methoxymethyl at C-3 in **6** was replaced by a proton in **9**. This modification was the reason for the doublet signal of H-1 [*δ*_H_ 7.69 (d, *J* = 5.7 Hz)]. Furthermore, ^1^H-^1^H COSY and HMBC correlations (Fig. 10) confirmed the planar structure as 6-ethyl-5-hydroxy-5,6,7,8-tetrahydro-4*H*-chromen-4-one. Moreover, the coupling constant of H-5 (*J* = 7.7 Hz), chemical shifts of related groups (C-4a, CH-5/6, CH_2_-7) (Tables 5, 6), ROESY correlations (Fig. 10), and the CD spectrum for **9** (Fig. 9) closely resembled those of **6**, implying that the absolute configuration of **9** is also (5*S*,6*R*).

**Figure 11.**
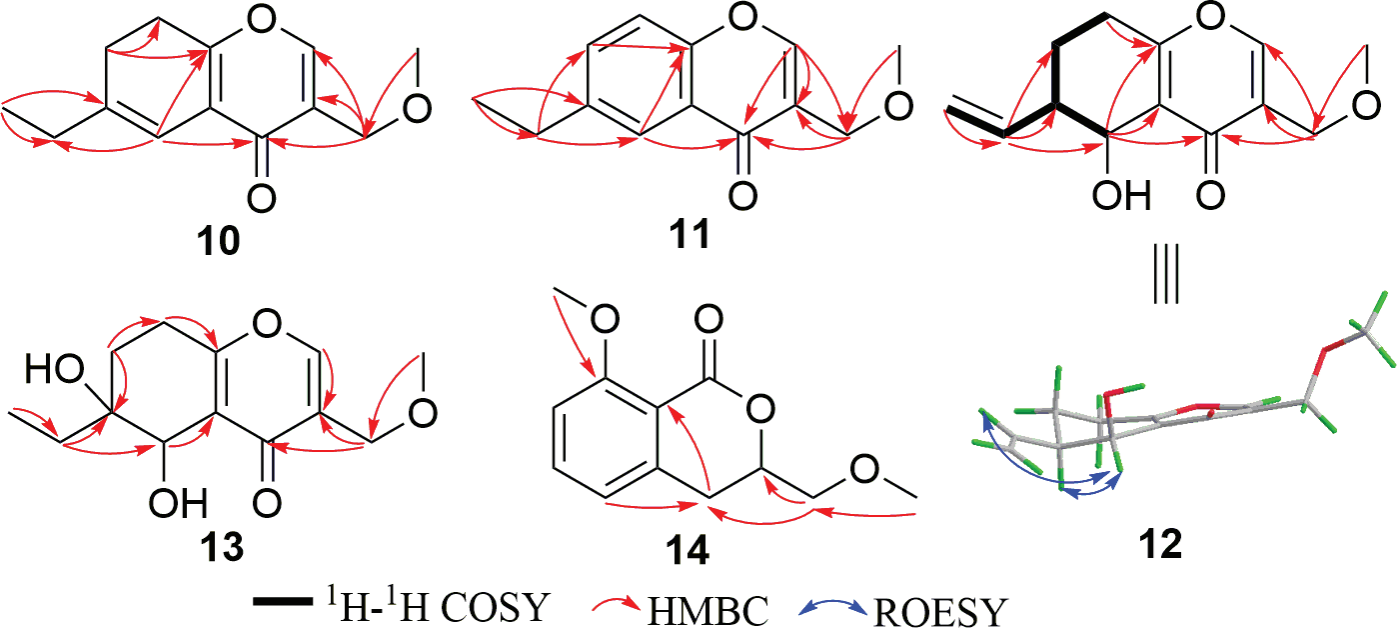
Key 2D NMR correlations of **10**–**14**.

Diplosporin F (**10**) was isolated as yellow oils. Its molecular formula is C_13_H_16_O_3_, indicating six degrees of unsaturation. The 1D NMR data also displayed typical signals for a 6-ethyl-3-(methoxymethyl)-4*H*-chromen-4-one, similar to compounds **5** and **6**. This structural framework accounted for five of the six degrees of unsaturation (Tables 5–7), while the remaining degree of unsaturation is attributed to the presence of a trisubstituted double bond [*δ*_C/H_ 111.4/6.37 (s); 140.4]. A detailed analysis of HMBC correlations from H_3_-11 to C-6 and H-5 to C-4/C-8a effectively assigned the double bond between C-5 and C-6 (Fig. 11). Therefore, the structure of **10** is 6-ethyl-3-(methoxymethyl)-7,8-dihydro-4*H*-chromen-4-one.

Diplosporin G (**11**) was obtained as a yellow powder. The molecular formula of compound **11**, C_13_H_14_O_3_, contains two mass units (H2) less than that of **10**. The 1D NMR data of **11** were quite similar to those of **10**, except for the emergence of a disubstituted double bond [*δ*_C/H_ 134.0/7.51 (dd, *J* = 8.6, 2.2 Hz); 118.1/7.38 (d, *J* = 8.6 Hz)] and the disappearance of two methines in **11** (Tables 6, 7). Additionally, HMBC correlations from H_2_-10 to C-5/C-7 indicated that this double bond was located at C7(8) (Fig. 11). This double bond, along with H-5 [*δ*_H_ 8.03 (d, *J* = 2.2 Hz)], formed an ABX aromatic coupling system. Therefore, the structure of **11** is 6-ethyl-3-(methoxymethyl)-4*H*-chromen-4-one.

The molecular formula of diplosporin H (**12**) is C_13_H_16_O_4_, with six degrees of unsaturation. The similar characteristics of 1D NMR data of **5** and **12** indicate they share the same scaffold. Their 1D NMR data also showed similarity to those of compound **5** (Tables 5–7). In the 1D NMR data, the signals for a terminal olefine [*δ*_C/H_ 138.2/6.08 (m); 116.1/5.22 (dd, *J* = 1.3, 1.3 Hz), 5.19 (dd, *J* = 1.3, 1.3 Hz)] were observed instead of signals for the methyl triplet at C-11 in **12**, implying that a terminal olefine substituted the ethyl at C-6. The HMBC correlations from H-10 to C-5/C-6/C-7 and the ^1^H-^1^H COSY correlations of H_2_-11/H-10/H-6/H-5 supported the above deduction (Fig. 11). The similarities in the *J* value of H-5 (brs) indicated that H-5 and H-6 are co-facial (Table 7). Subsequent calculations of ECD and ^13^C NMR for (5*S*,6*R*)-**12** were performed. The calculated results of (5*S*,6*R*)-**12** were then compared with the experimental results for **12** (Suppl. material 1: figs S96, S97) and matched quite well. Therefore, the absolute structure of **12** was determined to be (5*S*,6*R*)-5-hydroxy-3-(methoxymethyl)-6-vinyl-5,6,7,8-tetrahydro-4*H*-chromen-4-one.

The molecular formula of diplosporin I (**13**) was determined to be C_13_H_18_O_5_ through HRESIMS analysis, which consists of an additional oxygen atom compared to that of **6**. Additionally, the 1D NMR data (Tables 5–7) showed close similarity to those of **6**, implying that **13** is a congener of **11**. The only difference between **6** and **13** is that H-6 in **6** is replaced by a hydroxy in compound **13**. Analysis of the HMBC spectrum of compound **13** enabled the above assignment (Fig. 11). Since no useful ROESY correlations were observed to confirm the relative configuration of compound **13**, calculated ^13^C NMR and ECD spectra of compound **13** were performed. There are two chiral carbons in compound **13**, so it has two pairs of possible enantiomers, (5S*, 6S*)-**13** and (5S*, 6R*)-**13**. Firstly, the ^13^C NMR data of (5S*, 6S*)- and (5S*, 6R*)-**13** were calculated using GIAO B3LYP/6-31G(d) level in Gaussian 16 and then compared with the experimental data of compound **13**. As shown in Suppl. material 1: fig. S99, the *R*^2^, MAE, and Δ_max_ of the (5S*, 6R*)-**13** isomer [*R*^2^ = 0.9992, MAE = 1.6, Δ_max_ = 2.8 ppm (C-12)] were better than those for the (5S*, 6S*)-**13** isomer [*R*^2^ = 0.9978, MAE = 2.6, Δ_max_ = –7.1 ppm (C-5)] (Suppl. material 1), indicating the relative configuration of **13** to be (5S*,6R*). Secondly, the absolute configuration of compound **13** was determined by comparing the calculated ECD with the experimental CD, showing that (5*S*, 6*R*)-**13** exhibited a similar tendency to that shown in Suppl. material 1: fig. S100. Finally, the structure of compound **13** was determined to be (5*S*,6*R*)-6-ethyl-5,6-dihydroxy-3-(methoxymethyl)-5,6,7,8-tetrahydro-4*H*-chromen-4-one.

The molecular formula of compound **14** was determined to be C_12_H_14_O_4_ by HRESIMS, suggesting six degrees of unsaturation. The ^1^H NMR spectrum (Table 8) displayed distinguished signals for two methoxy (*δ*_H_ 3.44 and 3.94) and a set of ABB’ coupling benzene system [*δ*_H_ 6.82 (d, *J* = 8.0 Hz); 7.46 (dd, *J* = 8.0, 8.0 Hz); 6.92 (d, *J* = 8.0 Hz)]. The ^13^C NMR spectrum of **14** revealed twelve carbons, which can be assigned to two methoxys (*δ*_C_ 56.3, 59.7), one sp^3^ methylene (*δ*_C_ 31.5), one oxygenated methylene (*δ*_C_ 73.6), one oxygenated methine (*δ*_C_ 76.3), six aromatic signals (*δ*_C_ 111.1, 119.5, 134.7, 114.8, 141.7, 161.4), and one ester carbonyl (*δ*_C_ 162.1) (Table 8). The NMR data of **14** resemble those of the known compound **22** (Klaiklay et al. 2012), indicating that compound **14** is also an isocoumarin (Fig. 1). Further analysis of the NMR data and mass spectrometry results between **14** and **22** showed that CH_3_-9 in **22** was oxygenated to a methoxymethyl in compound **14**, which was supported by HMBC correlation from CH_3_-11 to C-9 (Fig. 11). To determine the absolute configuration of the chiral carbon C-3, the optical rotation of the one possible configuration of (3*R*)-**14** was calculated. The calculated optical rotation of (3*R*)-**14** ([α] 13.54) and the experimental one ([α] 96.2) are positive values, suggesting that the absolute configuration of C-3 is *R* (Suppl. material 1). Therefore, the structure of **14** is (3*R*)-8-methoxy-3-(methoxymethyl)isochroman-1-one.

Besides, 27 known compounds were obtained from three fermentations of rice (**15**–**29**), potato-dextrose-broth (PDB) (**30**–**34**), and glucose-peptone-yeast (GPY) (**24**, **28**, **35**–**41**). Their structures were identified by comparing 1D NMR, MS, and optical data with the reported ones in the literature. As the optical value of compound **26** was close to zero, it was inferred that this compound is a pair of racemates. They were identified as 16-hydroxyisopimar-7-en-19-oic acid (**15**) (Wu et al. 2011), 16-*α*-*D*-mannopyranosyloxyisopimar-7-en-19-oic acid (**16**) (Zhou et al. 2021; Shiono et al. 2009), 16-*α*-*D*-glucopyranosyloxyisopimar-7-en-19-oic acid (**17**) (Zhou et al. 2021; Shiono et al. 2009), cyclo(N-methyl-L-Phe-L-Val-D-Ile-L-Leu-L-Pro) (**18**) (Wu et al. 2011), 5-hydroxy-6-methoxy-2-methyl-4*H*-1-benzopyran-4-one (**19**) (Sun et al. 2012), 5-methoxy-2-methyl-4*H*-1-benzopyran-4-one (**20**) (Teles et al. 2005), 5-hydroxy-8-methoxy-2-methyl-4*H*-1-benzopyran-4-one (**21**) (Sun et al. 2012), (*S*)-8-methoxy-3-methylisochroman-1-one (**22**) [$[\alpha {]}_{D}^{23}$ +211.3 (*c* 0.23, MeOH)] (Klaiklay et al. 2012), (*R*)-(−)-5-methoxycarbonylmellein (**23**) [$[\alpha {]}_{D}^{23}$ +118.4 (*c* 0.07, MeOH)] (Gonzalez-de-Castro et al. 2014), (*S*)-5-hydroxy-8-methoxy-3-methylisochroman-1-one (**24**) [$[\alpha {]}_{D}^{23}$ +126.2 (*c* 0.02, MeOH)] (Harwood 1983), (*R*)-(−)-5-methoxycarbonylmellein (**25**) [$[\alpha {]}_{D}^{23}$ –113.4 (*c* 0.03, MeOH)] (Holker et al. 1981), (±) 8-hydroxy-4-methylisochroman-1-one (**26**) (Sumarah et al. 2008), 5-methoxy-1-naphthnol (**27**) (Si et al. 2011), 1*H*-indole-3-carboxylic acid methyl ester (**28**) (Bao et al. 2007), 22,23-dihydroxyergosta-4,6,8(14)-trien-3-one (**29**) (Lee et al. 2011), (*R*)-5-carboxymellein (**30**) (Xu et al. 2020; Rukachaisirikul et al. 2009), (22*E*,24*R*)-ergosta-7,22-diene-3*β*,5*α*,6*β*-triol (**31**) (Hata et al. 2002), (3*β*,5*α*,6*β*,22*E*)-6-methoxyergosta-7,22-diene-3,5-diol (**32**) (Shimizu et al. 2016), (24*S*)-ergosta-7-ene-3*β*,5*α*,6*β*-triol (**33**) (Gao et al. 2001), 3*β*,5*α*,9*α*-trihydroxy-(22*E*,24*R*)-ergosta-7,22-dien-6-one (**34**) (Wang et al. 2017), 3-methoxy-2-phenethyl-1*H*-pyrrole-1-carbaldehyde (**35**) (Zhou et al. 2018), tryptoline (**36**) (Lu et al. 2010; Airaksinen et al. 1981), cyclo-((*S*)-Pro-(*R*)-Leu)) (**37**) (Yang et al. 2009), 1-phenylethyl-*O*-*β*-*L*-rhamnopyranoside (**38**) (Wang et al. 2014), 1-phenylethyl-*O*-*α*-*L*-rhamnopyranoside (**39**) (Chapla et al. 2018), 2-hexylidene-3-methylsuccinic acid (**40**) (Chinworrungsee et al. 2001), and 22,23-dihydroxy-ergosta-4,6,8(14)-trien-3-one-23-*β*-D-glucopyranoside (**41**) (Li et al. 2017).

The metabolites produced by *X.longipes* under three different fermentation conditions exhibit significant variation. The common compounds shared across these conditions are phenylpropanoids and ergosteroids. Phenylpropanoids, a major component of essential oils, are produced by many fungi (Chen et al. 2024). Notably, the addition of 3-methyl-2-butenol to PDB fermentation significantly increased the yield of (3*R*)-5-carboxymellein (**30**) to 8.93 mg/L. Ergosteroids, integral components of the fungal cell wall, are consistently co-isolated with other metabolites.

Compared to liquid fermentations, rice fermentation yielded a more diverse array of metabolites, including diterpenoids, cyclic pentapeptides, 2,5-diene-4-pyrane compounds (diplosporins), phenylpropanoids, and ergosteroids. Furthermore, the metabolites from the two liquid fermentations also differed significantly. This suggests that varying fermentation conditions can induce distinct gene expressions, leading to the production of different metabolites.

Previous studies on *X.longipes*GPY cultures identified a series of nor-isopimarane diterpenoids (Chen et al. 2020a, 2020b). However, no terpenoids were detected in the liquid fermentations in this study, likely due to the addition of 5-carboxymellein, which may inhibit terpenoid synthase activity or gene expression.

Pimarane diterpenoids feature a 6-6-6 fused carbocyclic ring system and are classified into four subclasses: pimarane, isopimarane, ent-pimarane, and *ent*-isopimarane. While most pimarane diterpenoids are found in plants such as *Flickingeriafimbriata*, *Chloranthushenryi*, *Basilicumpolystachyon*, and *Sigesbeckiapubescens* (Zhou et al. 2021), only a few have been identified in fungi, including *Xylaria* sp. (Chen et al. 2020a, 2020b; Masahiko et al. 2014; Shiono et al. 2009; Wang et al. 2021; Zhou et al. 2021), *Acremonium* sp., and *Paraconiothyrium* sp. (Ye and Ai 2022; Zhou et al. 2021).

In *Xylaria* sp., pimarane diterpenoids primarily belong to the isopimarane family. The first 11*α*,16-epoxy isopimarane (xylarinorditerpene K) is a norisopimarane from GPY fermentation, while 11*α*,16-epoxy-isopimar-8(9)-en-19*β*-oic acid (**1**) is a rare normal isopimarane diterpenoid featuring an 11-ether substituent. Typically, isopimarane diterpenoids from *Xylaria* sp. are isolated as glycosides with a sugar moiety at C-16 (Shiono et al. 2009; Wang et al. 2021; Zhou et al. 2021). To date, only two sugar moieties have been reported in these compounds: *α*-D-mannose and *α*-D-glucopyranose. Additionally, the double bond in pimarane diterpene glycosides is usually located at C-7(8) (Shiono et al. 2009; Wang et al. 2021; Zhou et al. 2021). However, in the new isopimarane glycosides **2** and **3**, the double bond is positioned at C-8(9).

Macrocyclic peptides from *Xylaria* sp. typically consist of three to six peptides, with cyclic pentapeptides being the most common. So far, fewer than fifteen such peptides have been reported from *Xylaria* sp. (Chen et al. 2024). The isolation of compound **4** contributes to the diversity of known cyclopeptides.

Compared to diterpenoids and macrocyclic cyclic peptides, polyketides are the most abundant class of metabolites from *Xylaria* sp. In this study, nineteen polyketides were isolated, including nine characterized by a 6-ethyl-4*H*-chromen-4-one core (diplosporins) (Ibrahim et al. 2014). Notably, nearly all reported diplosporins, including compounds **5**–**8** and **10**–**13**, feature a 3-methoxymethyl group. In contrast, compound **9** lacks this group due to degradation, making it a novel nordiplosporin.

### ﻿Empirical rules for the configuration of 6-ethyl-5,6,7,8-tetrahydro-4*H*-chromen-4-one

The structural elucidation of natural products continues to pose significant challenges in chemical research. The development of advanced methodologies, including *J*-based configuration analysis (JBCA), nuclear Overhauser effect spectroscopy (NOESY), quantum-chemical calculations, X-ray crystallographic analysis, and the establishment of empirical NMR data rules, has proven effective in determining both relative and absolute configurations of organic molecules. While the planar structures of most small molecules can be reliably determined through NMR spectroscopy coupled with mass spectrometry, the determination of stereochemical configurations remains challenging due to the structural diversity of chiral centers and the difficulties in obtaining suitable single crystals for diffraction analysis. Consequently, identifying characteristic NMR and/or CD patterns for specific compound classes has emerged as a valuable and efficient supplementary approach for absolute configuration determination.

In the present study, a series of diplosporins were isolated from the rice fermentation products of *X.longipes*. While determining their absolute configurations, distinct J-coupling constants and CD spectra patterns were identified and systematically characterized. As illustrated in Figs 8, 9, the coupling constants between H-5 and H-6 (*J*_H-5/H-6_) provided crucial information about their spatial orientation. Specifically, small coupling constants (*J* < 3.0 Hz) indicated identical orientations of H-5 and H-6, while larger values suggested different orientations. Furthermore, the CD spectral patterns exhibited characteristic trends: a “valley-to-peak” sequence indicated *α*-orientation of both H-5 and H-6, whereas the reverse pattern suggested *β*-orientation of H-5 and *α*-orientation of H-6 (Fig. 9).

### ﻿Biological properties

Building on previous reports of immunosuppressive activities of diterpenoids from *X.longipes* against the proliferation of induced T and B lymphocytes, all compounds in this study were evaluated for their anti-proliferative effects on ConA-induced T lymphocytes and LPS-induced B lymphocytes. As summarized in Table 9, compounds **32** and **41** exhibited significant suppressive activity against ConA-induced T lymphocytes, with IC_50_ values of 3.1 and 12.1 *μ*M, respectively. Additionally, compounds **10**, **11**, **32**, **36**, and **41** demonstrated inhibitory effects on LPS-induced B lymphocytes, with IC_50_ values ranging from 8.0 to 20.0 *μ*M. The positive control, dexamethasone (Dex), showed IC_50_ values of 1.9 and 1.0 *μ*M, respectively. Notably, all active compounds displayed CC_50_ values exceeding 20.0 *μ*M, indicating a superior safety profile compared to Dex. In contrast, isopimarane diterpenoids **1**−**3** and **15**−**17** did not exhibit significant suppressive activity against either ConA-induced T lymphocytes or LPS-induced B lymphocytes.

**Table 9.** IC_50_ values of **10**, **11**, **32**, **36**, and **41** against the induced proliferation of induced-T and B lymphocytes, and their CC_50_ values for lymphocytes.

Sample	T cells	B cells	lymphocytes
	IC_50_ (*μ*M)		CC_50_ (*μ*M)
**10**	>20	8.0 ± 0.7	>20
**11**	>20	20.0 ± 1.5	>20
**32**	3.1 ± 0.2	6.0 ± 0.3	>20
**36**	>20	16.7 ± 1.8	>20
**41**	12.1 ± 1.7	16.7 ± 1.9	>20
**Dex**	1.9 ± 0.1	1.0 ± 0.05	0.2 ± 0.04

Given the reported lack of cytotoxicity of nor-isopimarane diterpenoids against T and B lymphocytes at 40 *μ*M (Chen et al. 2020b), the inhibition rates of **1**−**3** and **15**−**17** were further evaluated at two concentrations (40.0 and 20.0 *μ*M). As shown in Table 10, these diterpenoids displayed dose-dependent suppression of cell proliferation in both ConA-induced T lymphocytes and LPS-induced B lymphocytes, with **2** and **15** showing particularly notable activity. Concurrently, high cell viability (Table 10) confirmed that diterpenoids **1**−**3** and **15**−**17** were non-toxic at the cellular level, further supporting their safety profile.

**Table 10.** Inhibition rates of **1**−**3** and **15**−**17** against the cell proliferation of ConA-induced T lymphocytes and LPS-induced B lymphocytes, and cell viability treated with these compounds.

Group	*c* (*μ*M)	Inhibition rate (%)		Cell validity (%)	Group	*c* (*μ*M)	Inhibition rate (%)		Cell validity (%)
		T	B				T	B	
**1**	40	16.5 ± 1.5	20.4 ± 6.3	90.7 ± 4.0	**15**	40	28.8 ± 5.8	29.0 ± 12.2	80.2 ± 1.8
	20	11.4 ± 6.4	18.9 ± 7.5	86.3 ± 2.1		20	26.4 ± 6.3	23.0 ± 4.3	89.9 ± 7.5
**2**	40	32.8 ± 6.8	30.6 ± 6.2	89.8 ± 7.9	**16**	40	20.7 ± 4.3	20.5 ± 10.6	96.2 ± 1.1
	20	23.8 ± 0.9	24.9 ± 8.6	100.0 ± 2.4		20	13.1 ± 2.7	19.3 ± 4.6	101.8 ± 6.9
**3**	40	26.5 ± 3.3	22.6 ± 9.6	89.9 ± 4.4	**17**	40	16.3 ± 4.3	20.7 ± 6.9	100.0 ± 8.5
	20	15.7 ± 7.9	11.0 ± 4.9	87.7 ± 7.4		20	10.1 ± 4.4	11.5 ± 3.1	94.0 ± 5.6
**Dex**	2	45.2 ± 4.9	68.6 ± 1.5	36.9 ± 2.2	Con				100 ± 9.5

**Figure 12.**
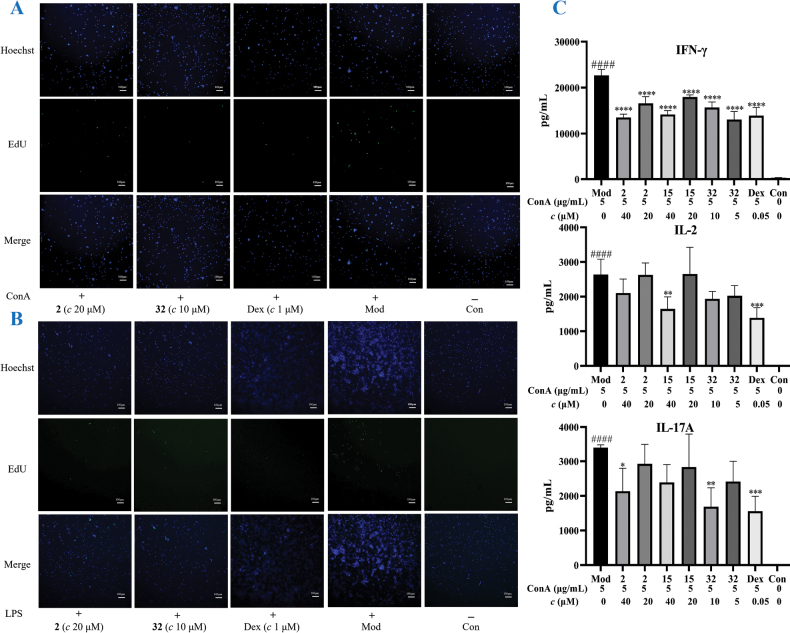
**A, B** Inhibitory activities of **2** (*c* = 20 *μ*M), **32** (*c* = 10 *μ*M), and Dex (*c* = 2, 1 *μ*M) against induced T cells (Con A 5 *μ*g/mL) and B (LPS 15 *μ*g/mL) cells measured by EdU; **C** concentrations of IFN-*γ*, IL-2, and IL-17A of induced T cells (Con A 5 *μ*g/mL) in different groups (*vs.* Con ^####^*P* < 0.0001; *vs.* Mod, ****P < 0.0001, **P < 0.01, *P < 0.05).

Considering the notable immunosuppressive activity of compounds **2** and **32**, which possess distinct structural scaffolds, their inhibitory effects were further investigated using EdU (5-ethynyl-2’-deoxyuridine) imaging kits (HF488). The EdU assay, which detects proliferating cells, revealed that lymphocyte proliferation (indicated by green fluorescence) was markedly enhanced in the model group (Mod) following stimulation with ConA and LPS compared to the control group (Con). In contrast, treatment with compounds **2** and **32** significantly suppressed the proliferation of induced T and B cells relative to the model group (Fig. 12A, B).

Additionally, compounds **2**, **15**, and **32** were evaluated for their ability to inhibit the secretion of cytokines interferon (IFN)-*γ*, interleukin (IL)-2, and IL-17A in mouse splenocytes, following established protocols (Jing et al. 2021; Zhao et al. 2024). As illustrated in Fig. 12C, the levels of IFN-*γ*, IL-2, and IL-17A were significantly elevated in the model group compared to the control group (*P* < 0.0001). Treatment with **2**, **15**, and **32** resulted in a significant reduction in IFN-*γ* secretion across all sample groups (*P* < 0.0001). While these compounds also decreased the secretion of IL-2 and IL-17A, most sample groups did not show statistically significant differences compared to the model group. Notably, compound **15** (40 *μ*M) significantly reduced IL-2 levels (*P* < 0.01), while compounds **2** (40 *μ*M) and **32** (10 *μ*M) markedly lowered IL-17A levels (*P* < 0.05 and *P* < 0.01, respectively). Given the established role of IFN-*γ* in T-cell proliferation (Liu et al. 2022), the antiproliferative effects of **2**, **15**, and **32** on T lymphocytes may be mediated, at least in part, through the suppression of IFN-*γ* secretion.

**Figure 13.**
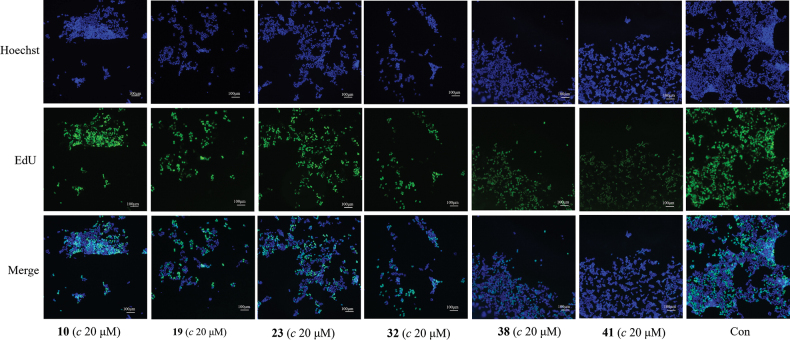
Inhibitory activities of **10**, **19**, **23**, **32**, **38**, and **41** (*c* = 20 *μ*M) against HaCaT cells measured by EdU.

Furthermore, all isolated compounds (**1–41**) were assessed for their antiproliferative activity against the HaCaT cell line, an immortalized keratinocyte model. As summarized in Table 11, compounds **9**, **10**, **13**, **19**, **22**, **24**, **25**, **28**, **30**, **32**–**35**, and **37–41** exhibited inhibitory effects on HaCaT cell proliferation, with IC_50_ values ranging from 25.2 to 35.1 *μ*M. Notably, the IC_50_ value of the positive control, methotrexate (MTX), was 26.4 *μ*M. Complementary EdU experiments (Fig. 13) corroborated these findings, demonstrating that the proliferation rates of HaCaT cells in sample groups treated with compounds **10**, **19**, **23**, **32**, **38**, and **41** were significantly reduced compared to the control group, consistent with the results obtained from the CCK-8 assay.

**Table 11.** IC_50_ values of active compounds against the proliferation of HaCaT cells.

Sample	IC_50_ (*μ*M)	Sample	IC_50_ (*μ*M)
**9**	35.0 ± 3.1	**33**	33.9 ± 2.8
**10**	30.9 ± 2.7	**34**	32.2 ± 3.4
**13**	31.1 ± 2.6	**35**	31.5 ± 2.4
**19**	29.70 ± 3.3	**37**	32.0 ± 2.2
**22**	32.3 ± 1.5	**38**	25.2 ± 1.6
**24**	32.5 ± 2.0	**39**	32.6 ± 3.6
**25**	35.1 ± 1.9	**40**	32.9 ± 3.4
**28**	29.1 ± 1.7	**41**	27.7 ± 2.5
**30**	31.2 ± 4.1	MTX	26.4 ± 1.4
**32**	27.0 ± 1.3		

**Figure 14.**
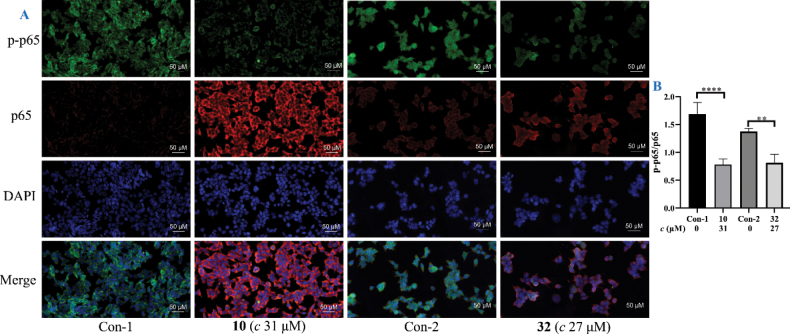
**A** immunofluorescence images: Effects of **10** (*c* = 31 *μ*M) and **32** (*c* = 27 *μ*M) on p-p65 and p65 expression levels in HaCaT cells; **B** ratios of p-p65/p65 of HaCaT cells in different groups (*vs.* Con, **** *P* < 0.0001, **P < 0.01).

Nuclear factor-*κ*B (NF-*κ*B), a family of heterodimeric proteins comprising the p50 and p65 subunits, is critical in regulating cellular proliferation, differentiation, and survival (Hexum et al. 2012). Since compounds **10** and **32** demonstrated inhibitory effects on the proliferation of induced T/B lymphocytes and HaCaT cells, their molecular mechanisms were further investigated concerning the NF-*κ*B pathway. As shown in Fig. 14, compounds **10** (31.0 *μ*M) and **32** (27.0 *μ*M) significantly attenuated NF-*κ*B activation, with *P* values of less than 0.0001 and 0.01, respectively.

Psoriasis, a chronic inflammatory skin disorder affecting 2–3% of the global population, remains without a definitive cure (Dai et al. 2024). The pathophysiology of psoriasis is characterized by the hyperproliferation of keratinocytes, driven by excessive pro-inflammatory cytokines such as IFN-*γ* and IL-17A, which are predominantly secreted by activated T cells, particularly Th17 cells (Castaldo et al. 2021; Rendon and Schäkel 2019). Given that compound **32** effectively suppressed the proliferation of induced T lymphocytes and HaCaT cells and the secretion of key cytokines (IFN-*γ* and IL-17A), it emerges as a promising candidate for further investigation as a potential therapeutic agent for psoriasis.

### ﻿Analysis of the structure-activity relationship

For immunosuppressive activity, compounds **10**, **11**, **32**, **36**, and **41** demonstrated notable efficacy, representing three structural classes: diplosporins (**10**, **11**), an indole alkaloid (**36**), and ergosterols (**32**, **41**). Ergosterols, along with Dex, belong to the steroid family. The inhibitory effects of compounds **32** and **41** on the proliferation of induced T and B lymphocytes can be attributed to their steroid nucleus. Notably, the activities of **32** and **41** surpassed those of their analogs **31** and **29**, respectively, suggesting that the OCH_3_-6 group in **32** and the 23-glucopyranosyl moiety in **41** are critical functional groups for enhancing immunosuppressive activity.

Compounds **10**, **11**, and **36** exhibited significant selectivity in inhibiting LPS-induced B lymphocyte proliferation. Among the diplosporins, the presence of a double bond at C5(6) in compounds **10** and **11** appears to be a key structural feature contributing to their immunosuppressive activity. As shown in Table 9, compound **10** displayed superior activity compared to compound **11**, indicating that higher degrees of unsaturation may negatively impact the immunosuppressive efficacy of these compounds.

## ﻿Conclusion

The *Xylaria* genus, a widely distributed filamentous fungus found in marine and terrestrial environments, is known for producing diverse secondary metabolites. A comprehensive phytochemical investigation of three fermentation cultures of *X.longipes* led to the isolation of 41 metabolites, including fourteen novel compounds (**1**−**14**). These metabolites encompassed a variety of structural classes, such as isopimarane-type diterpenoids, isopimarane-type diterpenoid glycosides, cyclic peptides, polyketides, phenylpropanoids, pyrroles, and ergosterols. Notably, a shift in the production of secondary metabolites was observed when transitioning from solid (rice) to liquid media (PDB and GPY, both supplemented with 3-methyl-2-butenol), highlighting the media-dependent nature of metabolite biosynthesis. This study also validated the utility of combining ^3^*J* coupling constants with CD trends for determining the absolute configurations of C-5 and C-6 in diplosporins.

All isolated compounds were evaluated for their anti-proliferative activity against ConA-induced T lymphocytes, LPS-induced B lymphocytes, and HaCaT cells *in vitro*, with several compounds demonstrating significant activity. Ergosteroids **32** and **41** exhibited potent immunosuppressive and antiproliferative effects against HaCaT cells while maintaining high lymphocyte safety. Cytokine analysis revealed that active compounds **2**, **15**, and **32** significantly reduced IFN-*γ* levels, with compound **32** (10 *μ*M) also inhibiting IL-17A secretion. Further immunofluorescence assays indicated that the antiproliferative effects of compounds **10** and **32** on HaCaT cells were mediated, at least in part, through the inactivation of NF-*κ*B via reduced phosphorylation of p65. Given its multifaceted activities, compound **32** is a promising candidate for developing therapeutic agents for psoriasis.

## ﻿Additional information

### Use of AI

No use of AI was reported.

### Adherence to national and international regulations

All the fungal strains used in this study have been legally obtained, respecting the Convention on Biological Diversity (Rio Convention).

### Author ORCIDs

Zhen-Zhu Zhao https://orcid.org/0000-0001-7961-4694

Zhen-Zhen Wang https://orcid.org/0000-0001-6961-4367

Xin Pang https://orcid.org/0000-0002-8914-219X

### Data availability

All of the data that support the findings of this study are available in the main text or Supplementary Information.

## Supplementary materials

Supplementary material 1 Additional material, including NMR, HRMS data, computational details, and other relevant information on compounds 1–14, together with all the obtained sequences deposited in GenBank Data type pdf https://binary.pensoft.net/file/1393073This dataset is made available under the Open Database License (http://opendatacommons.org/licenses/odbl/1.0/). The Open Database License (ODbL) is a license agreement intended to allow users to freely share, modify, and use this Dataset while maintaining this same freedom for others, provided that the original source and author(s) are credited.Zhen-Zhu Zhao, Yan Wang, Xiao-Yu Wang, Hui Chen, Zhen-Zhen Wang, Jing-Kun Wang, Le-Le Wang, Ming-Jun Shen, Xin Pang, Wei-Sheng Feng
