# Supplementary material for: ﻿Diverse metabolites with anti-psoriasis potential from different fermentations of the fungicolous fungus Xylarialongipes HFG1018
Source: IMA Fungus. 2025 Aug 12;16:e153522. doi: 10.3897/imafungus.16.153522 (PMC12365674; doi:10.3897/imafungus.16.153522)
Supplement: Supplementary material 1 — Additional material, including NMR, HRMS data, computational details, and other relevant information on compounds 1–14, together with all the obtained sequences deposited in GenBank [file imafungus-16-e153522-s001.pdf]

Supplementary Information for

Diverse metabolites with anti-psoriasis potential from different fermentations of the fungicolous fungus *Xylaria longipes* HFG1018

Zhen-Zhu Zhao[1], Yan Wang[1], Xiao-Yu Wang[2], Hui Chen[1], Zhen-Zhen Wang[2], Jing-Kun Wang[1], Le-Le Wang[1], Ming-Jun Shen[1], Xin Pang[1]*, Wei-Sheng Feng[1]*

[1] School of Pharmacy, Henan University of Chinese Medicine, Zhengzhou 450046, China

[2] Academy of Chinese Medical Sciences, Henan University of Chinese Medicine, Zhengzhou 450046, China

Correspondence:

*Xin Pang, Email: pangxin116@163.com
*Wei-Sheng Feng, Email: fwsh@hactcm.edu.cn

Contents

1. Comparative analysis of ITS, *TUB2,* and *RPB2* sequences with available GenBank sequences 5
2. Spectral Figures 6
Fig. S1. ^{1}H NMR spectrum of **1** (500 MHz, $CDCl_3$) 6
Fig. S2. ^{13}C NMR spectrum of **1** (125 MHz, $CDCl_3$) 6
Fig. S3. HSQC spectrum of **1** 7
Fig. S4. ^{1}H-^{1}H COSY spectrum of **1** 7
Fig. S5. HMBC spectrum and partial enlarged HMBC spectrum of **1** 8
Fig. S6. ROESY spectrum of **1** 9
Fig. S7. HRESIMS report of **1** 10
Fig. S8. IR spectrum of **1** 10
Fig. S9. ^{1}H NMR spectrum of **2** (500 MHz, DMSO-d_6) 11
Fig. S10. ^{13}C and DEPT NMR spectra of **2** (125 MHz, DMSO-d_6) 11
Fig. S11. HSQC spectrum of **2** 12
Fig. S12. ^{1}H-^{1}H COSY spectrum of **2** 12
Fig. S13. HMBC spectrum of **2** 13
Fig. S14. ROESY spectrum of **2** 13
Fig. S15. HRESIMS report of **2** 14
Fig. S16. ^{1}H NMR spectrum of **3** (500 MHz, DMSO-d_6) 14
Fig. S17. ^{13}C NMR spectrum of **3** (125 MHz, DMSO-d_6) 15
Fig. S18. HSQC spectrum of **3** 15
Fig. S19. ^{1}H-^{1}H COSY spectrum of **3** 16
Fig. S20. HMBC spectrum of **3** 16
Fig. S21. ROESY spectrum of **3** 17
Fig. S22. HRESIMS report of **3** 17
Fig. S23. ^{1}H NMR spectrum of **4** (500 MHz, DMSO-d_6) 18
Fig. S24. ^{13}C and DEPT NMR spectra of **4** (125 MHz, DMSO-d_6) 18
Fig. S25. HSQC spectrum of **4** 19
Fig. S26. ^{1}H-^{1}H COSY spectrum of **4** 19
Fig. S27. HMBC spectrum of **4** 20
Fig. S28. ROESY spectrum of **4** 20
Fig. S29. HRESIMS report of **4** 21
Fig. S30. ^{1}H NMR spectrum of **5** (500 MHz, CD_3OD) 21
Fig. S31. ^{13}C NMR spectrum of **5** (125 MHz, CD_3OD) 22
Fig. S32. HSQC spectrum of **5** 22
Fig. S33. HMBC spectrum of **5** 23
Fig. S34. ROESY spectrum of **5** 23
Fig. S35. HRESIMS report of **5** 24
Fig. S36. ^{1}H NMR spectrum of **6** (500 MHz, $CDCl_3$) 24
Fig. S37. ^{13}C NMR spectrum of **6** (125 MHz, $CDCl_3$) 25
Fig. S38. HSQC spectrum of **6** 25
Fig. S39. ^{1}H-^{1}H COSY spectrum of **6** 26

Fig. S40. HMBC spectrum of **6** 26
Fig. S41. ROESY spectrum of **6** 27
Fig. S42. HRESIMS report of **6** 27
Fig. S43. ^{1}H NMR spectrum of **7** (500 MHz, $CDCl_3$) 28
Fig. S44. ^{13}C NMR spectrum of **7** (125 MHz, $CDCl_3$) 28
Fig. S45. HSQC spectrum of **7** 29
Fig. S46. HMBC spectrum of **7** 29
Fig. S47. ROESY spectrum of **7** 30
Fig. S48. HRESIMS report of **7** 30
Fig. S49. ^{1}H NMR spectrum of **8** (500 MHz, $CDCl_3$) 31
Fig. S50. ^{13}C NMR spectrum of **8** (125 MHz, $CDCl_3$) 31
Fig. S51. HSQC spectrum of **8** 32
Fig. S52. ^{1}H-^{1}H COSY spectrum of **8** 32
Fig. S53. HMBC spectrum of **8** 33
Fig. S54. ROESY spectrum of **8** 33
Fig. S55. HRESIMS report of **8** 34
Fig. S56. ^{1}H NMR spectrum of **9** (500 MHz, $CDCl_3$) 34
Fig. S57. ^{13}C NMR spectrum of **9** (125 MHz, $CDCl_3$) 35
Fig. S58. HSQC spectrum of **9** 35
Fig. S59. ^{1}H-^{1}H COSY spectrum of **9** 36
Fig. S60. HMBC spectrum of **9** 36
Fig. S61. ROESY spectrum of **9** 37
Fig. S62. HRESIMS report of **9** 37
Fig. S63. ^{1}H NMR spectrum of **10** (500 MHz, $CDCl_3$) 38
Fig. S64. ^{13}C NMR spectrum of **10** (125 MHz, $CDCl_3$) 38
Fig. S65. HSQC spectrum of **10** 39
Fig. S66. HMBC spectrum of **10** 39
Fig. S67. ^{1}H NMR spectrum of **11** (500 MHz, $CDCl_3$) 40
Fig. S68. ^{13}C NMR spectra of **11** (125 MHz, $CDCl_3$) 40
Fig. S69. HSQC spectrum of **11** 41
Fig. S70. HMBC spectrum of **11** 41
Fig. S71. HRESIMS report of **11** 42
Fig. S72. ^{1}H NMR spectrum of **12** (500 MHz, $CDCl_3$) 42
Fig. S73. ^{13}C NMR spectrum of **12** (125 MHz, $CDCl_3$) 43
Fig. S74. HSQC spectrum of **12** 43
Fig. S75. ^{1}H-^{1}H COSY spectrum of **12** 44
Fig. S76. HMBC spectrum of **12** 44
Fig. S77. ROESY spectrum of **12** 45
Fig. S78. HRESIMS report of **12** 45
Fig. S79. ^{1}H NMR spectrum of **13** (500 MHz, $CDCl_3$) 46
Fig. S80. ^{13}C NMR spectrum of **13** (125 MHz, $CDCl_3$) 46
Fig. S81. HSQC spectrum of **13** 47
Fig. S82. HMBC spectrum of **13** 47
Fig. S83. ROESY spectrum of **13** 48

Fig. S84. HRESIMS report of **13**......48
Fig. S85. ^{1}H NMR spectrum of **14** (500 MHz, $CDCl_3$)......49
Fig. S86. ^{13}C NMR spectrum of **14** (125 MHz, $CDCl_3$)......49
Fig. S87. HSQC spectrum of **14**......50
Fig. S88. HMBC spectrum of **14**......50
Fig. S89. HRESIMS report of **14**......51
Fig. S90. Comparison of chemical shifts of pyranoses in compounds **2**, **16**, **3**, and **17**......51
3. Comparison of 1D NMR data between **4** and **18**......52
4. Computational details......53
4.1 Computational details for compound **1**......53
4.2 Computational details for compound **3**......54
4.3 Computational details for compounds **5** and **6**......54
4.4 Computational details for **12**......55
4.5 Computational details for **13**......57
4.6 Computational details for **14**......60
References......60

1. Comparative analysis of ITS, *TUB2,* and *RPB2* sequences with available GenBank sequences

Table S1. Comparative analysis of ITS, *TUB2,* and *RPB2* sequences with the available GenBank sequences.

Sequence	Description	Scientific Name	Per. Ident (%)	Accession
ITS	*Xylaria ellisii* DAOMC 252031 ITS region; from TYPE material	*Xylaria ellisii*	96.59	NR_172972.1
TUB2	*Xylaria longipes* isolate CBS 148.73 beta-tubulin gene, partial cds	*Xylaria longipes*	96.4	KU684204.1
rpb2	*Xylaria longipes* isolate CBS 148.73 RNA polymerase II second largest subunit (RPB2) gene, partial cds	*Xylaria longipes*	97.28	KU684280.1

2. Spectral Figures

Fig. S1. ^{1}H NMR spectrum of 1 (500 MHz, $CDCl_3$)

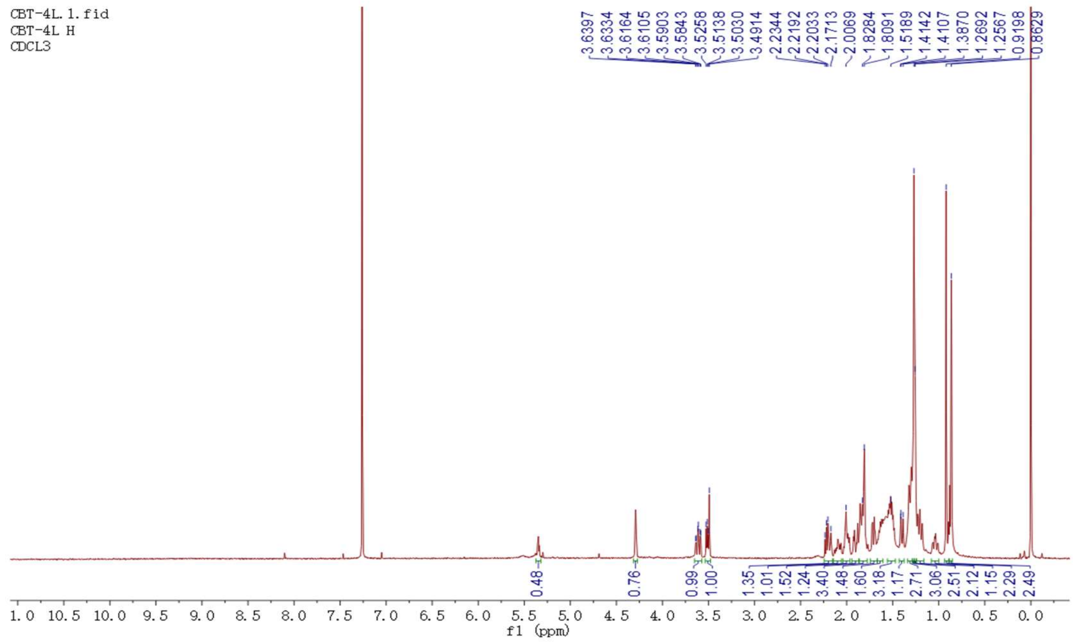

Fig. S2. ^{13}C NMR spectrum of 1 (125 MHz, $CDCl_3$)

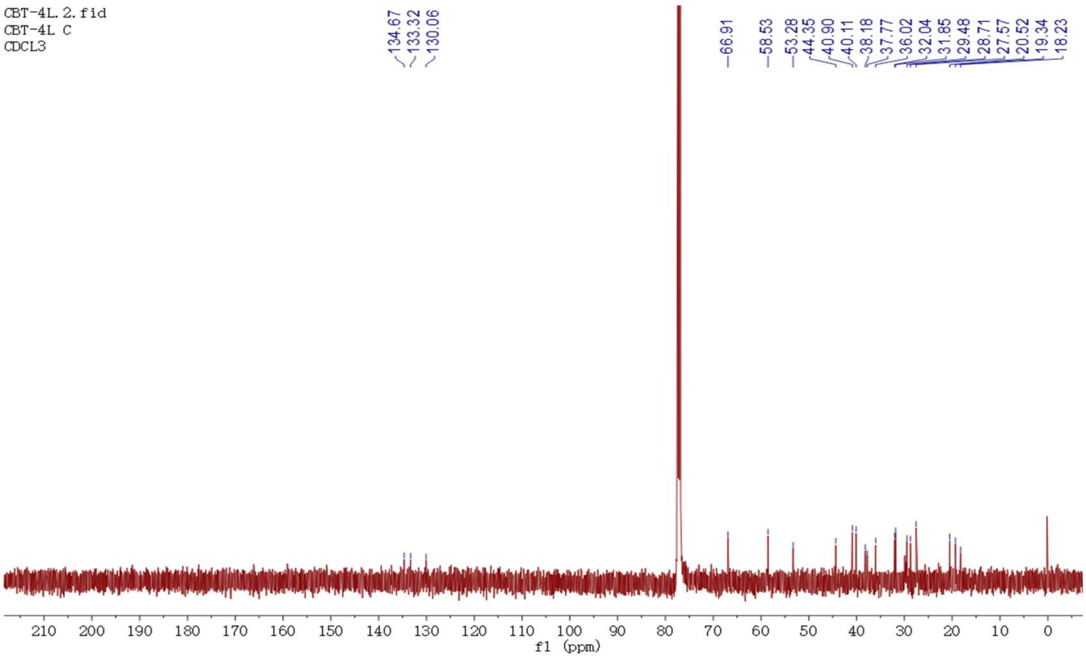

Fig. S3. HSQC spectrum of 1

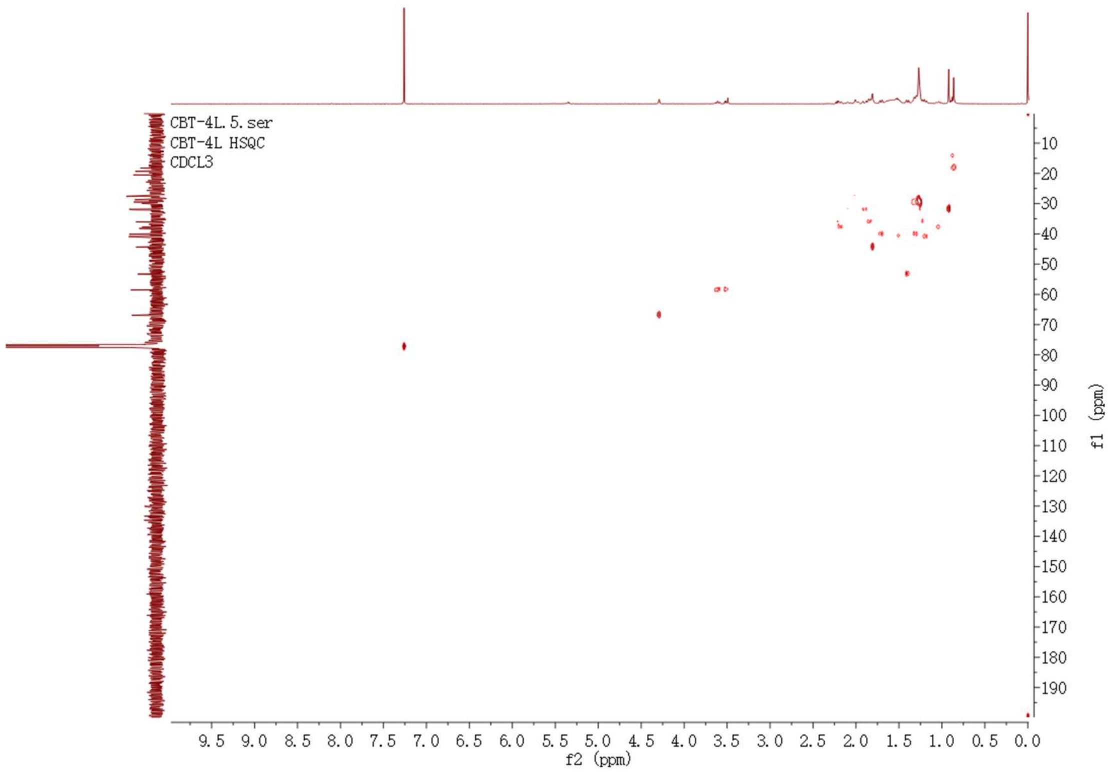

Fig. S4. ¹H-¹H COSY spectrum of 1

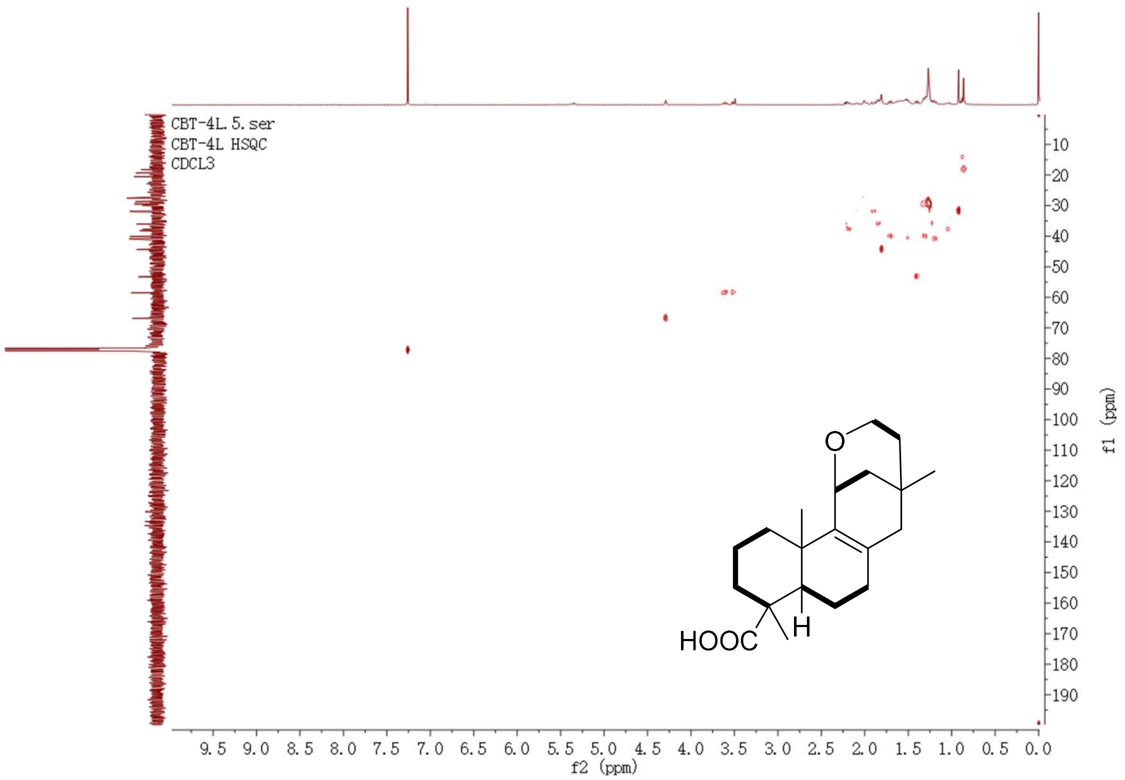

Fig. S5. HMBC spectrum and partial enlarged HMBC spectrum of 1

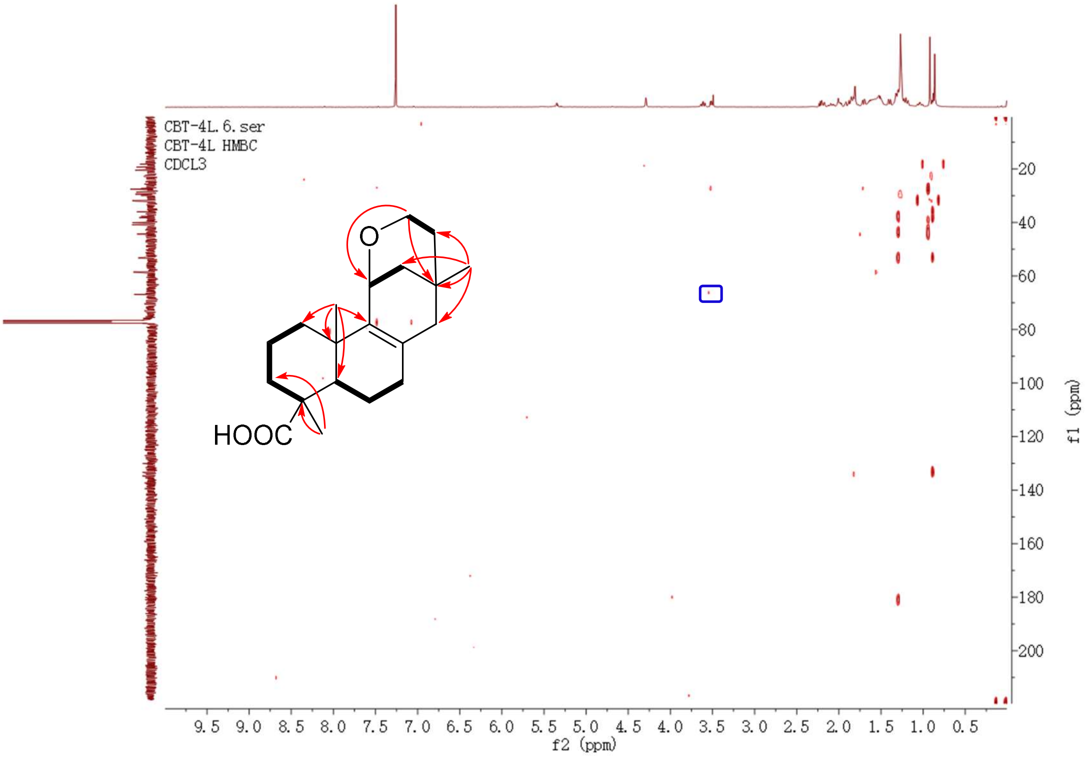

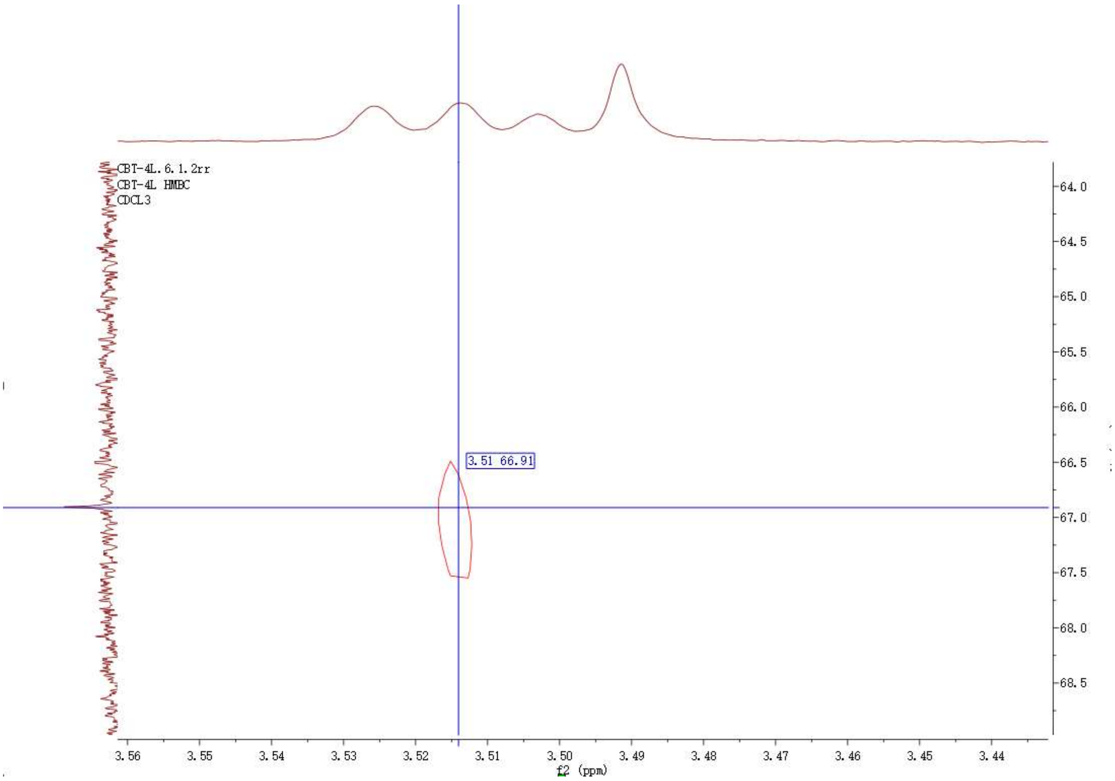

Fig. S6. ROESY spectrum of 1

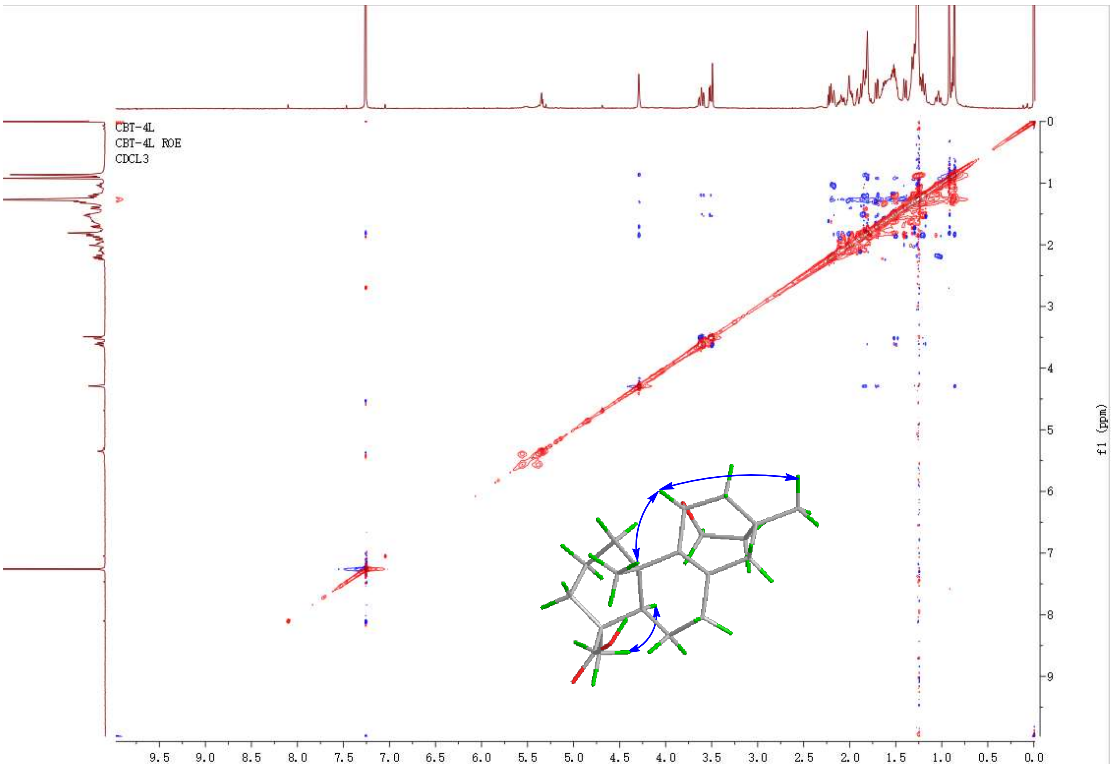

Fig. S7. HRESIMS report of 1

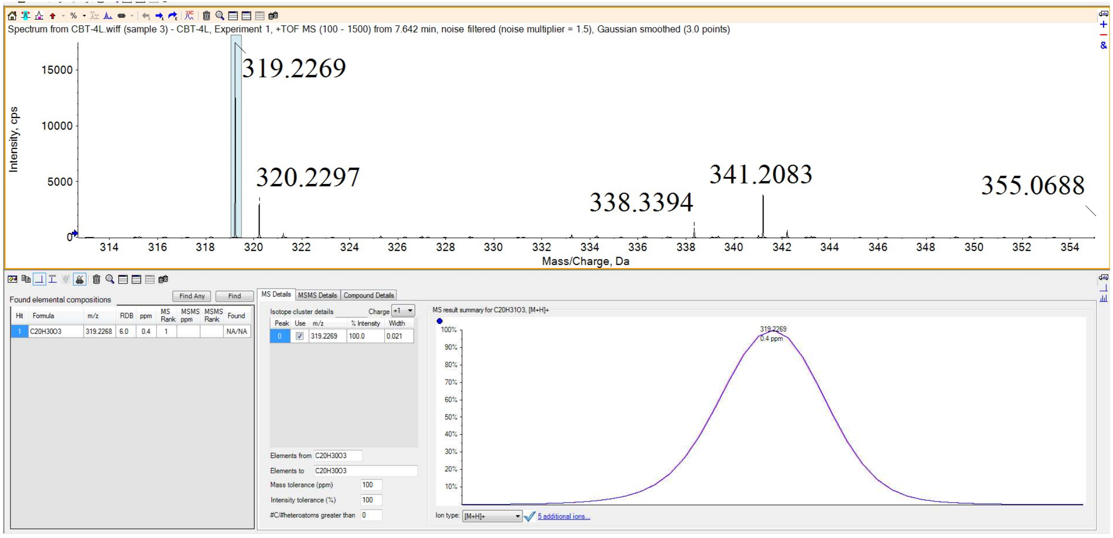

Fig. S8. IR spectrum of 1

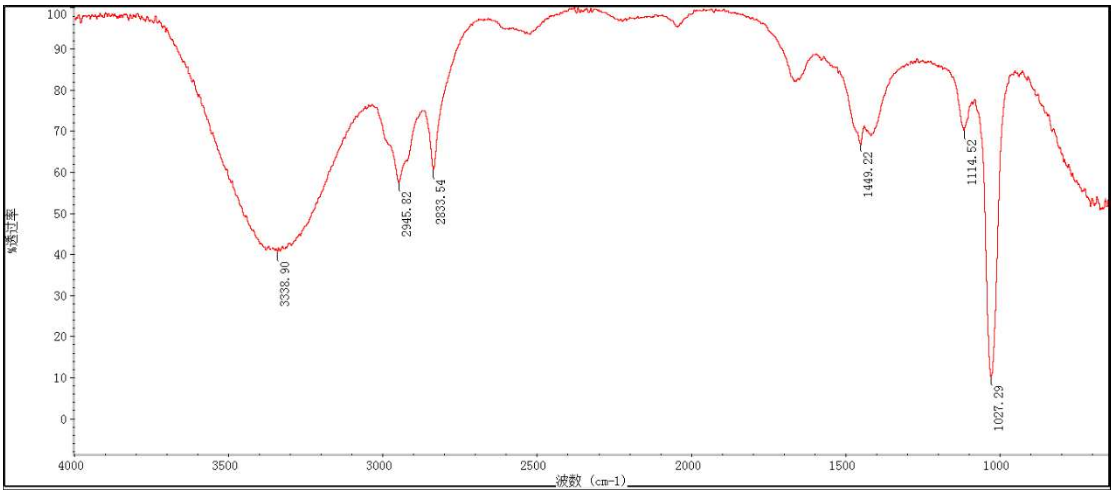

Fig. S9. ^{1}H NMR spectrum of 2 (500 MHz, DMSO-d_6)

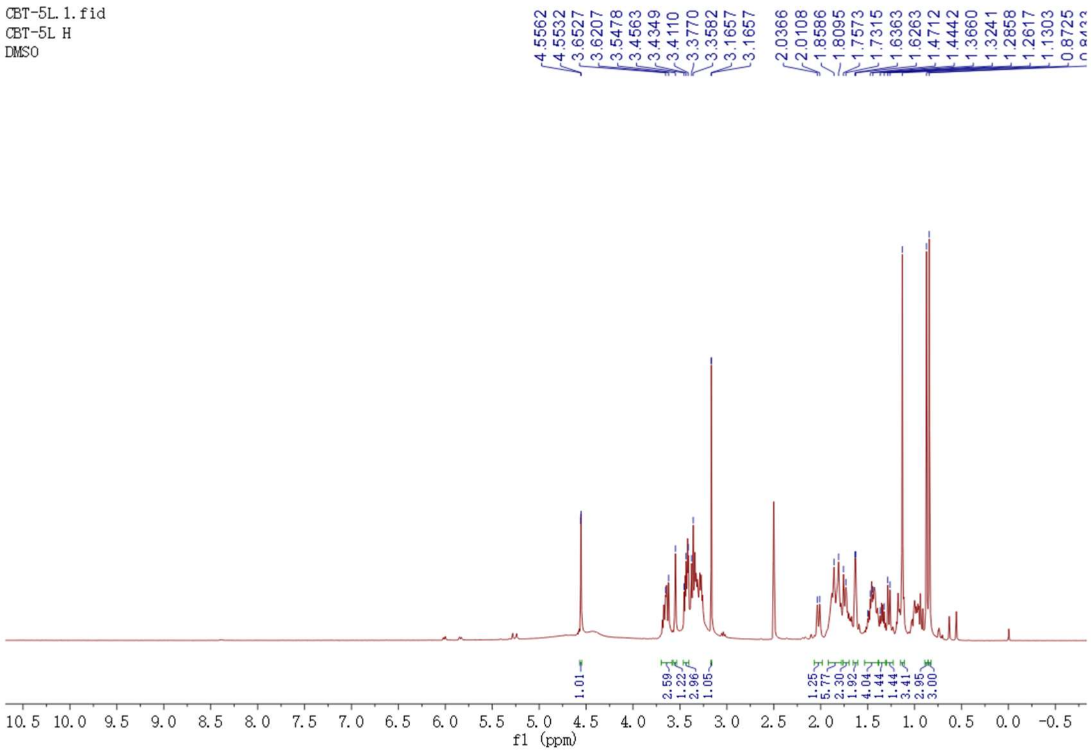

Fig. S10. ^{13}C and DEPT NMR spectra of 2 (125 MHz, DMSO-d_6)

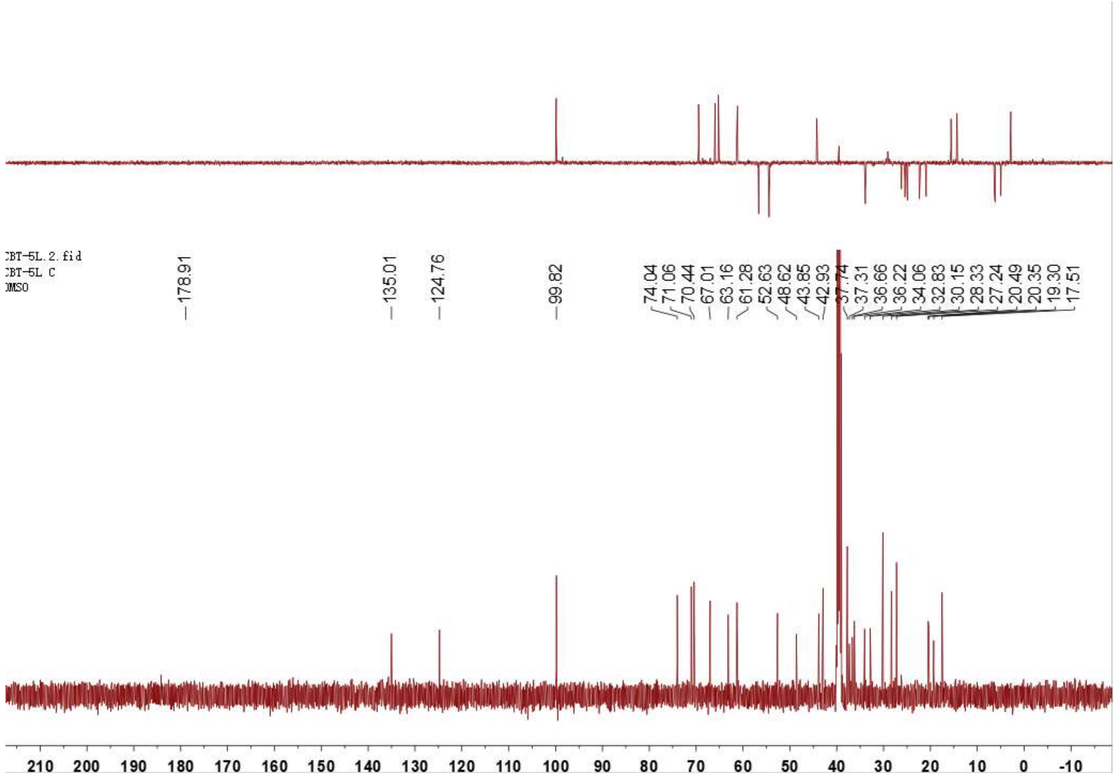

Fig. S11. HSQC spectrum of 2

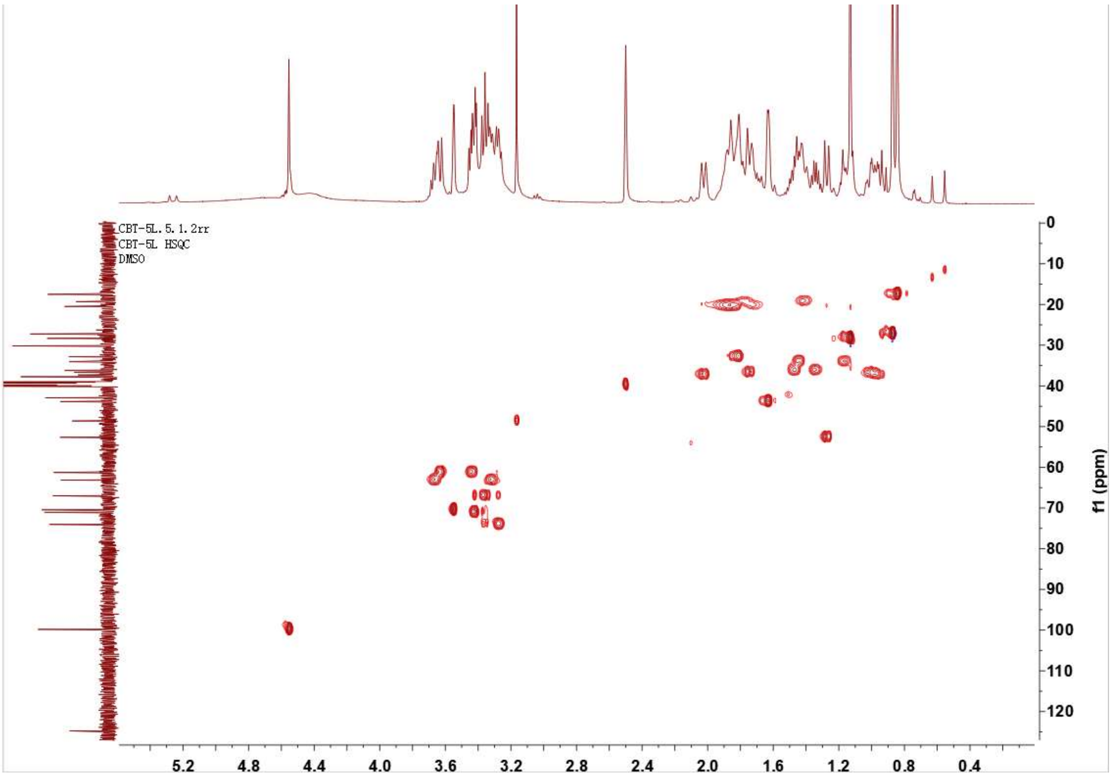

Fig. S12. ^{1}H-^{1}H COSY spectrum of 2

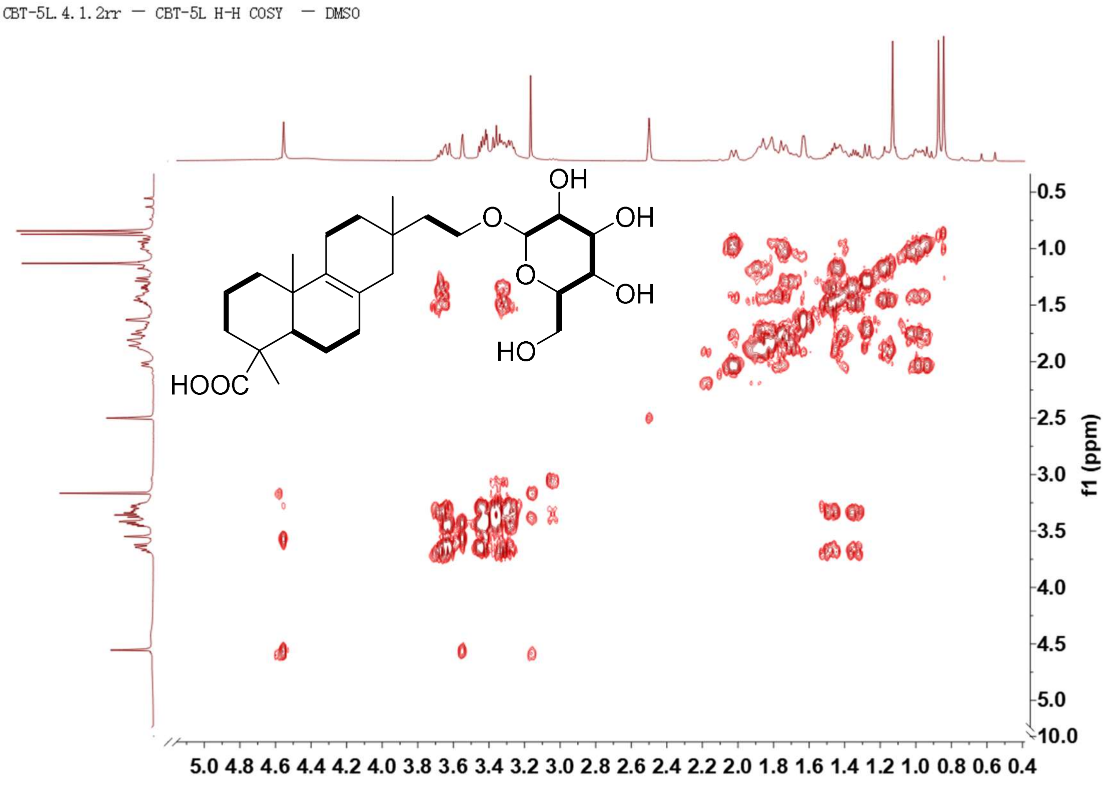

Fig. S13. HMBC spectrum of 2

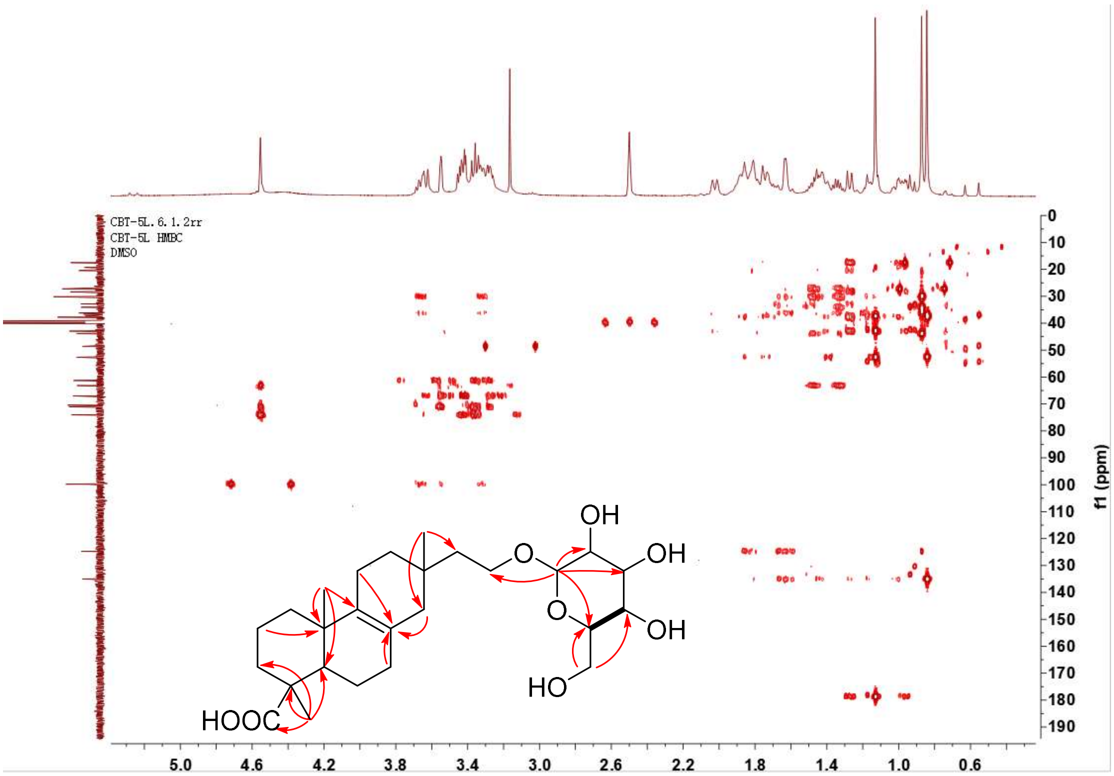

Fig. S14. ROESY spectrum of 2

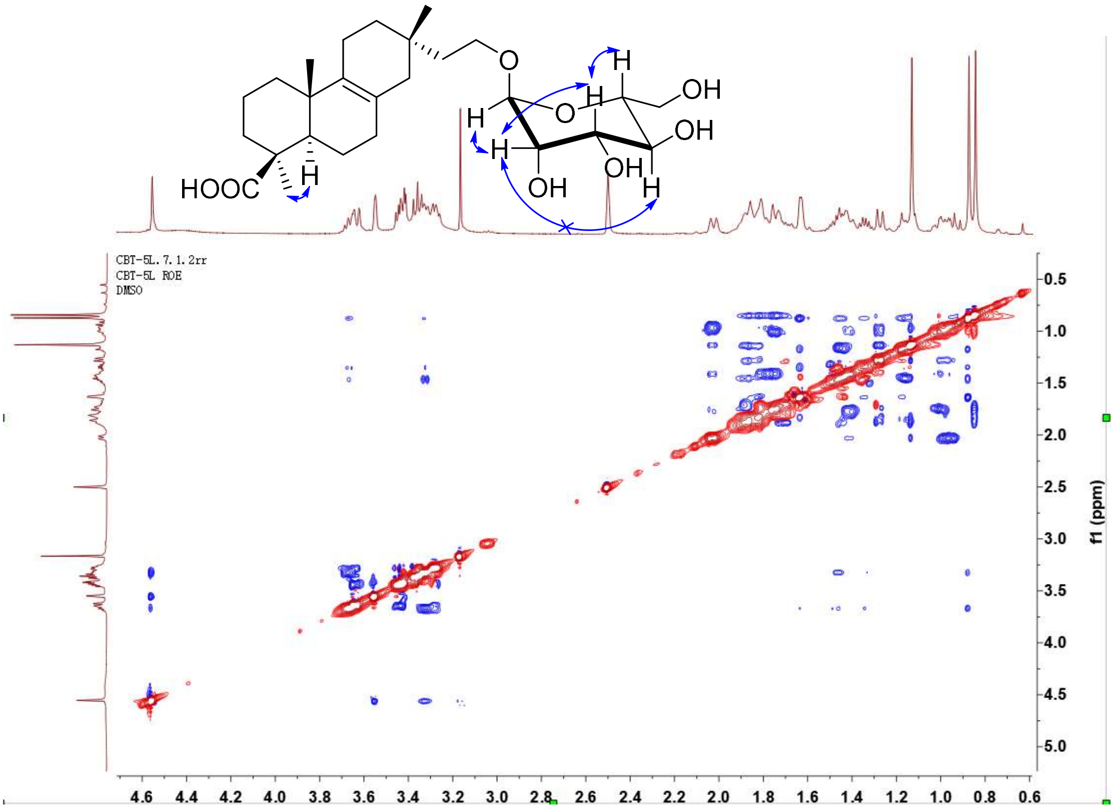

Fig. S15. HRESIMS report of 2

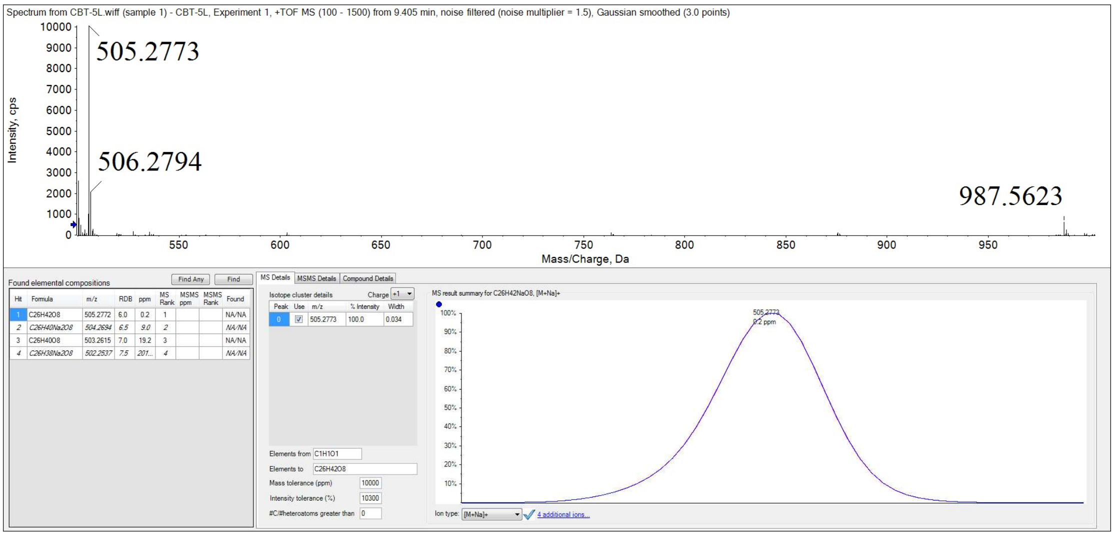

Fig. S16. ^{1}H NMR spectrum of 3 (500 MHz, DMSO-d_6)

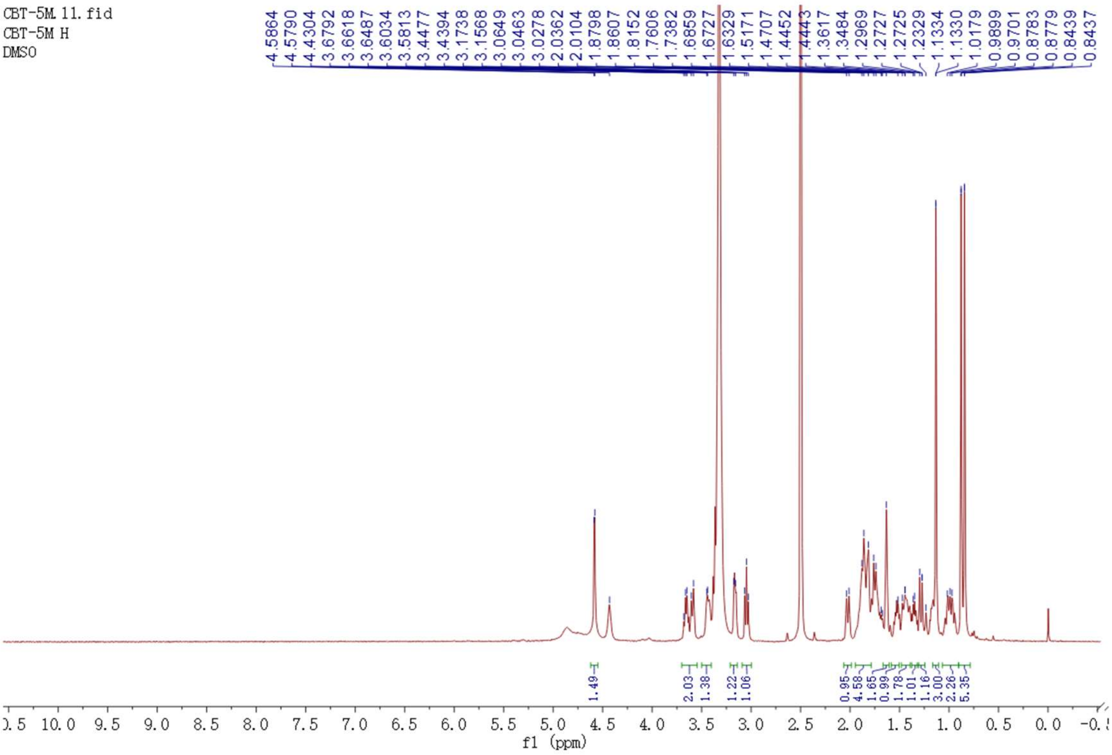

Fig. S17. ^{13}C NMR spectrum of 3 (125 MHz, DMSO-d_6)

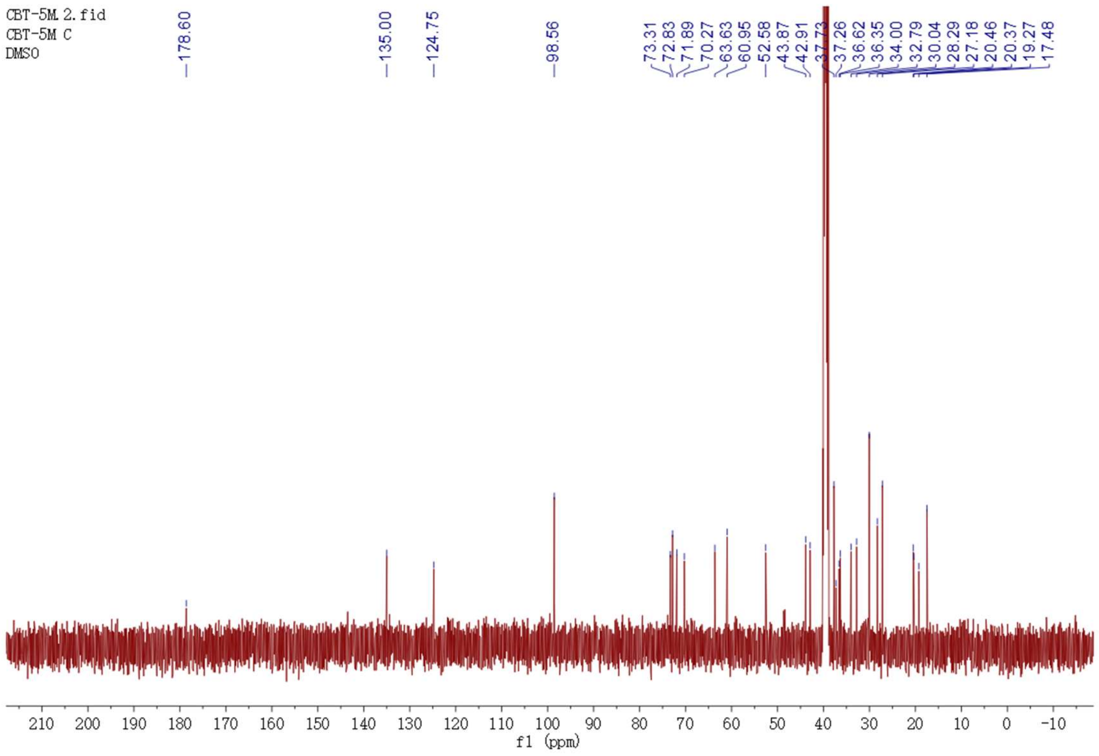

Fig. S18. HSQC spectrum of 3

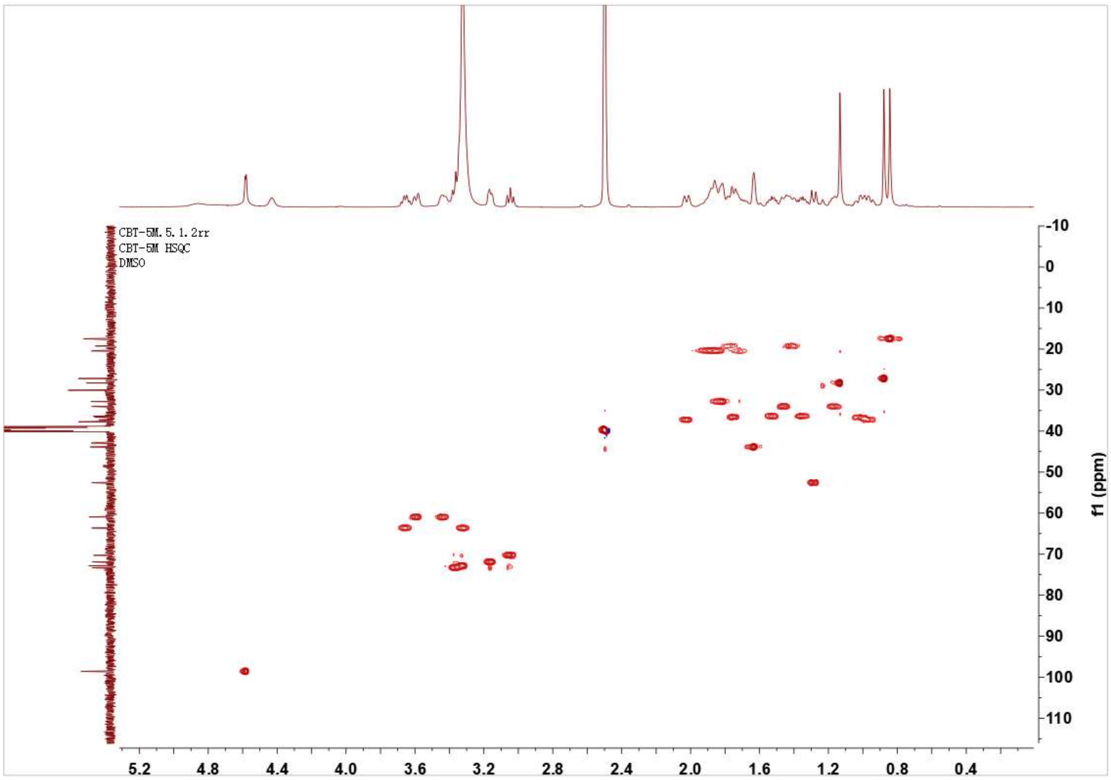

Fig. S19. ^{1}H-^{1}H COSY spectrum of 3

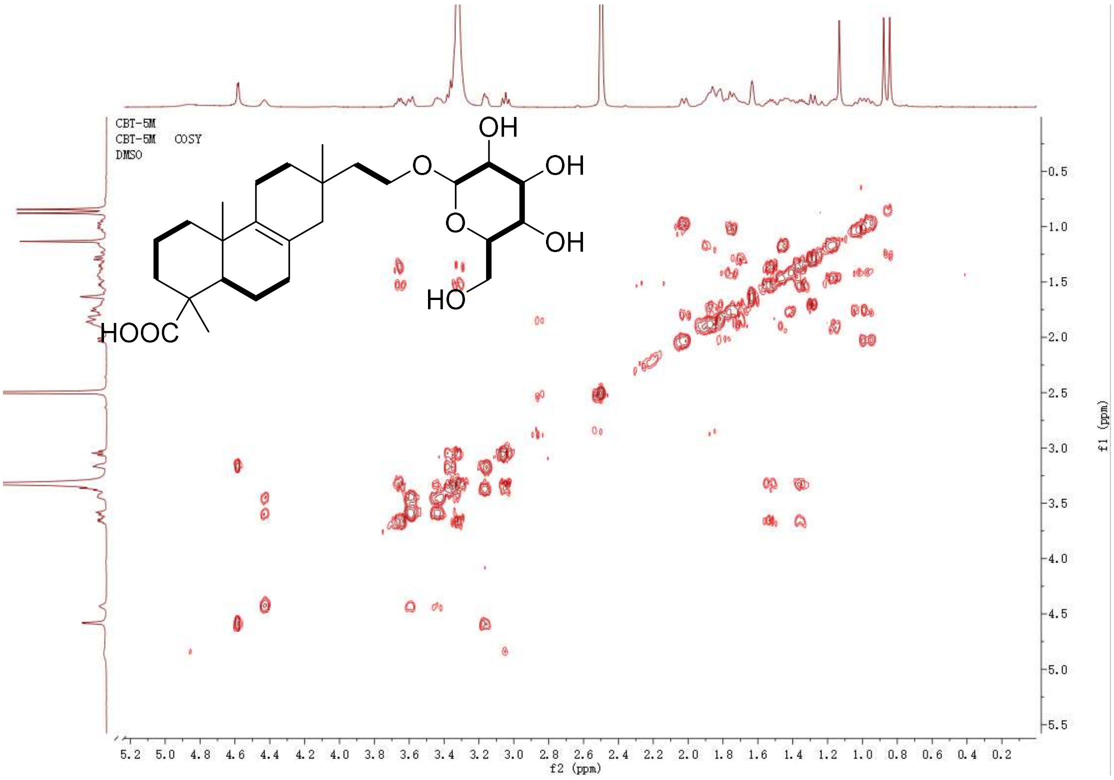

Fig. S20. HMBC spectrum of 3

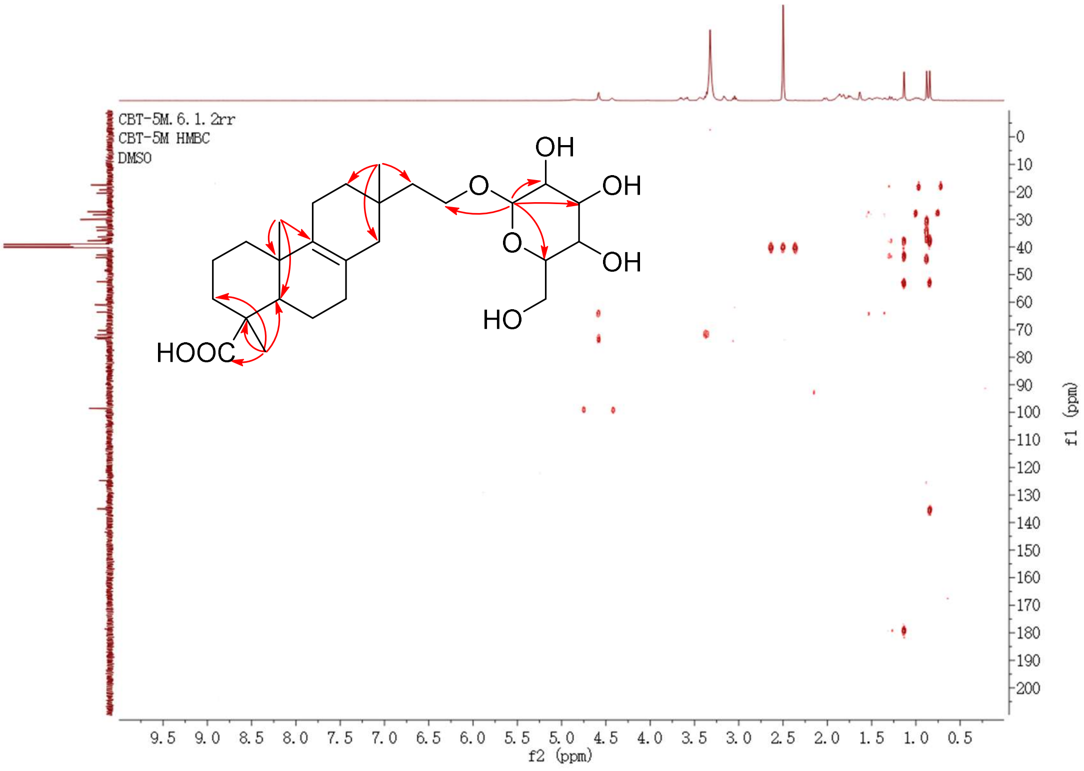

Fig. S21. ROESY spectrum of 3

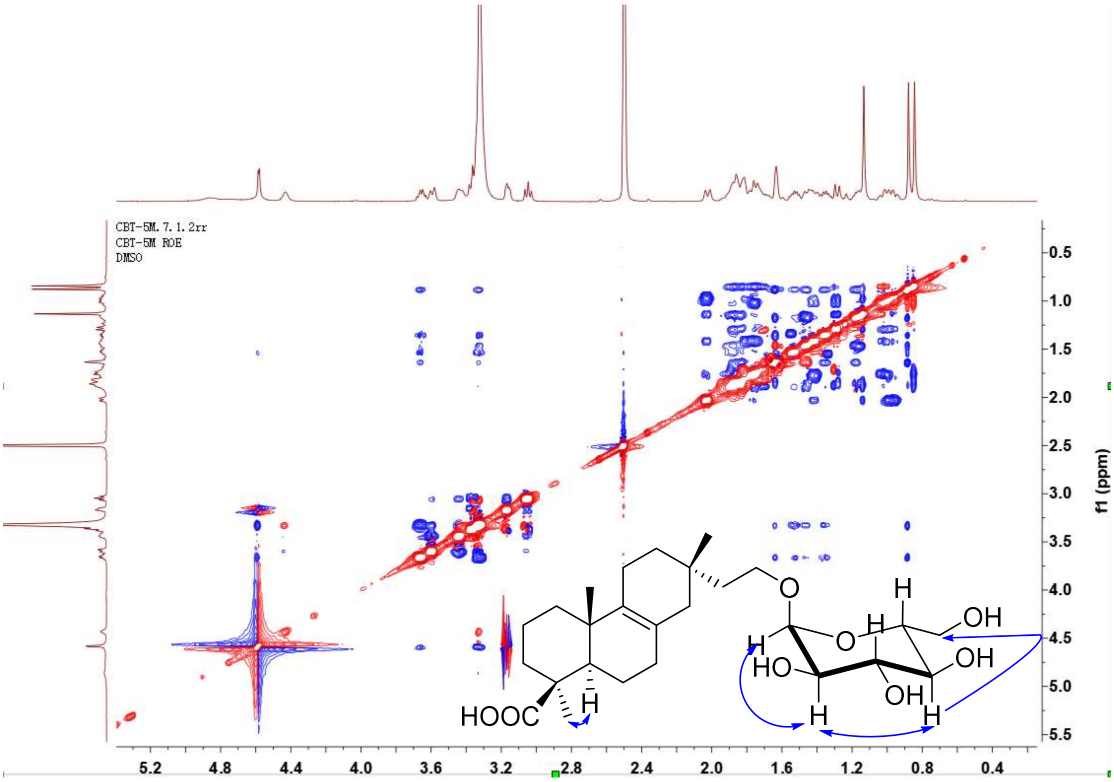

Fig. S22. HRESIMS report of 3

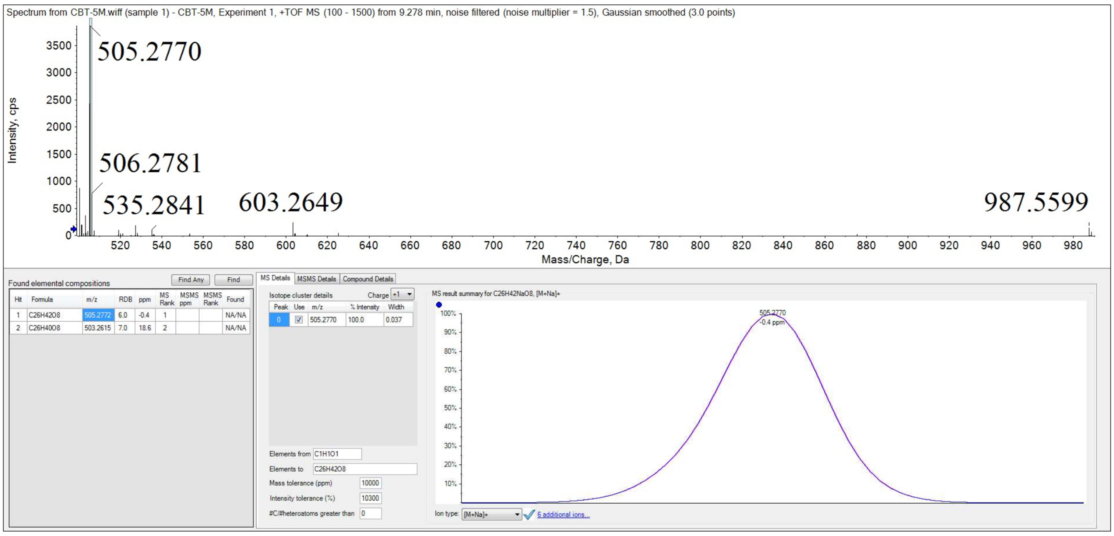

Fig. S23. ^{1}H NMR spectrum of 4 (500 MHz, DMSO-d_6)

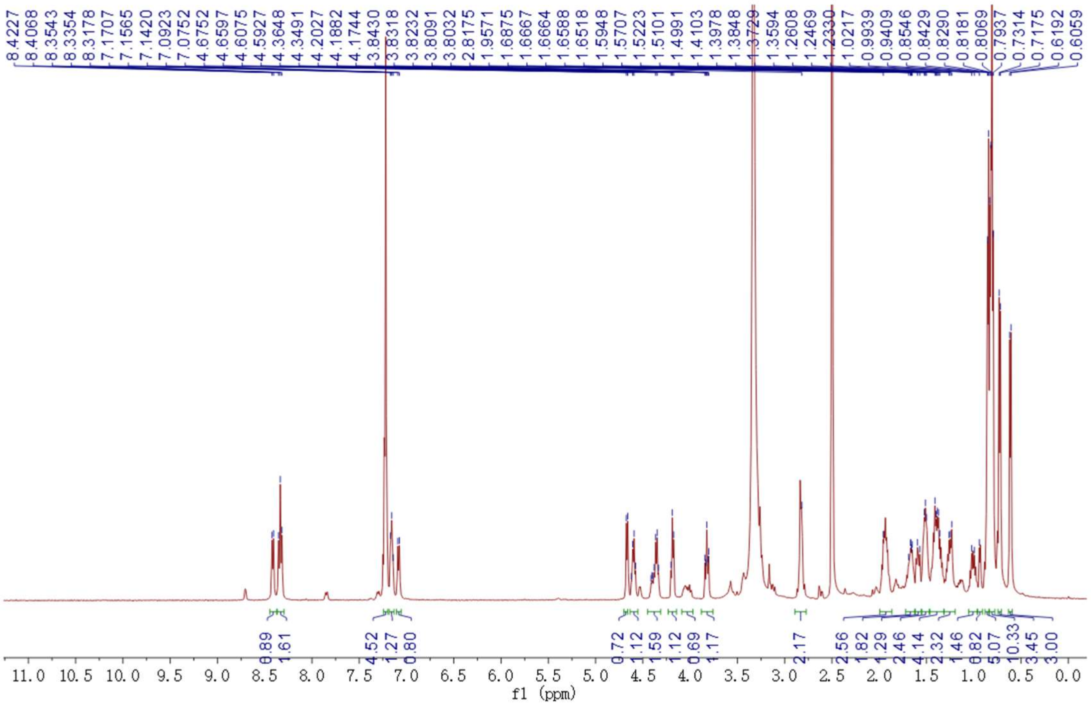

Fig. S24. ^{13}C and DEPT NMR spectra of 4 (125 MHz, DMSO-d_6)

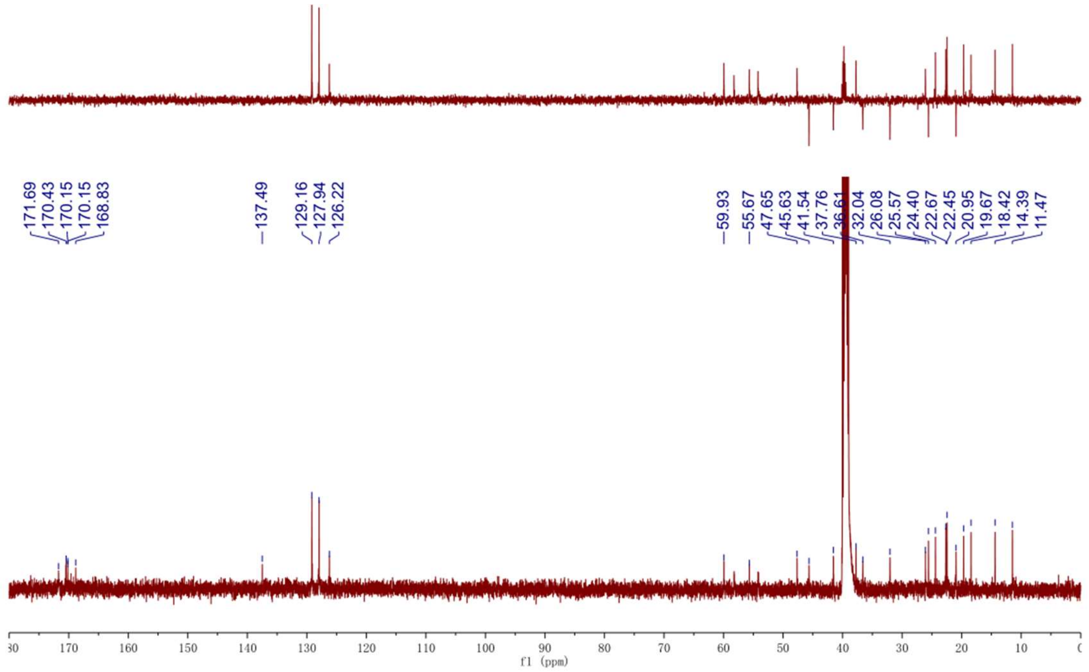

Fig. S25. HSQC spectrum of 4

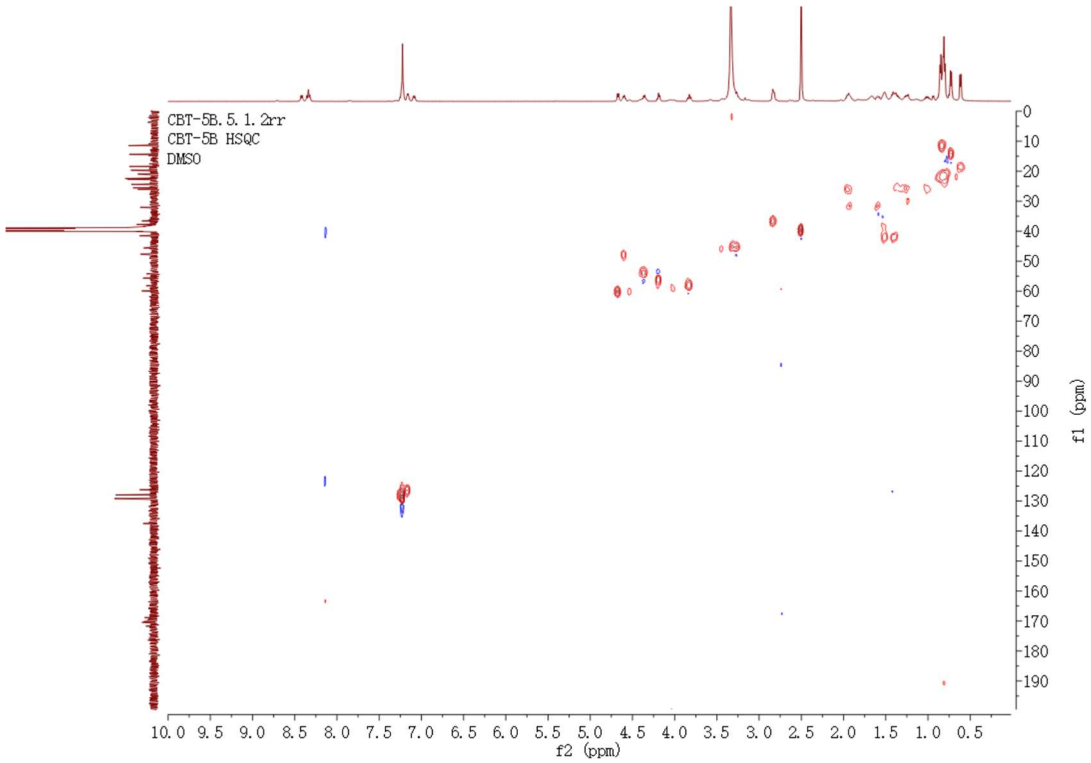

Fig. S26. ^{1}H-^{1}H COSY spectrum of 4

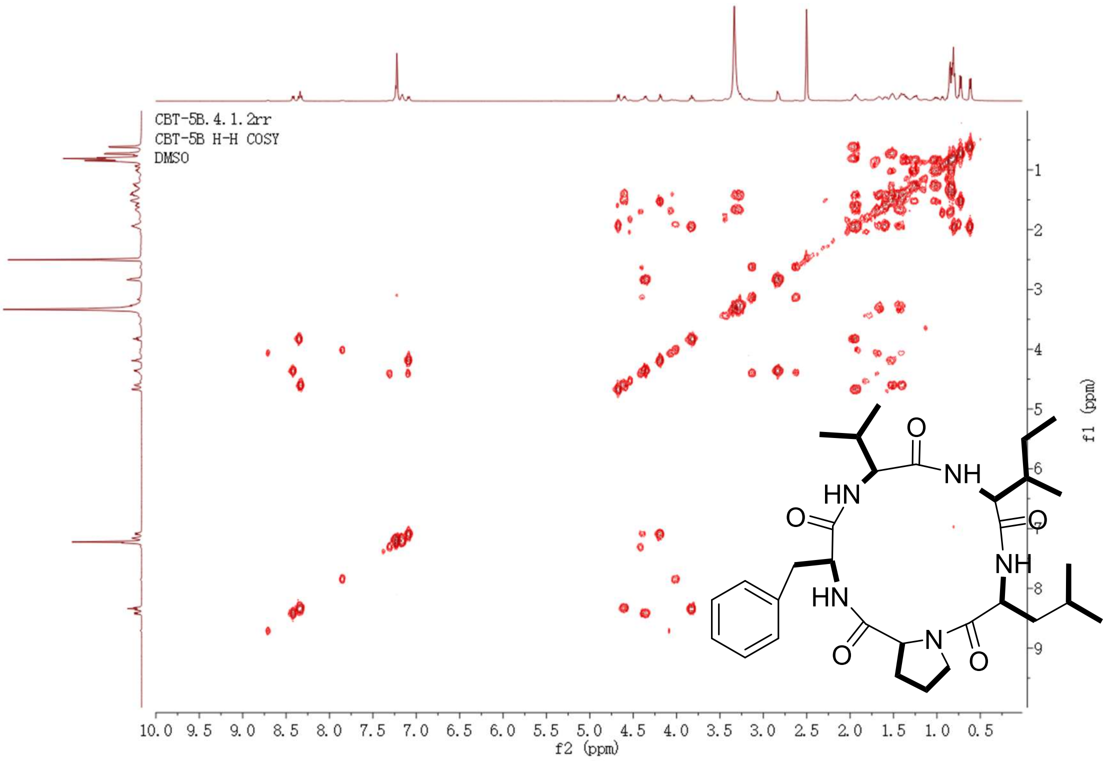

Fig. S27. HMBC spectrum of 4

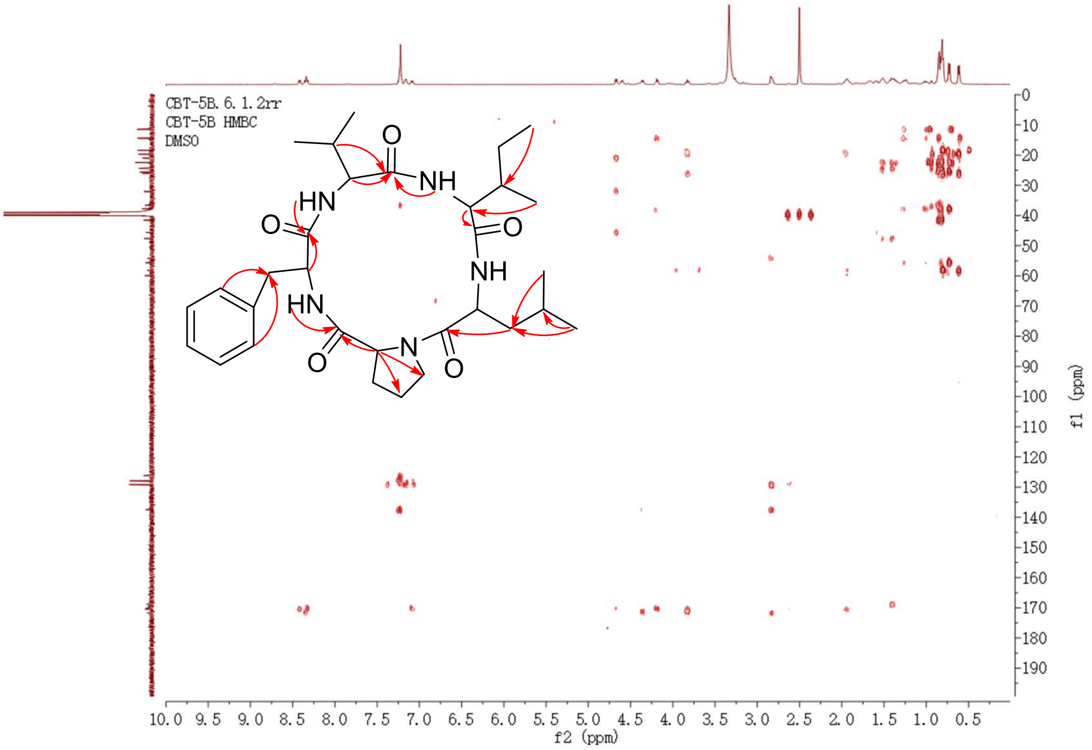

Fig. S28. ROESY spectrum of 4

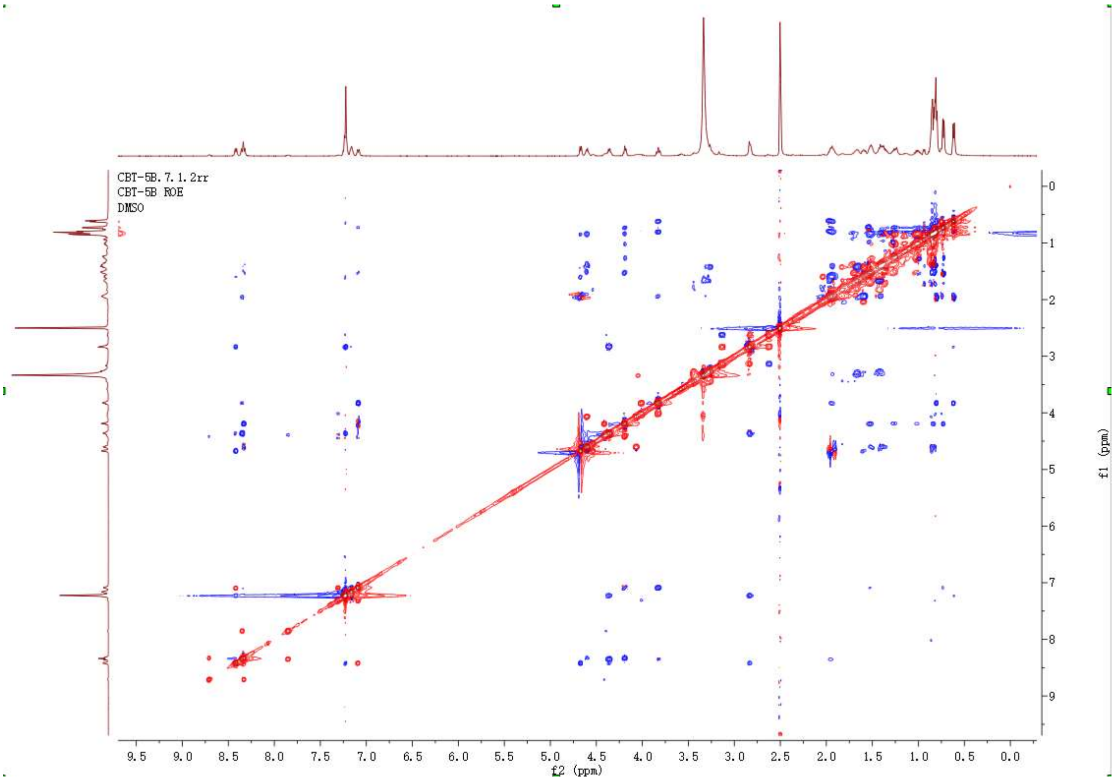

Fig. S29. HRESIMS report of 4

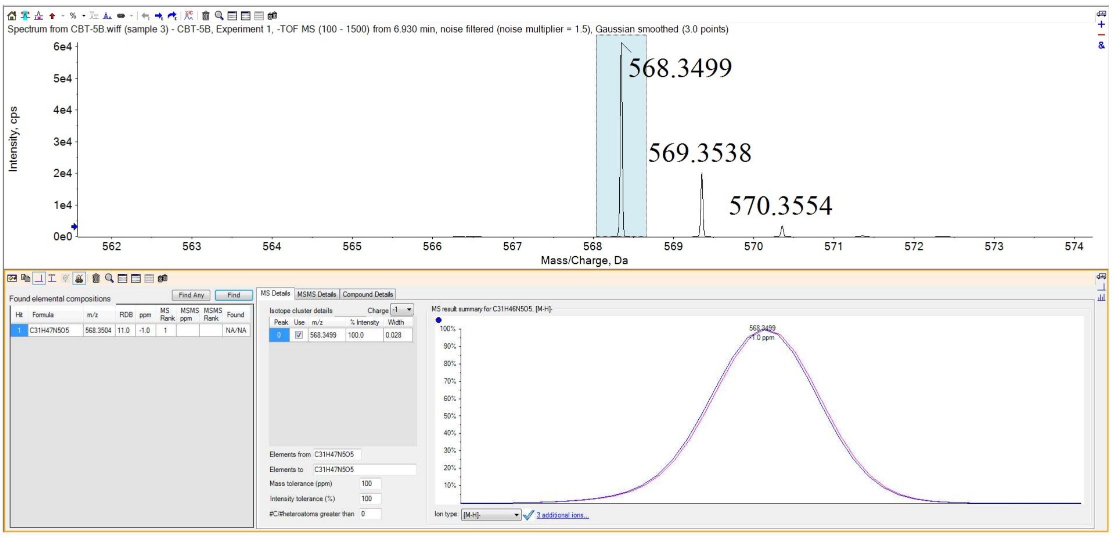

Fig. S30. ^{1}H NMR spectrum of 5 (500 MHz, CD_3OD)

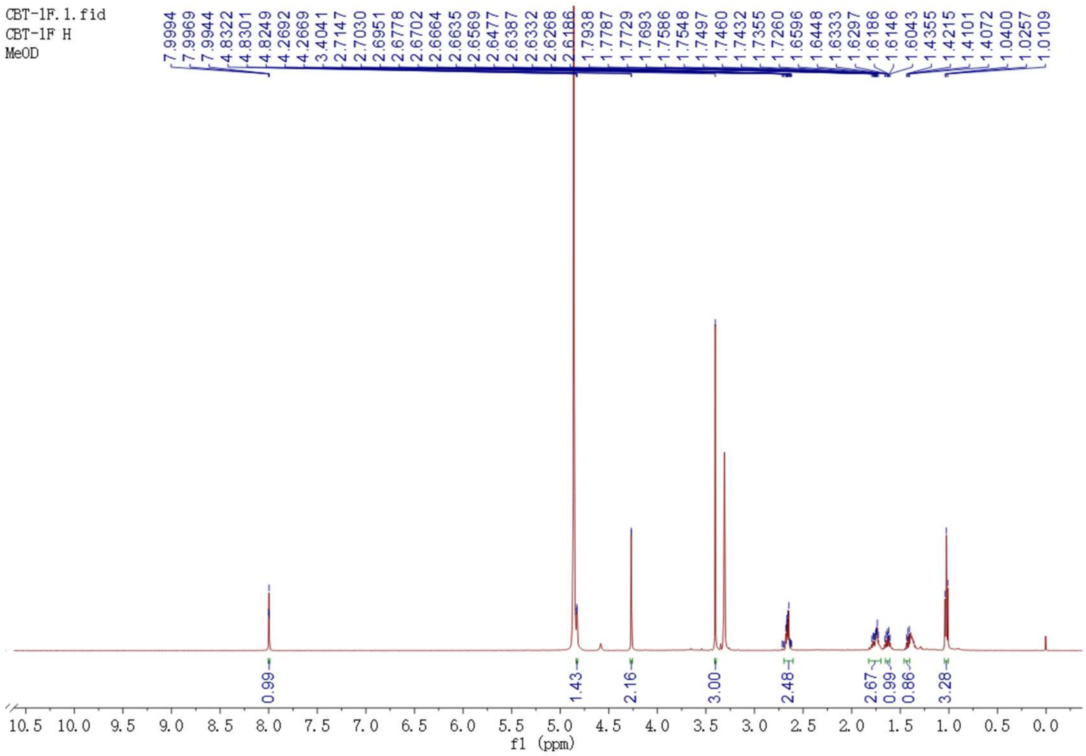

Fig. S31. ^{13}C NMR spectrum of 5 (125 MHz, CD_3OD)

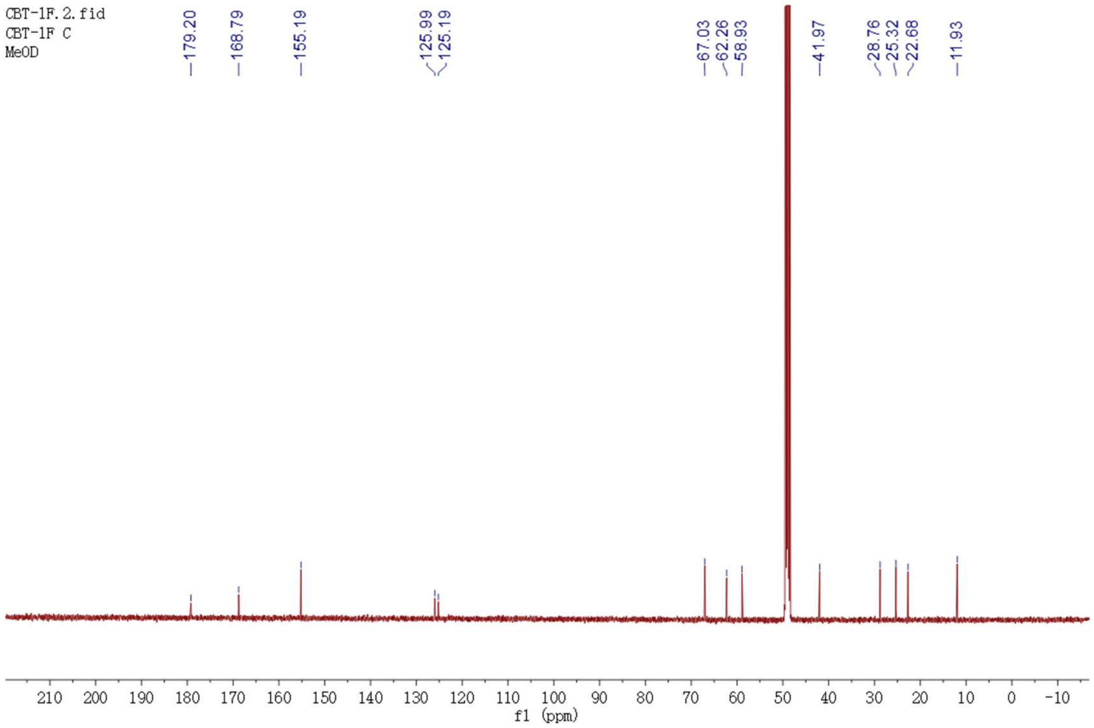

Fig. S32. HSQC spectrum of 5

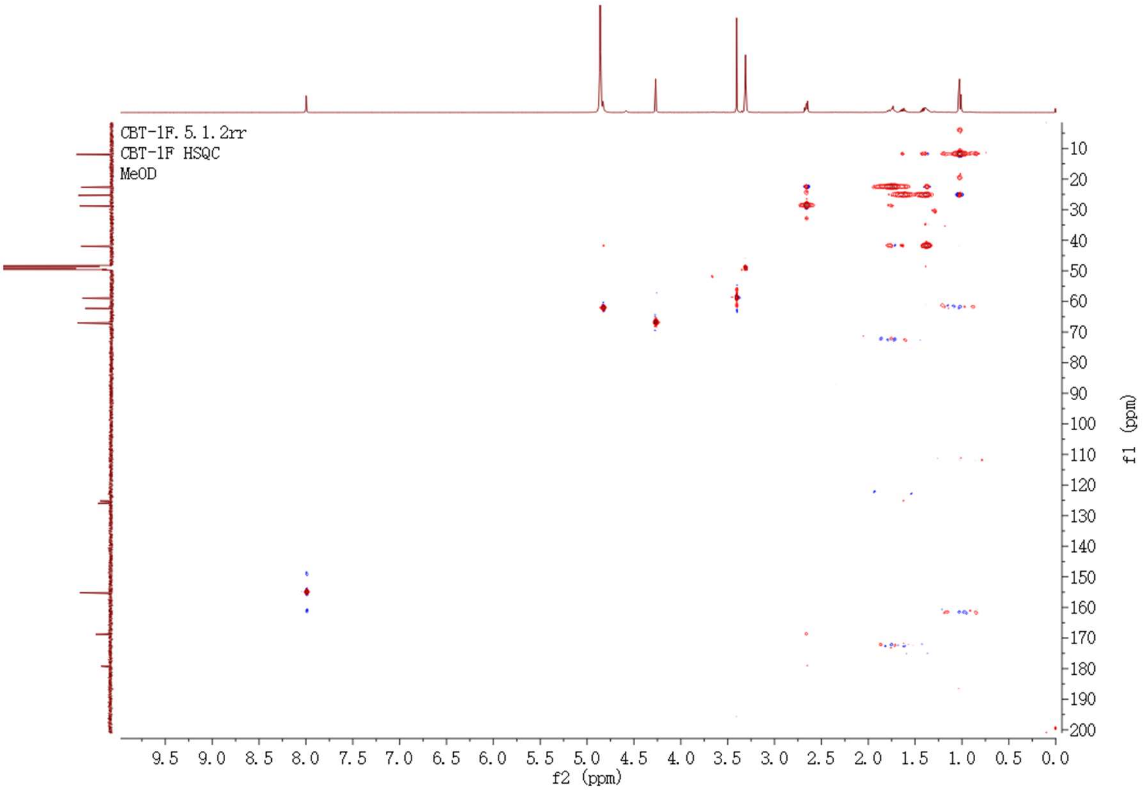

Fig. S33. HMBC spectrum of 5

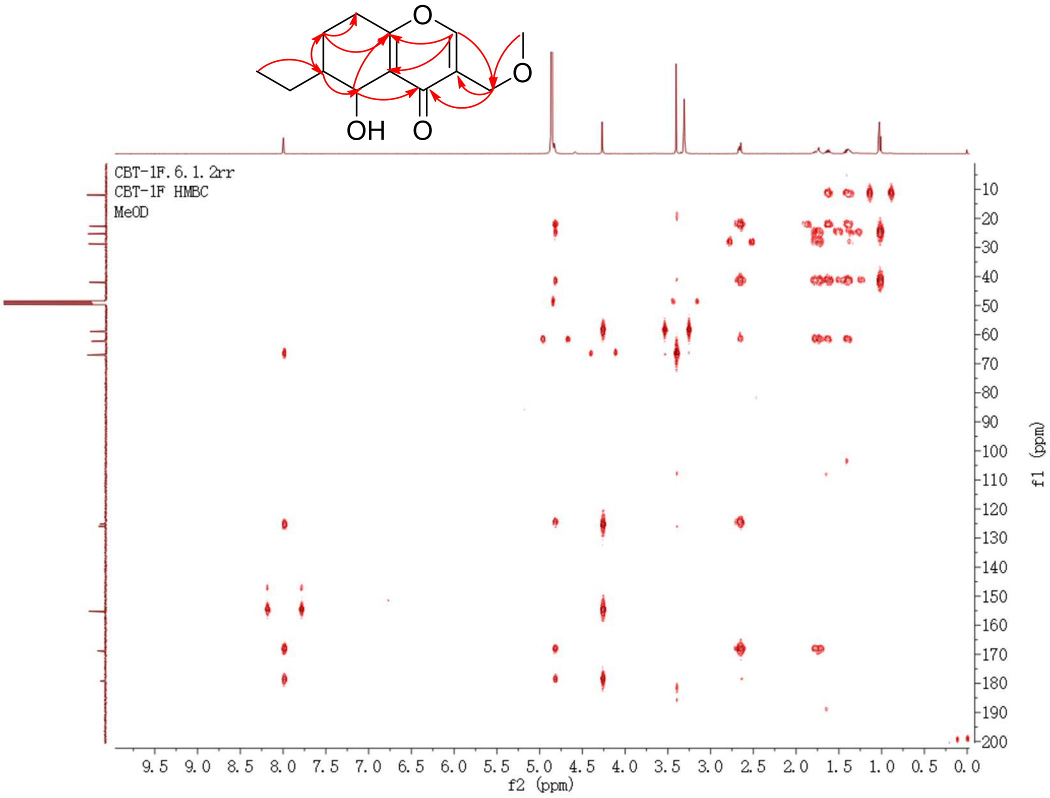

Fig. S34. ROESY spectrum of 5

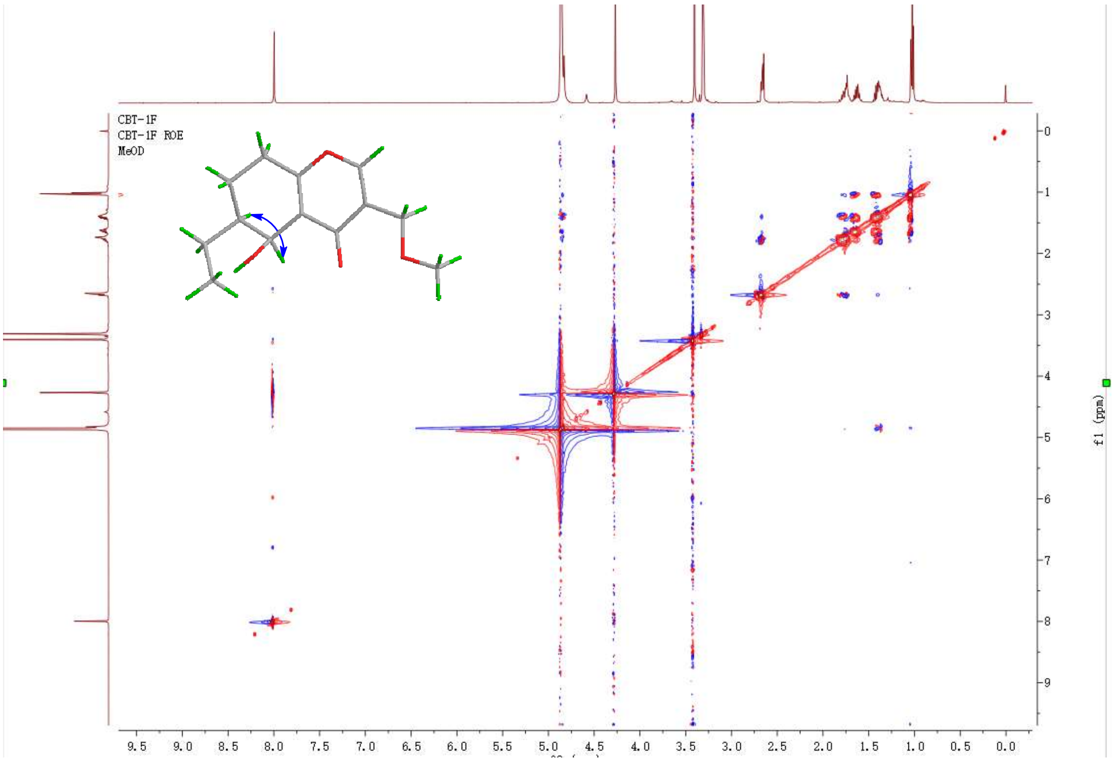

Fig. S35. HRESIMS report of 5

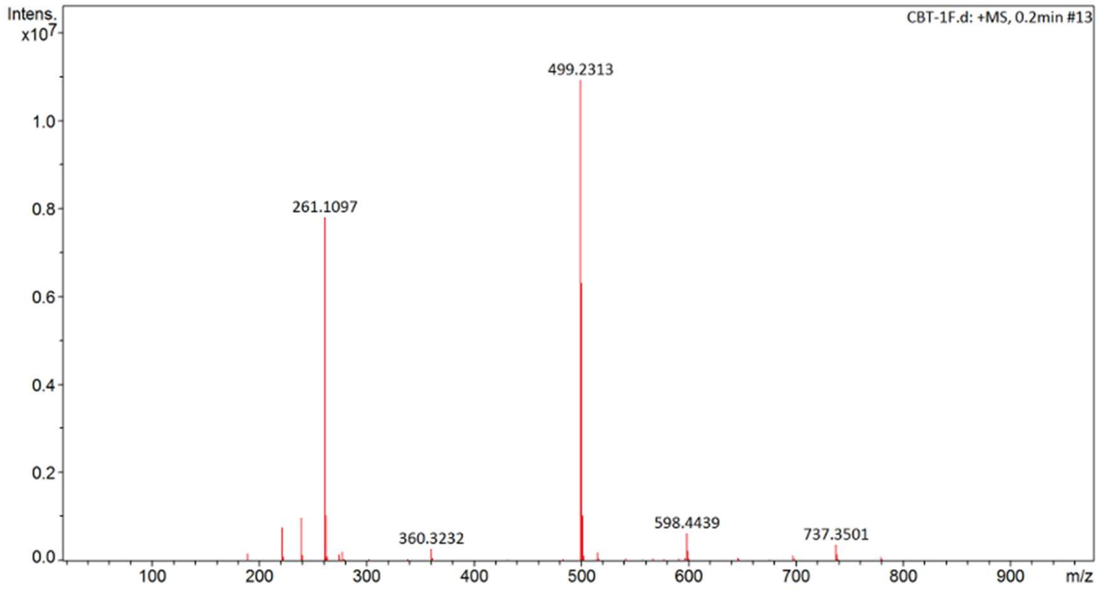

Bruker Compass DataAnalysis 4.4 printed: 4/1/2024 10:24:31 AM by: demo Page 1 of 1

Fig. S36. ^{1}H NMR spectrum of 6 (500 MHz, $CDCl_3$)

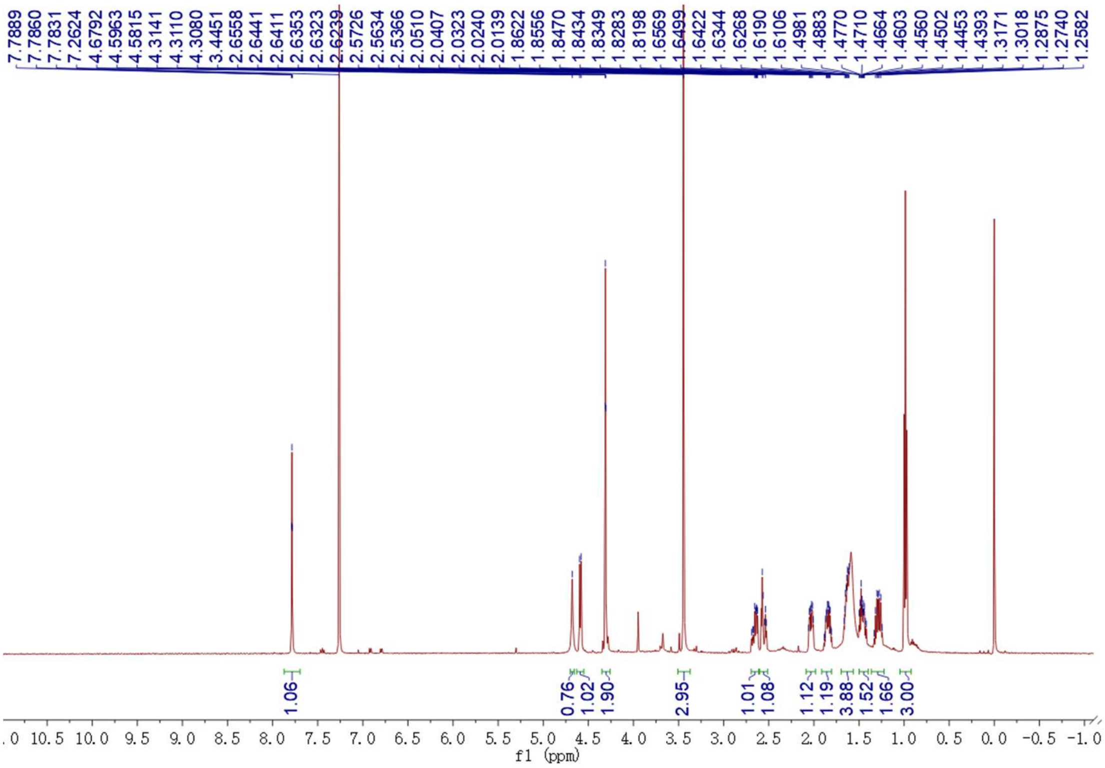

Fig. S37. ^{13}C NMR spectrum of 6 (125 MHz, $CDCl_3$)

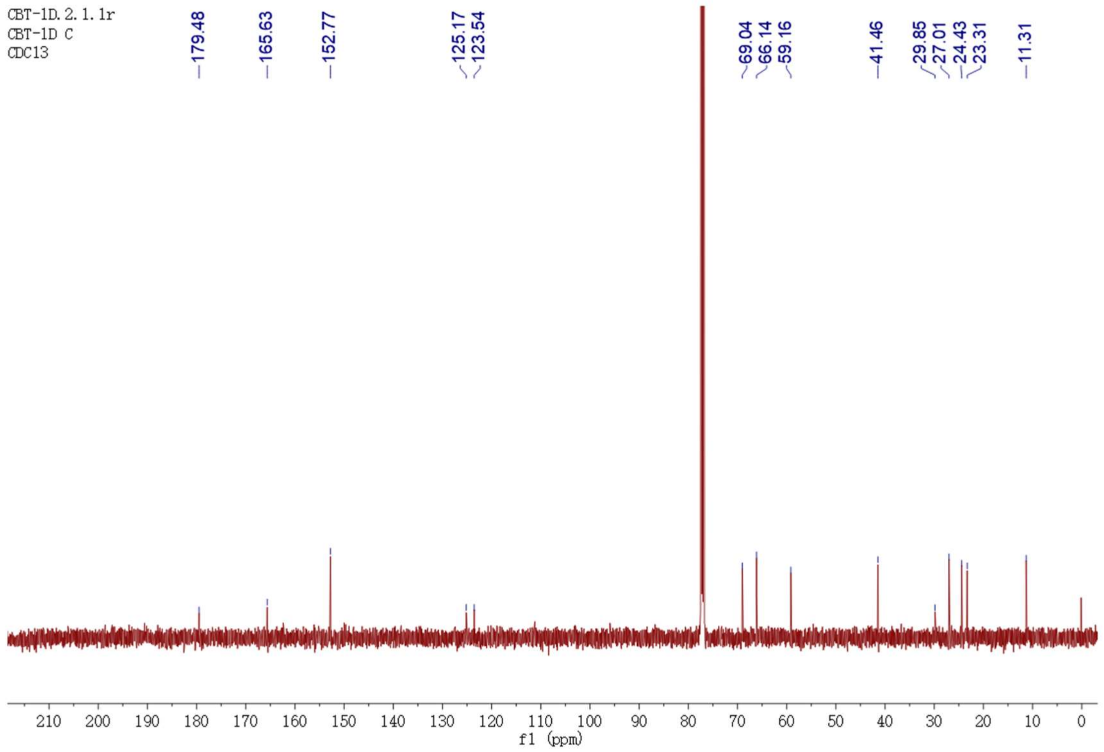

Fig. S38. HSQC spectrum of 6

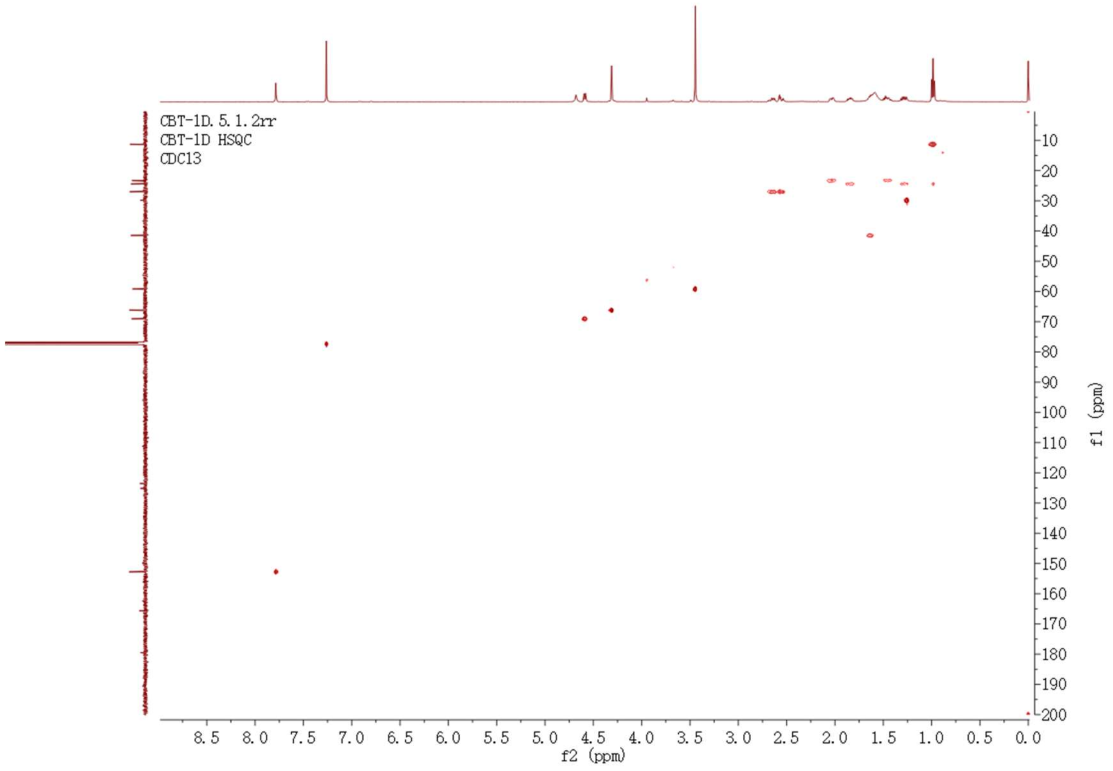

Fig. S39. ^{1}H-^{1}H COSY spectrum of 6

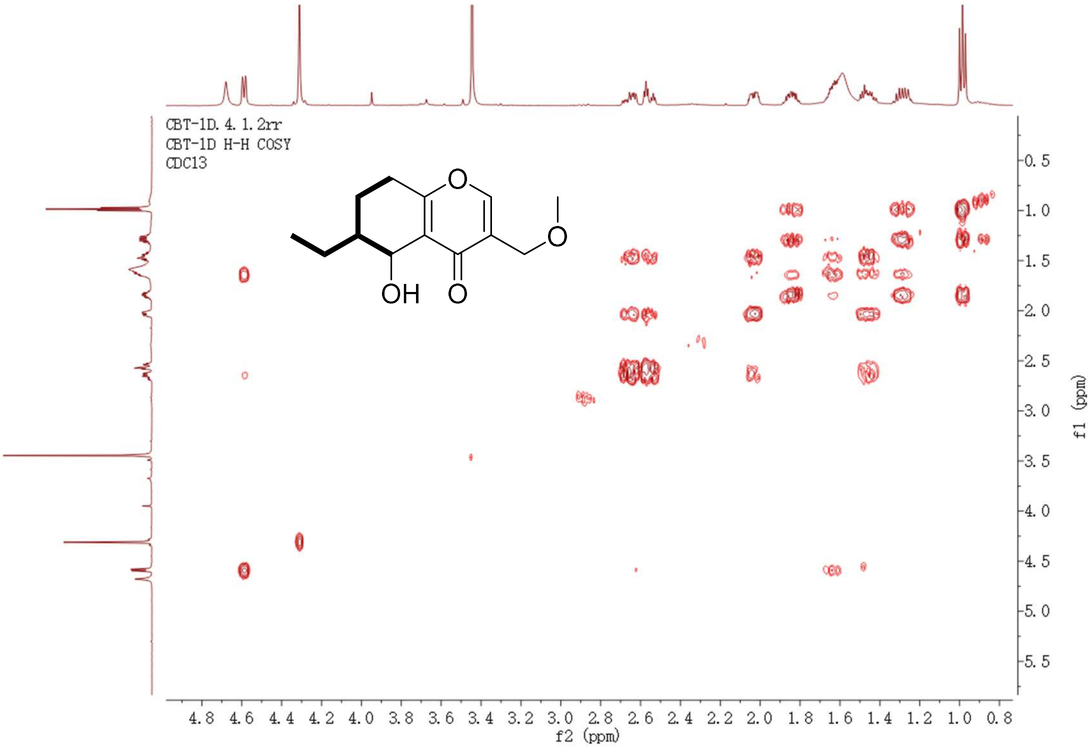

Fig. S40. HMBC spectrum of 6

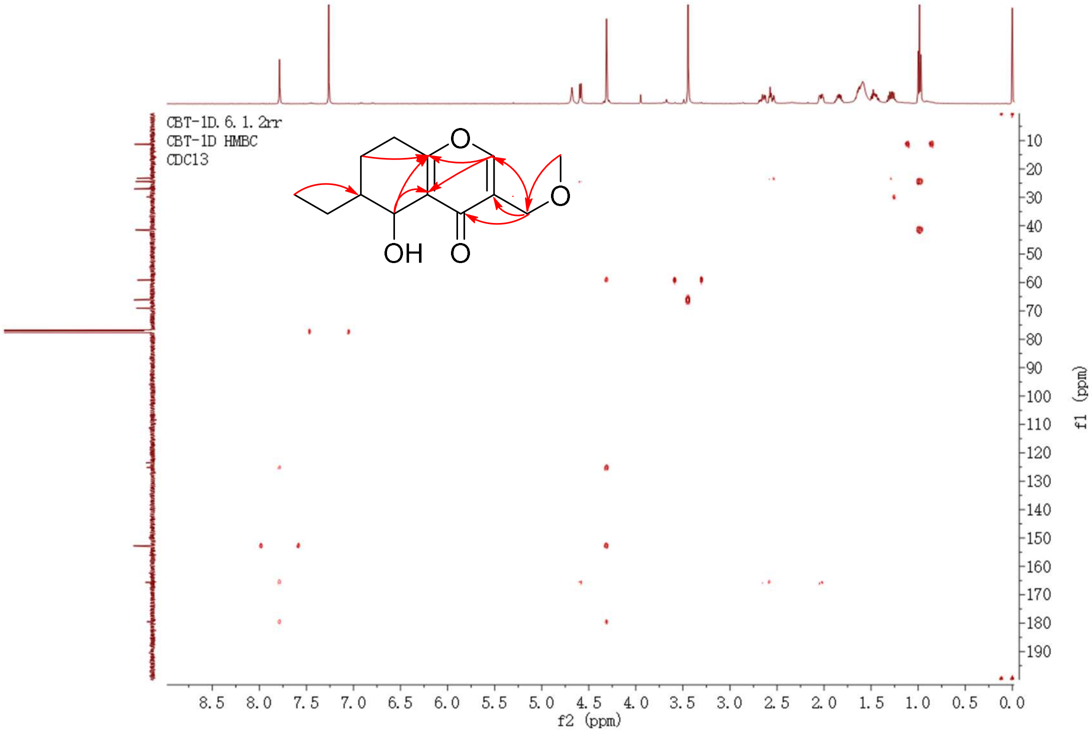

Fig. S41. ROESY spectrum of 6

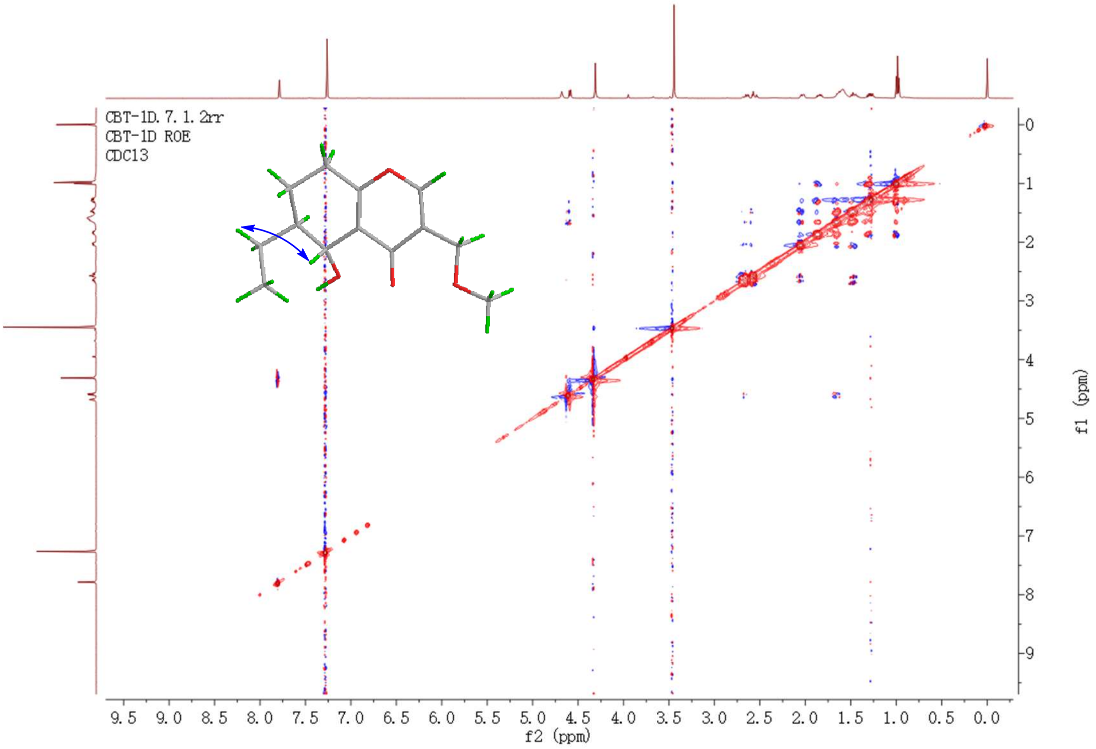

Fig. S42. HRESIMS report of 6

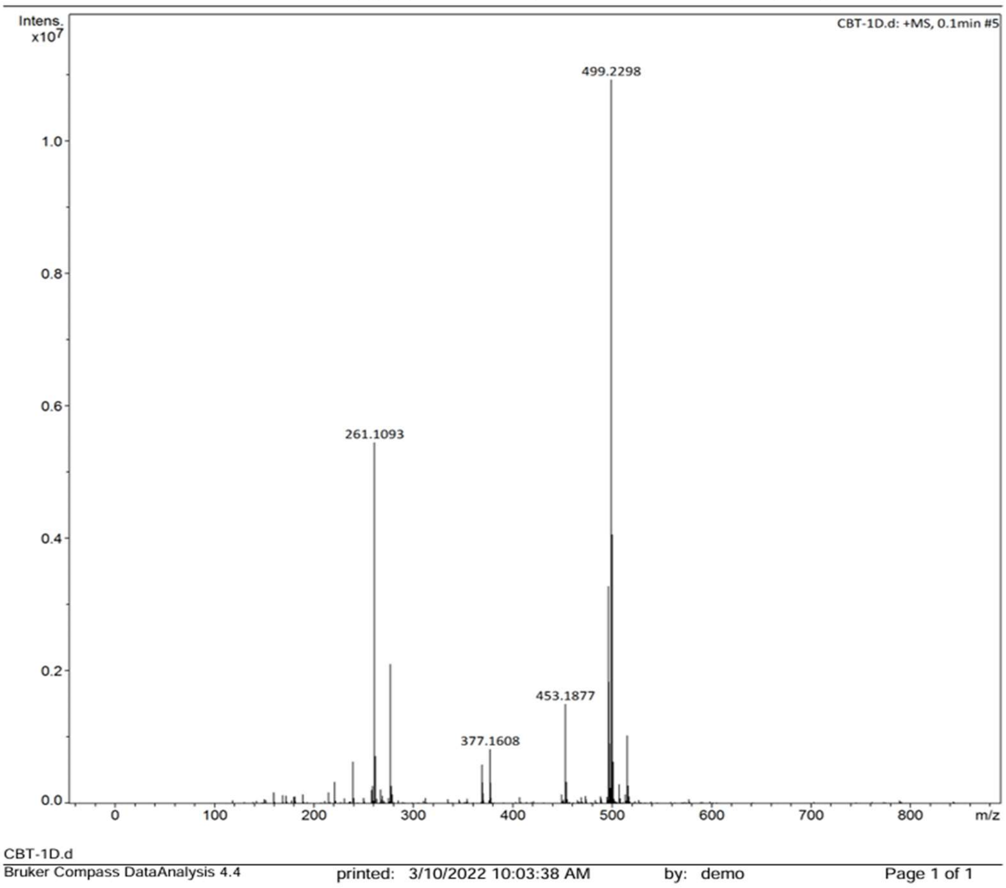

Fig. S43. ^{1}H NMR spectrum of 7 (500 MHz, $CDCl_3$)

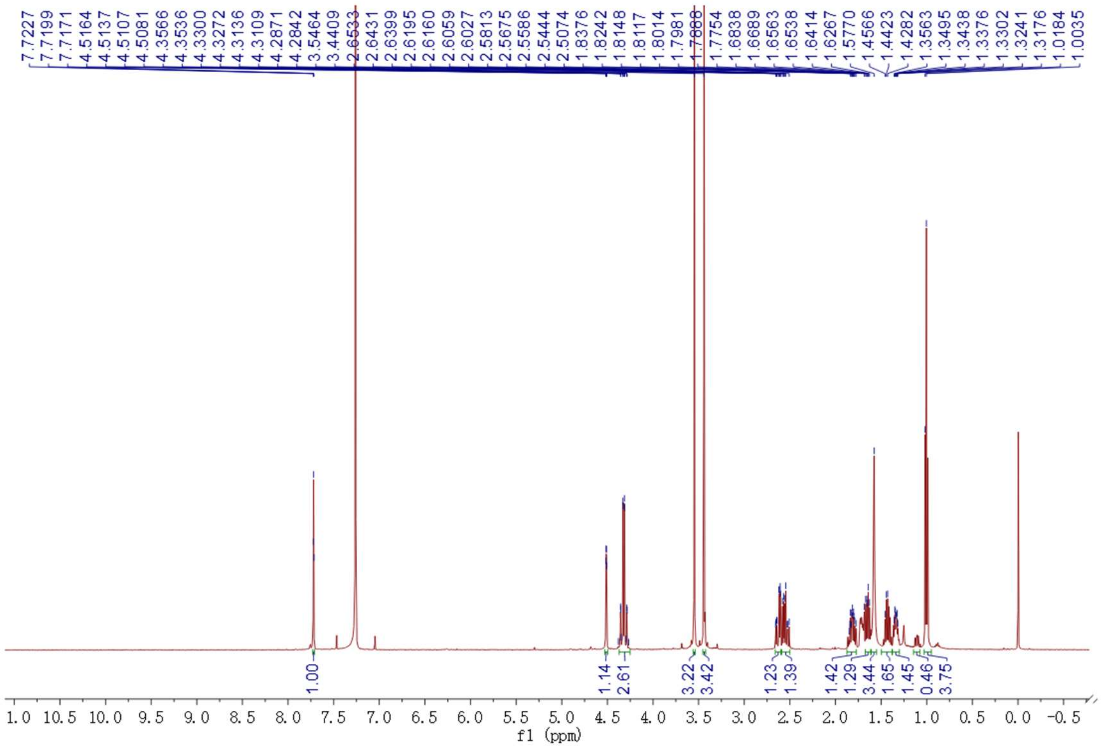

Fig. S44. ^{13}C NMR spectrum of 7 (125 MHz, $CDCl_3$)

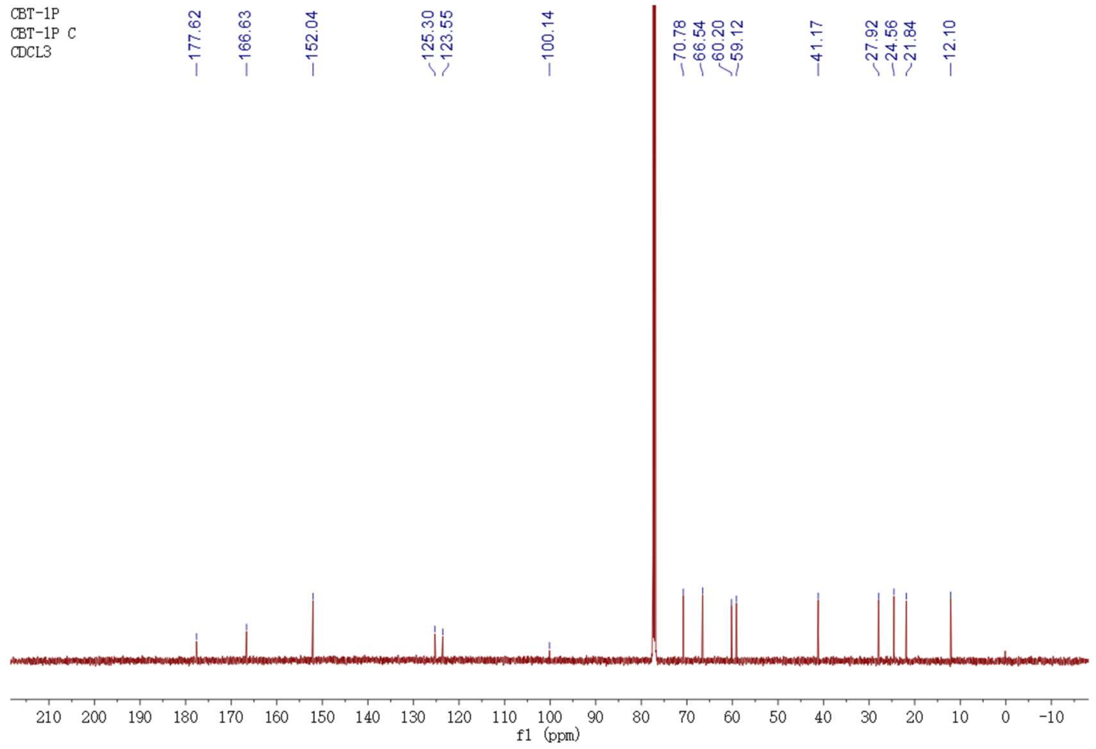

Fig. S45. HSQC spectrum of 7

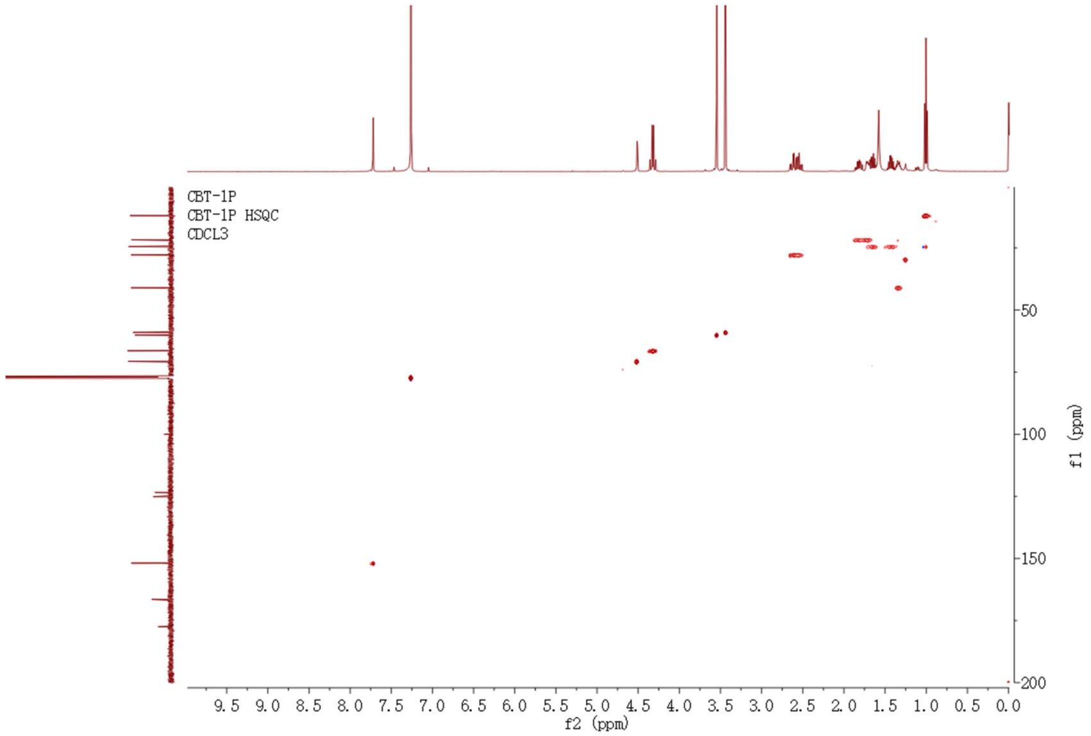

Fig. S46. HMBC spectrum of 7

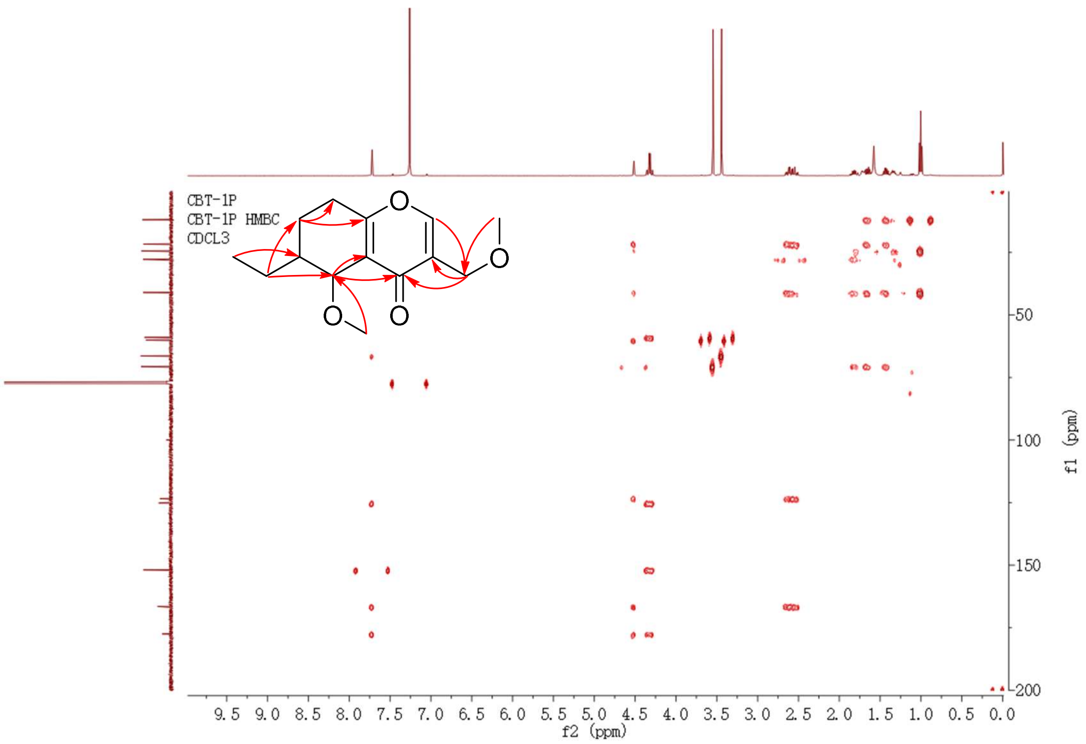

Fig. S47. ROESY spectrum of 7

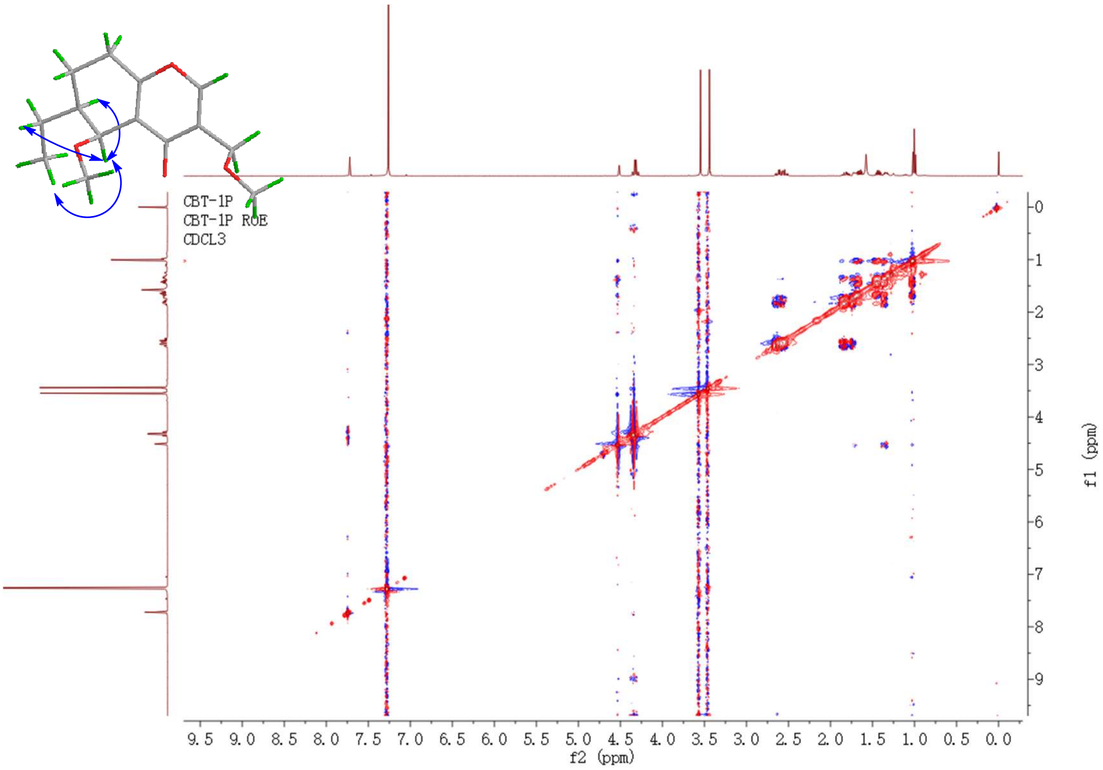

Fig. S48. HRESIMS report of 7

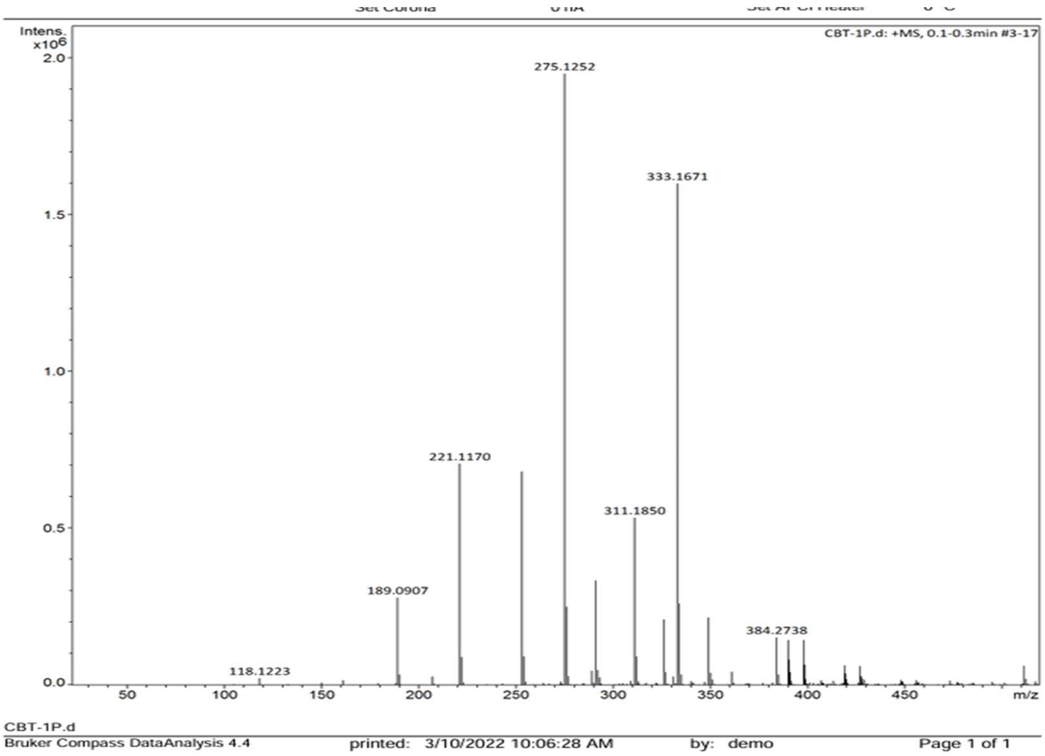

Fig. S49. ^{1}H NMR spectrum of 8 (500 MHz, $CDCl_3$)

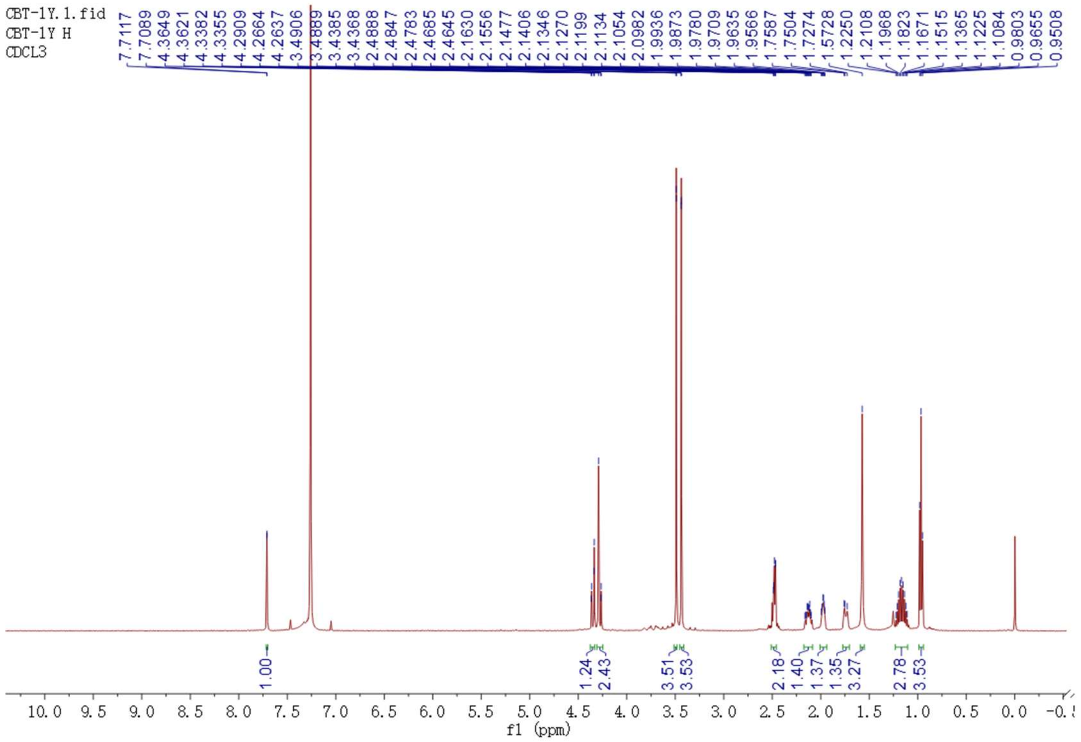

Fig. S50. ^{13}C NMR spectrum of 8 (125 MHz, $CDCl_3$)

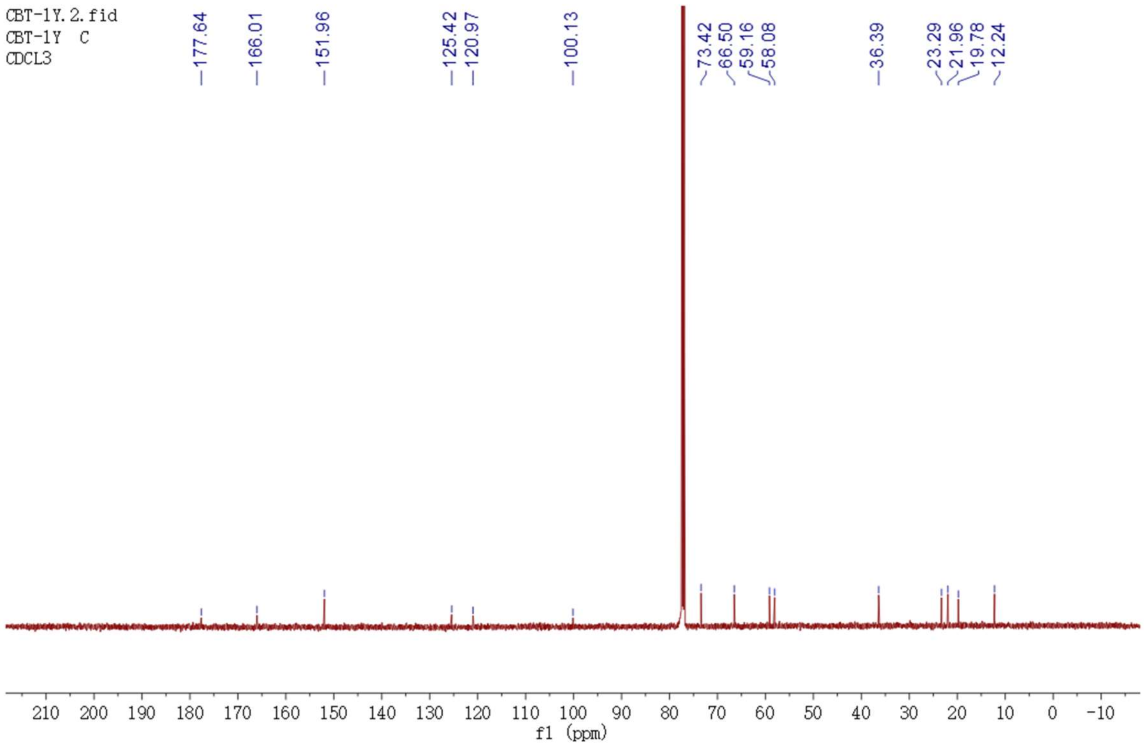

Fig. S51. HSQC spectrum of 8

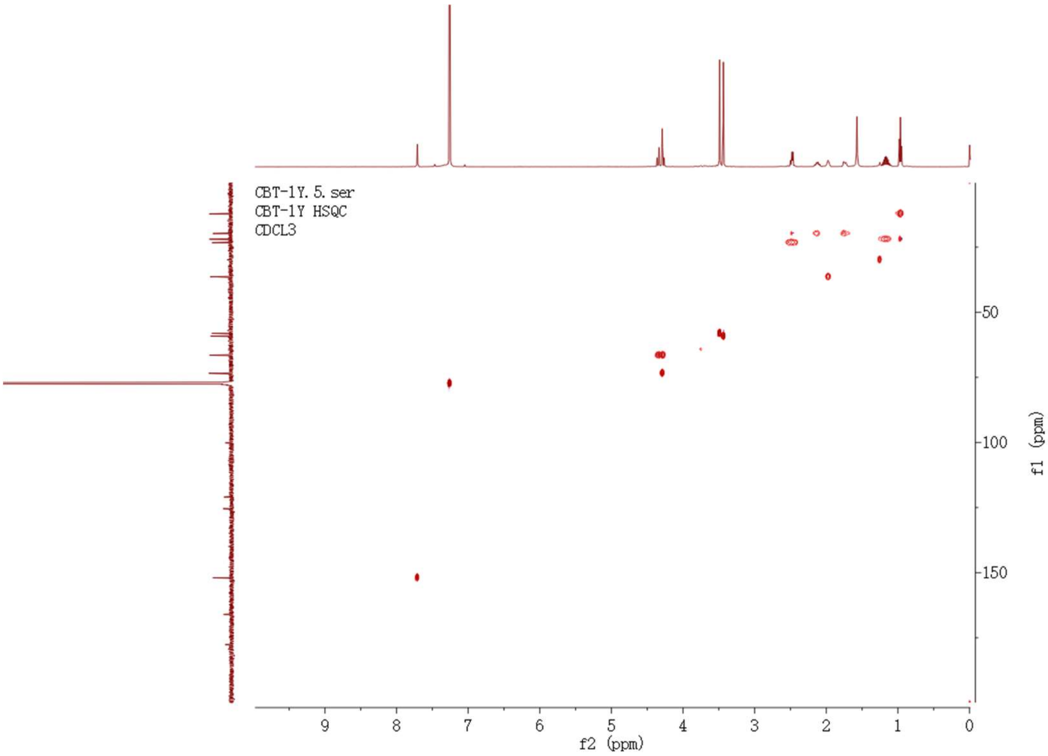

Fig. S52. ^{1}H-^{1}H COSY spectrum of 8

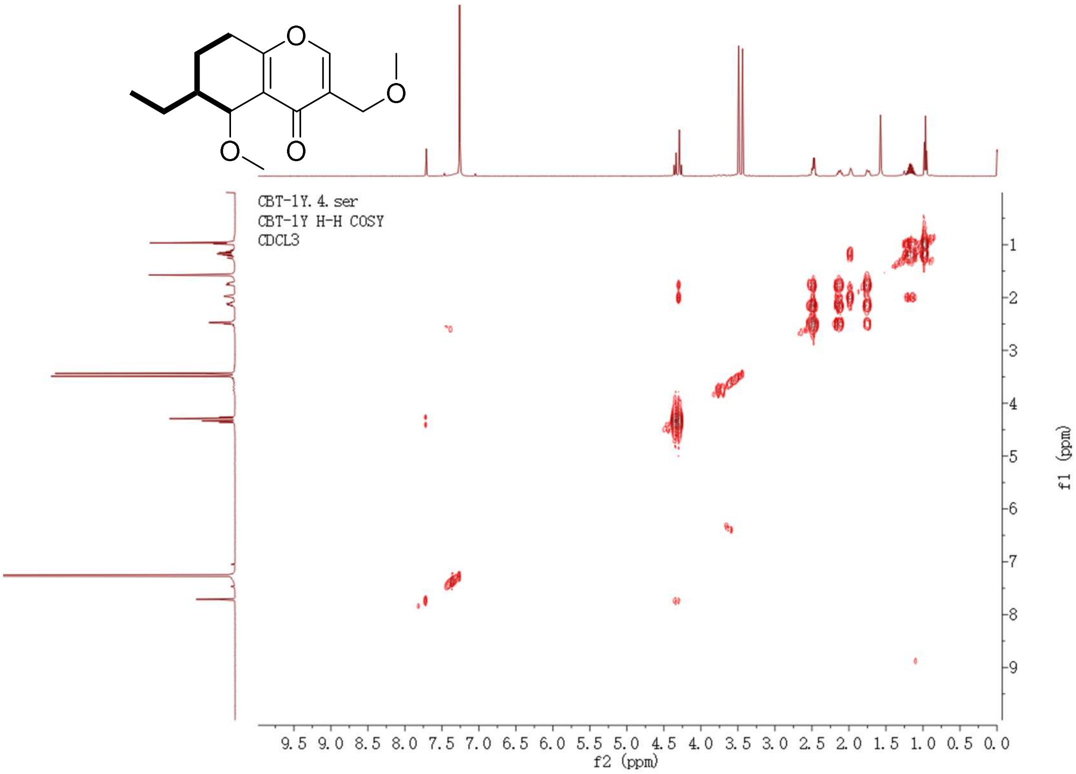

Fig. S53. HMBC spectrum of 8

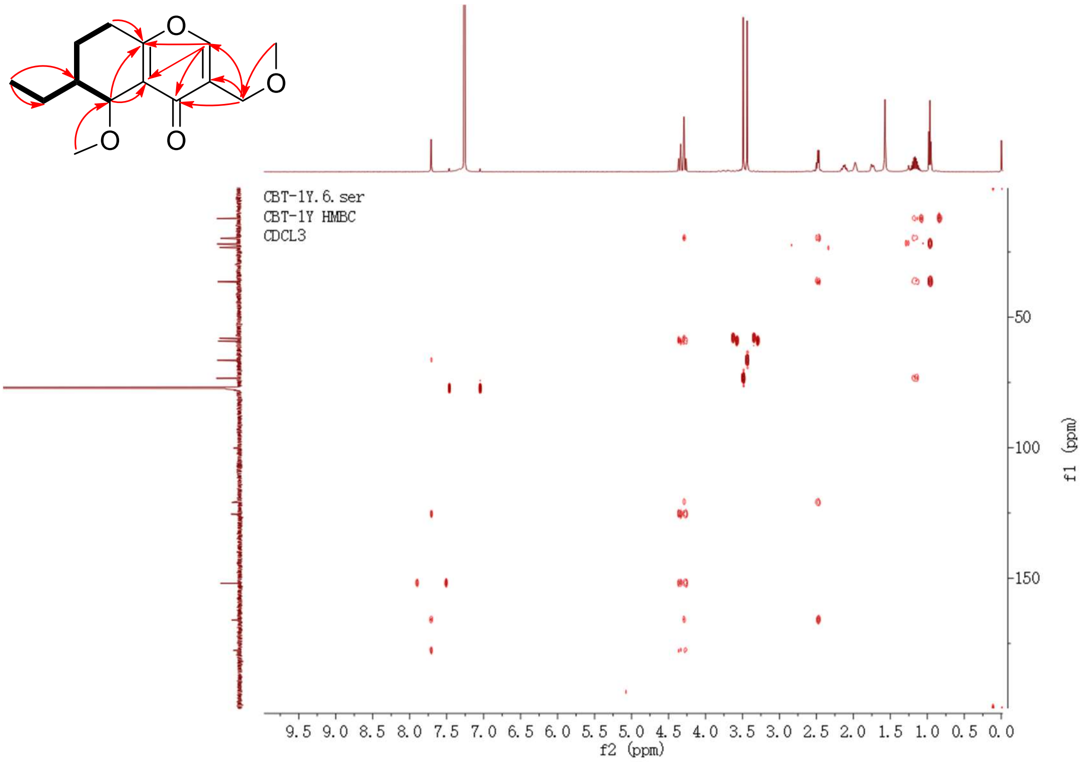

Fig. S54. ROESY spectrum of 8

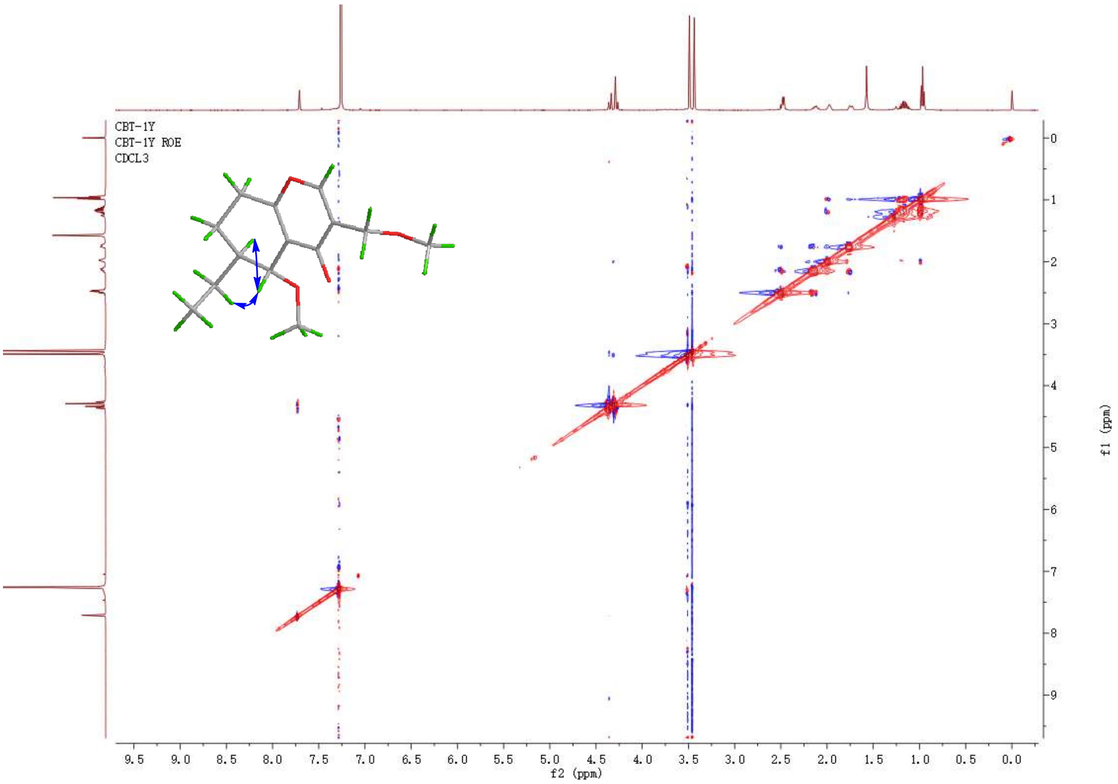

Fig. S55. HRESIMS report of 8

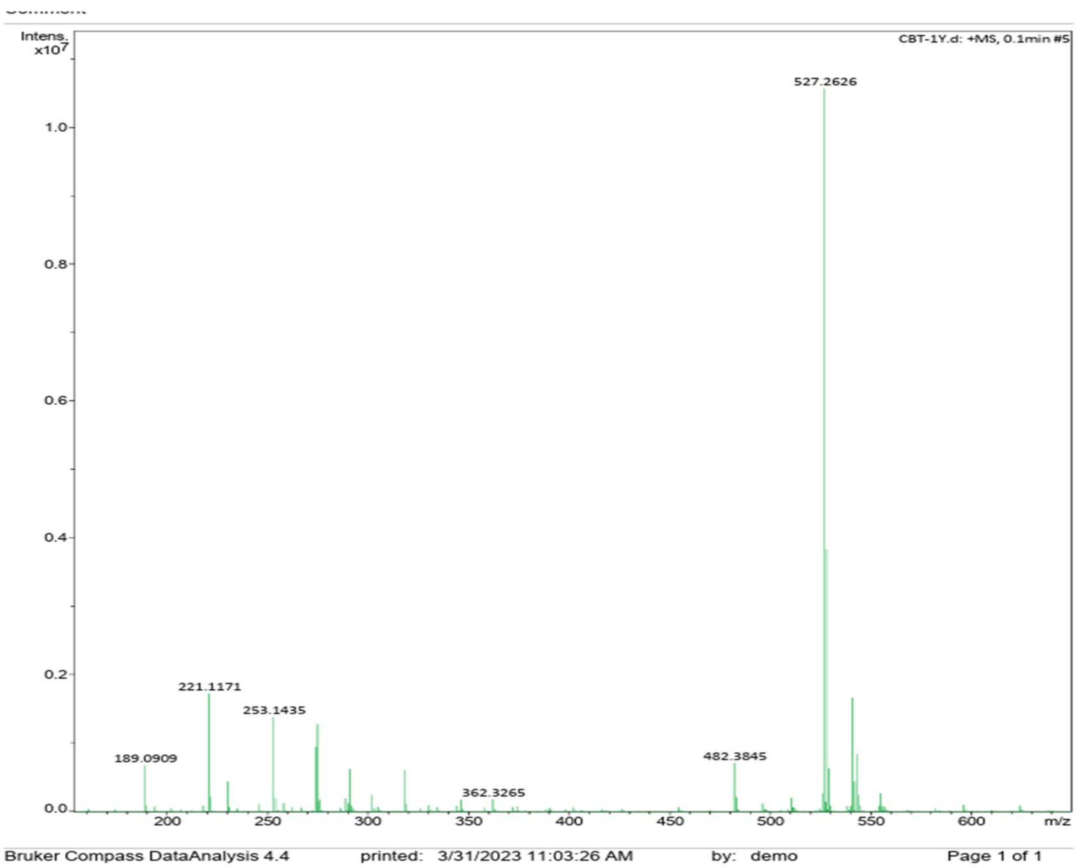

Fig. S56. ^{1}H NMR spectrum of 9 (500 MHz, $CDCl_3$)

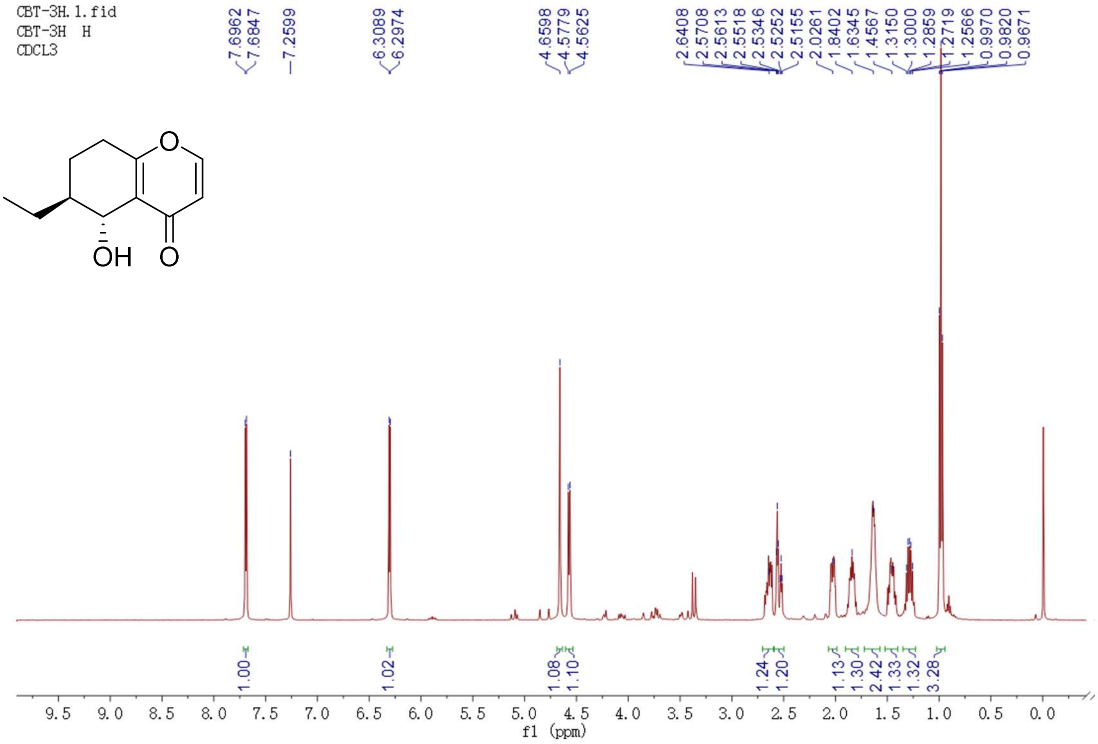

Fig. S57. ^{13}C NMR spectrum of 9 (125 MHz, $CDCl_3$)

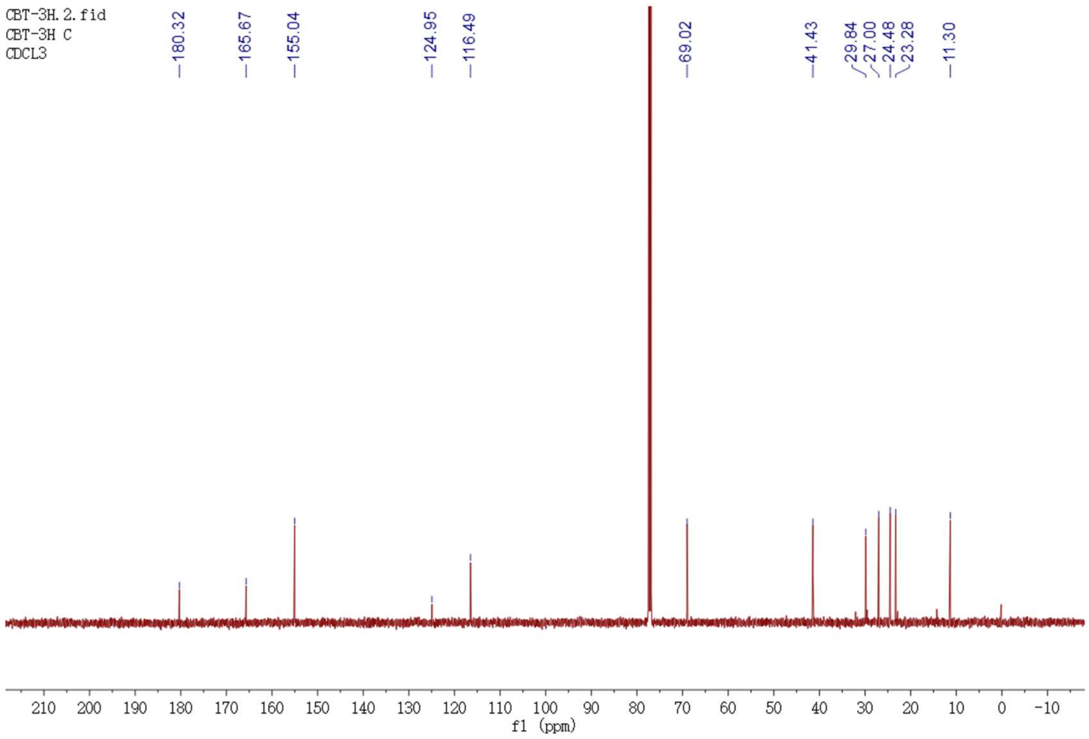

Fig. S58. HSQC spectrum of 9

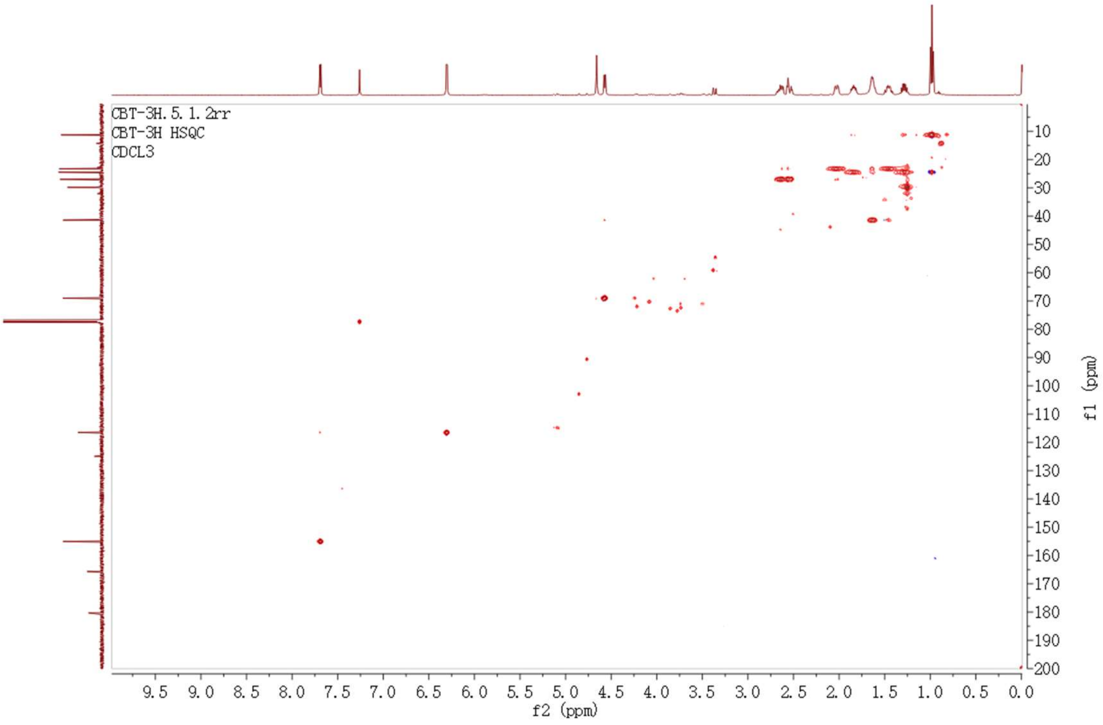

Fig. S59. ^1H-^1H COSY spectrum of 9

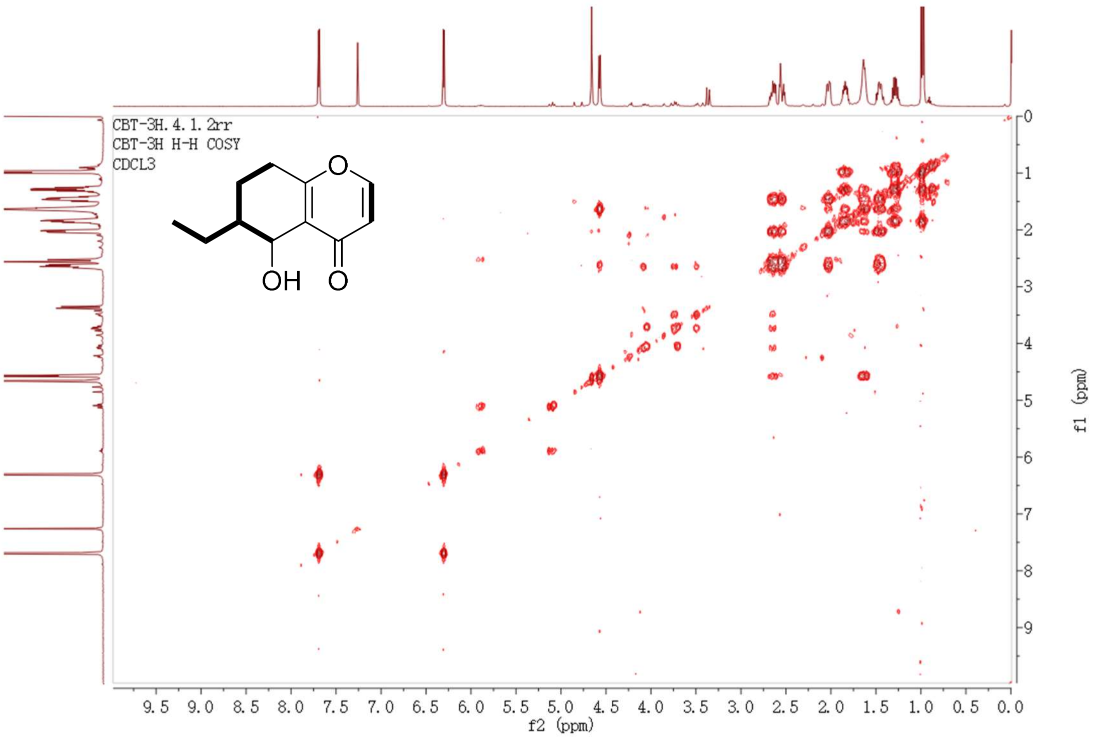

Fig. S60. HMBC spectrum of 9

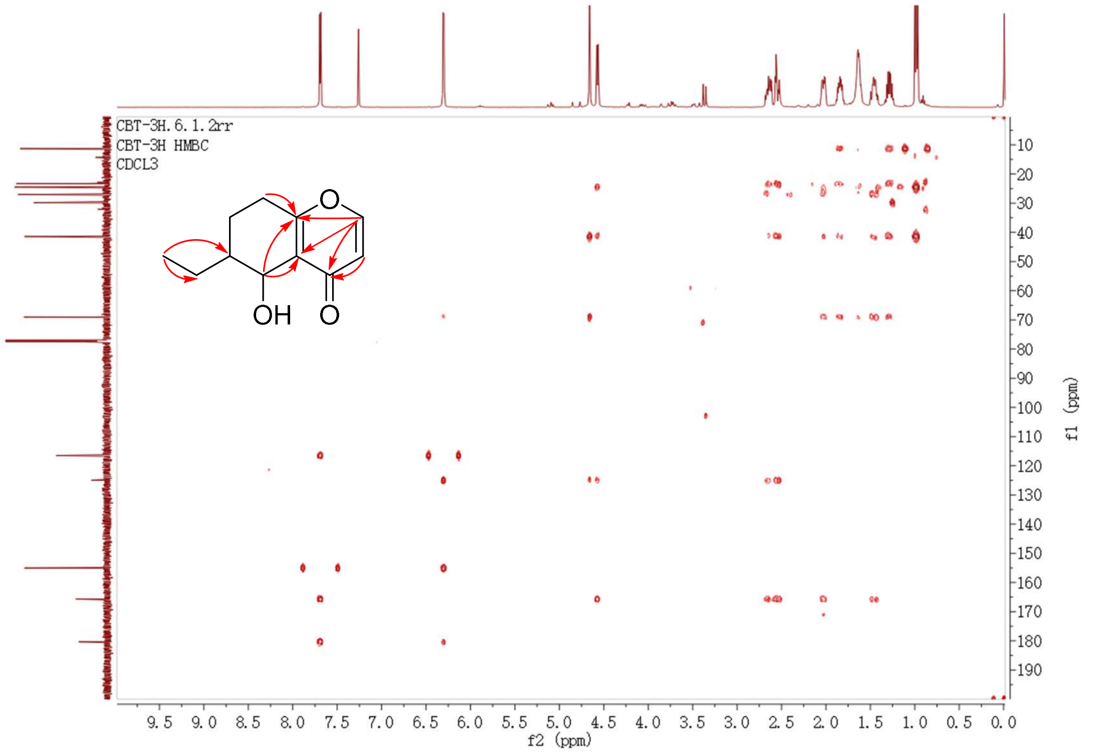

Fig. S61. ROESY spectrum of 9

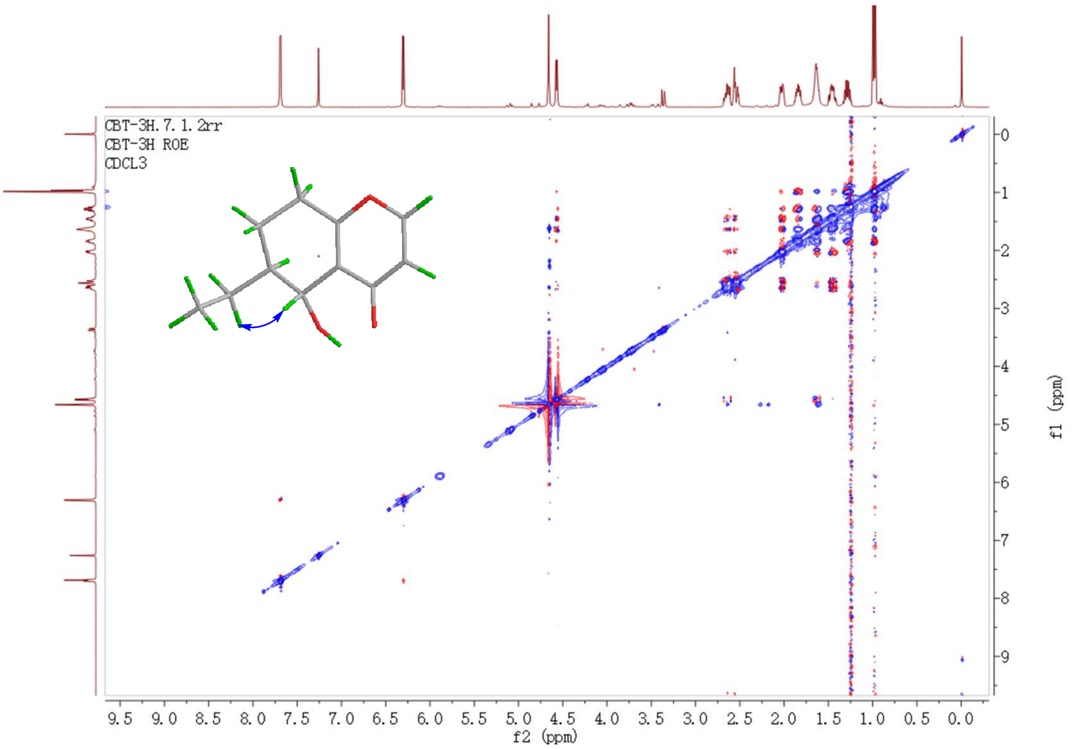

Fig. S62. HRESIMS report of 9

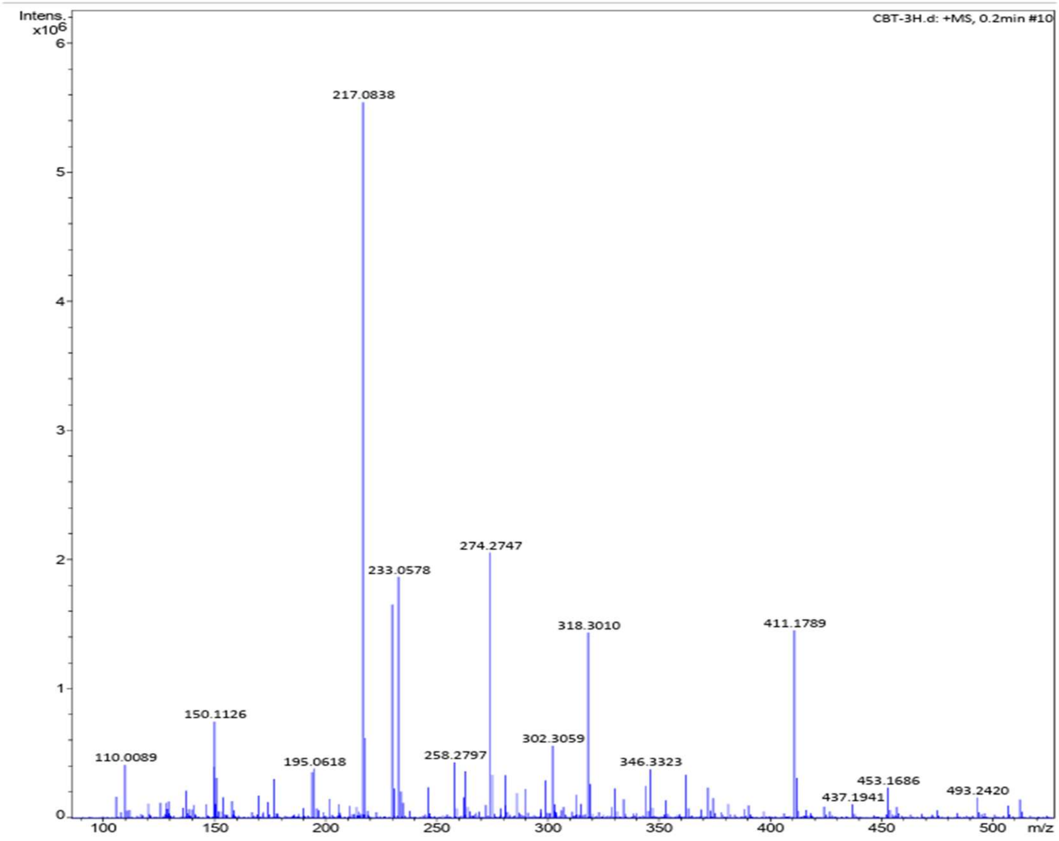

Bruker Compass DataAnalysis 4.4 printed: 3/31/2023 11:09:36 AM by: demo Page 1 of 1

Fig. S63. ^{1}H NMR spectrum of 10 (500 MHz, CDCl3)

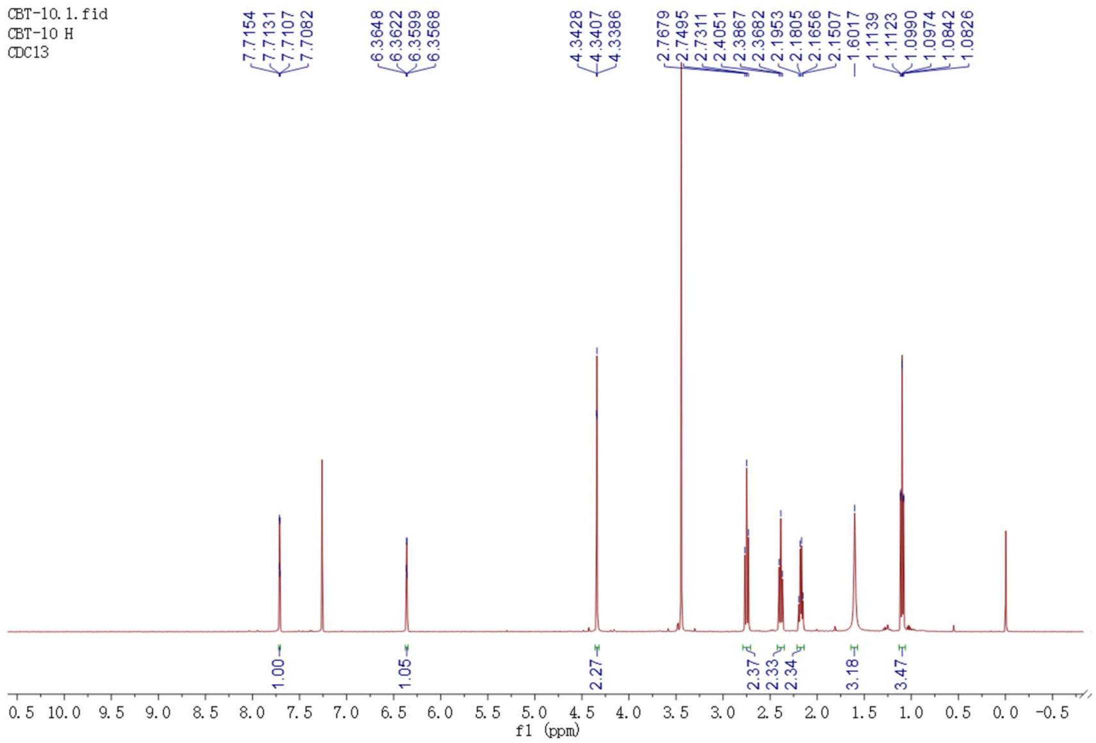

Fig. S64. ^{13}C NMR spectrum of 10 (125 MHz, CDCl3)

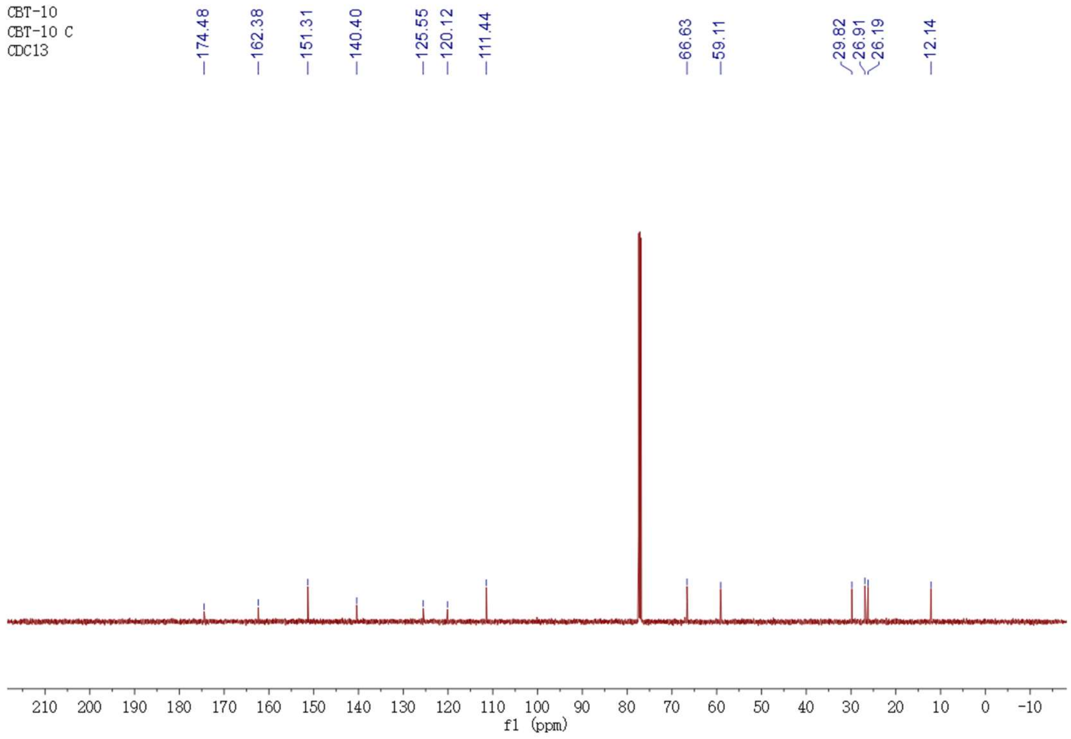

Fig. S65. HSQC spectrum of 10

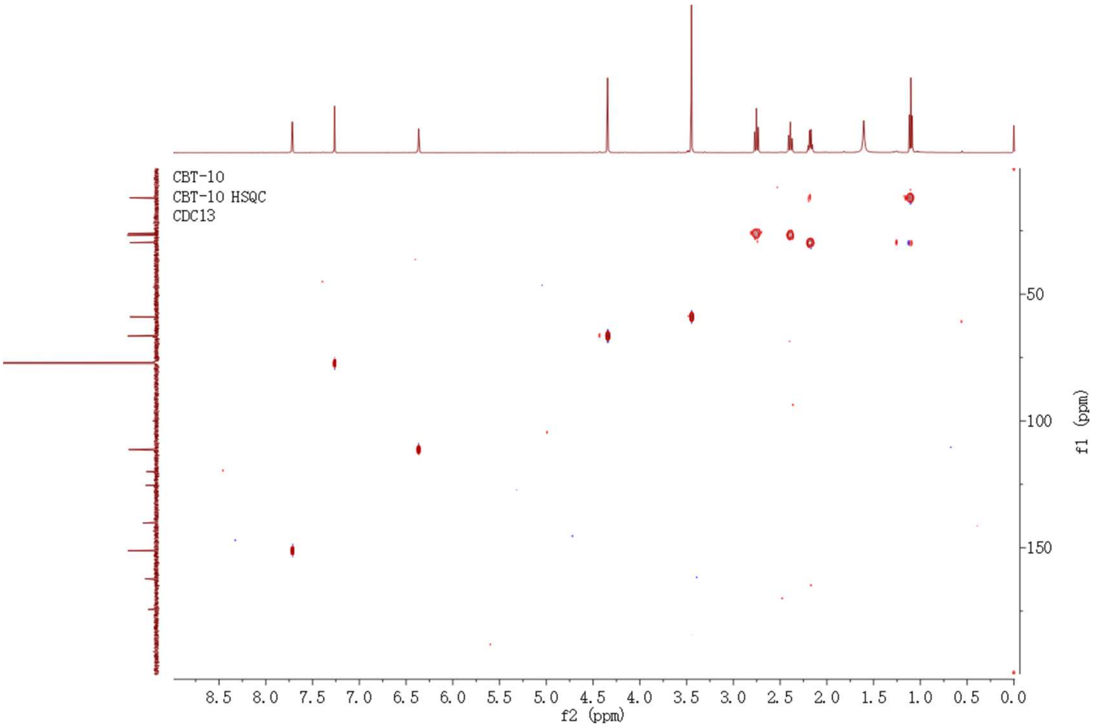

Fig. S66. HMBC spectrum of 10

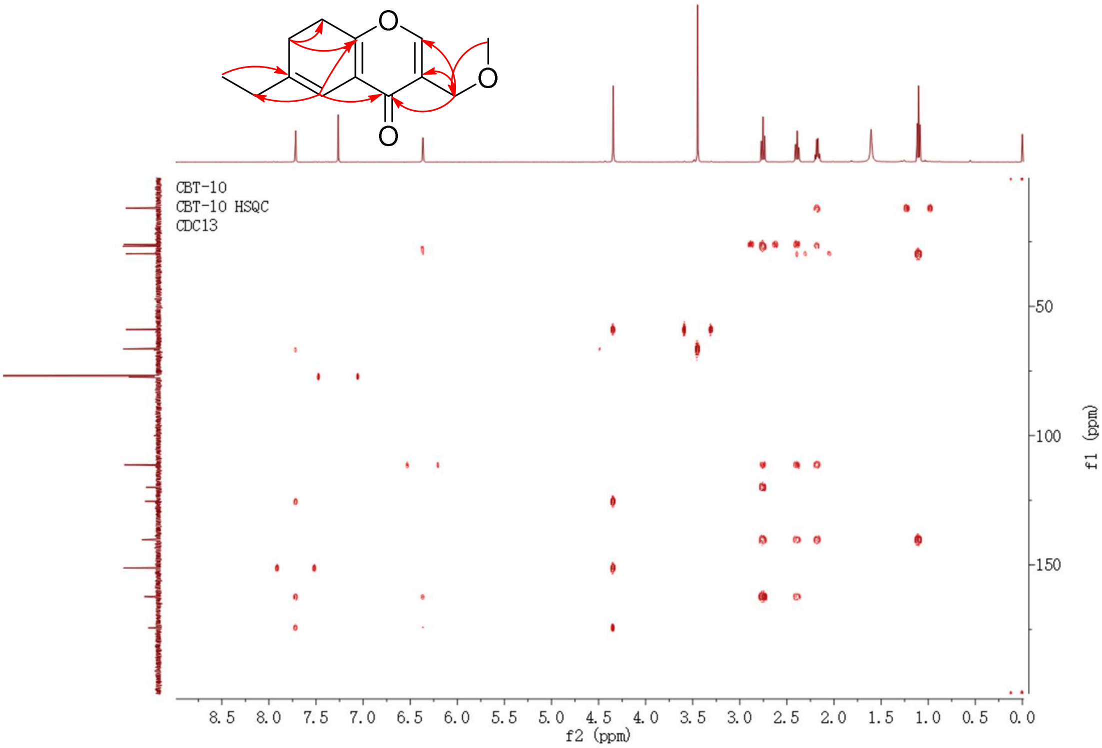

Fig. S67. ^{1}H NMR spectrum of 11 (500 MHz, CDCl$_3$)

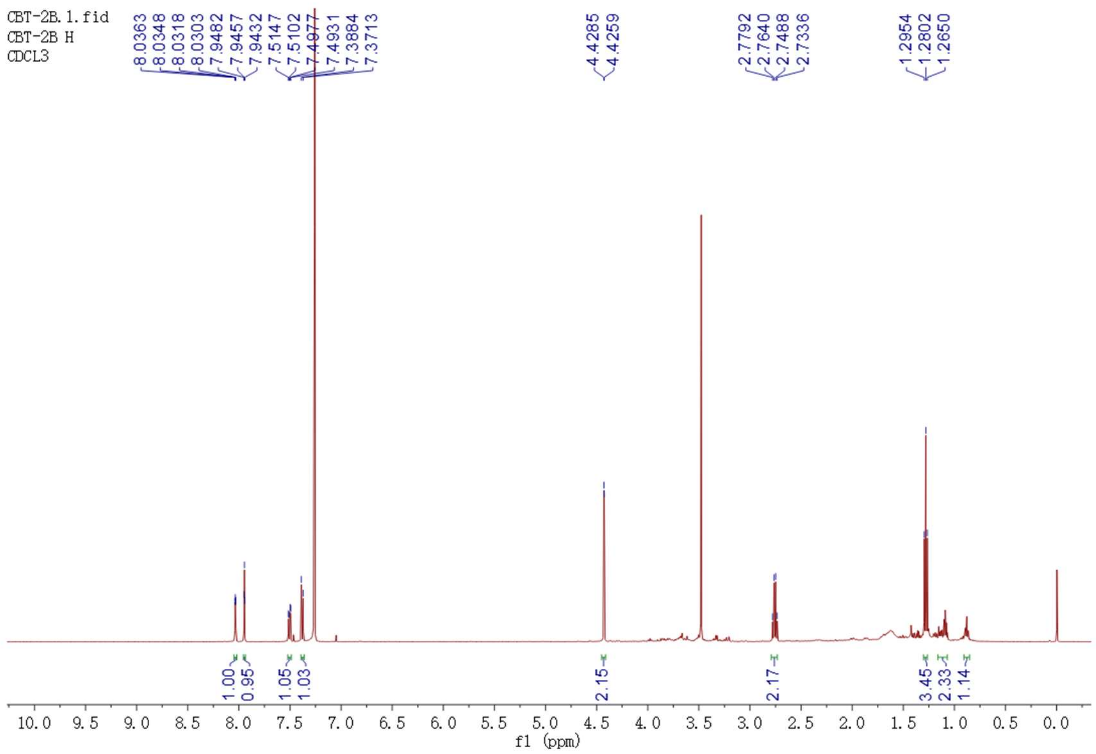

Fig. S68. ^{13}C NMR spectra of 11 (125 MHz, CDCl$_3$)

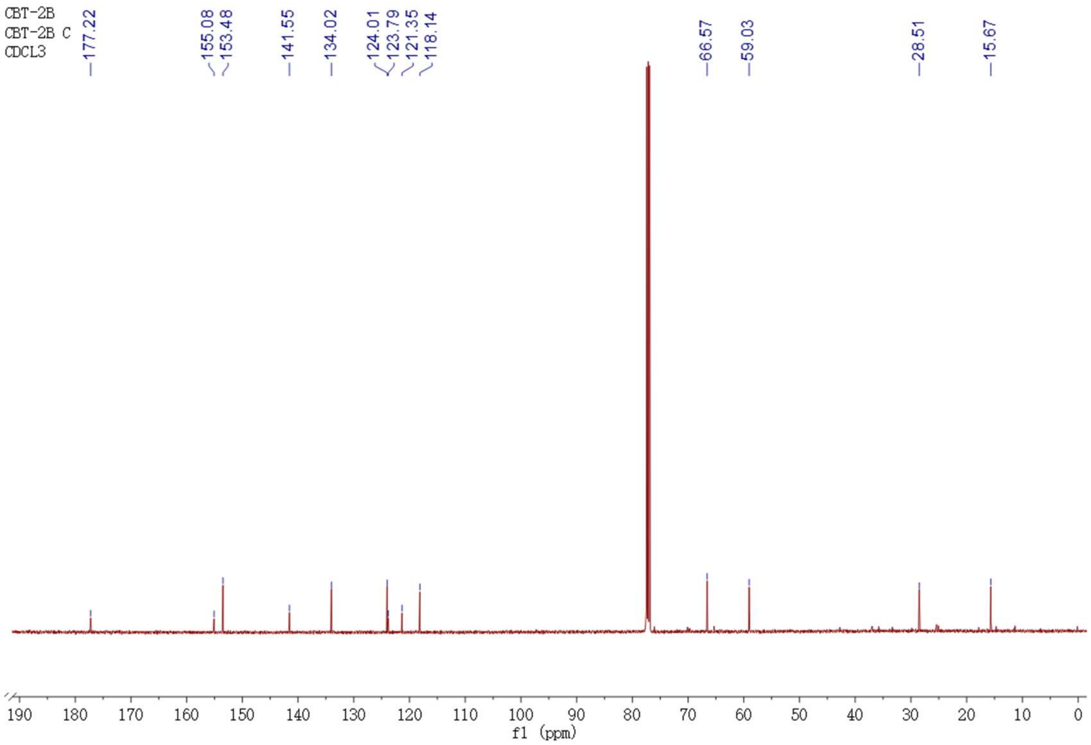

Fig. S69. HSQC spectrum of 11

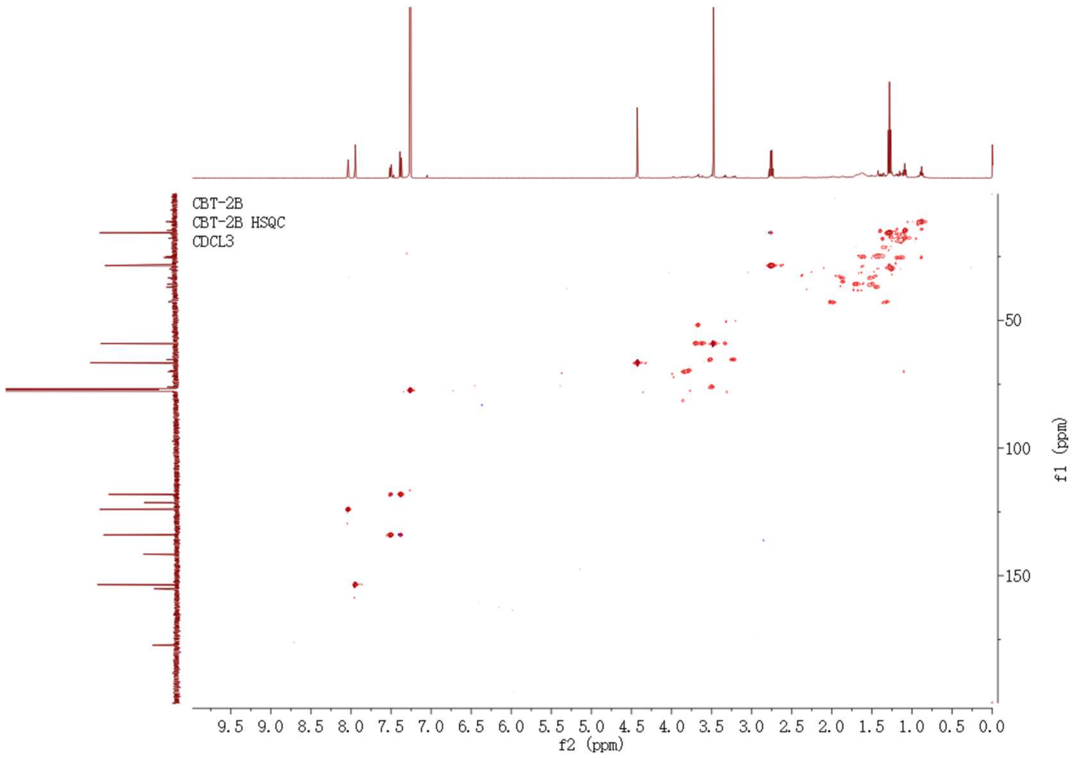

Fig. S70. HMBC spectrum of 11

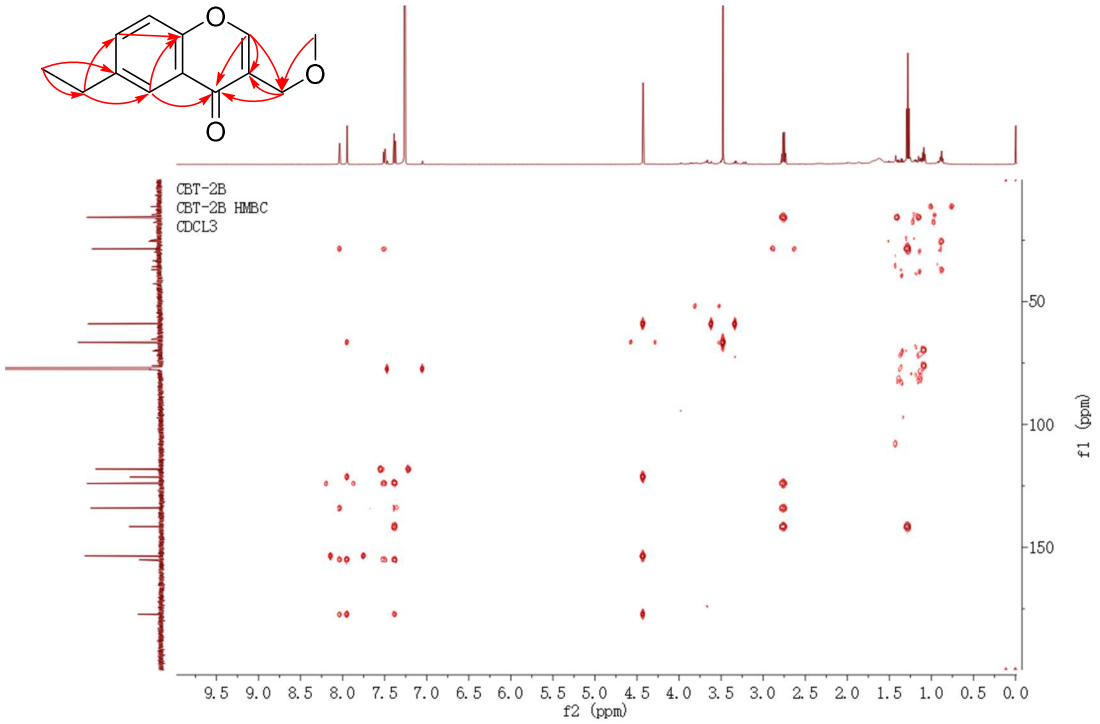

Fig. S71. HRESIMS report of 11

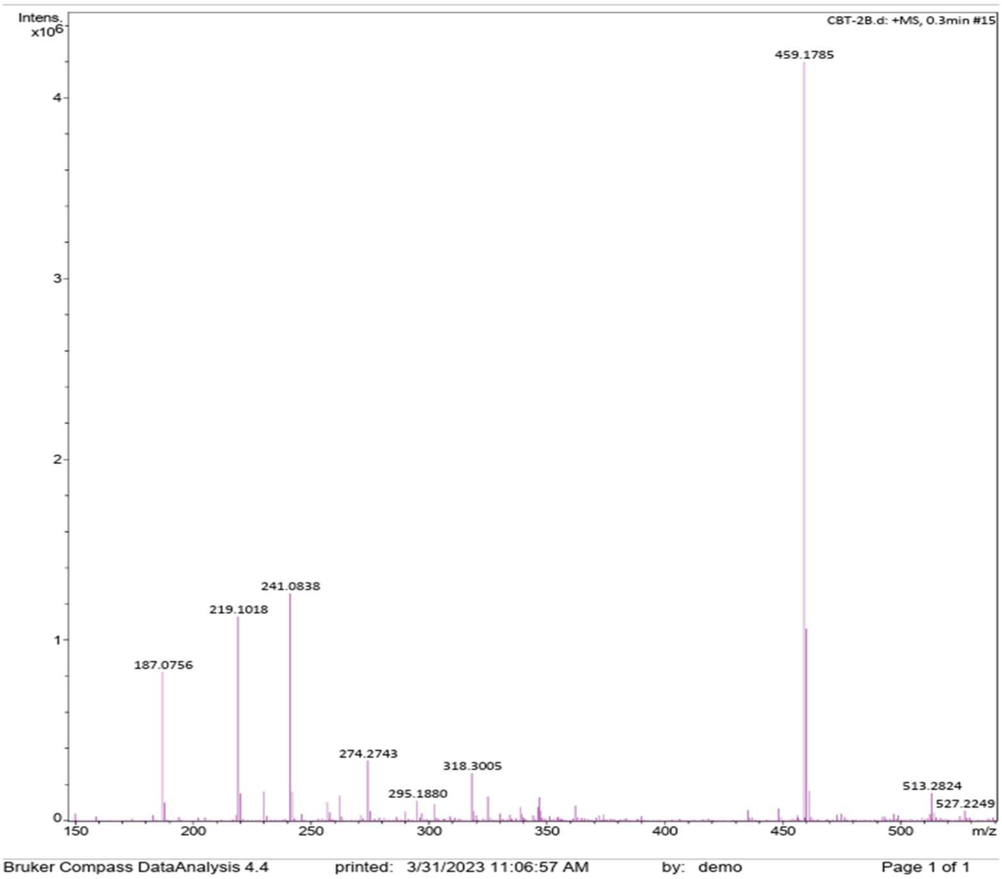

Fig. S72. ^{1}H NMR spectrum of 12 (500 MHz, $CDCl_3$)

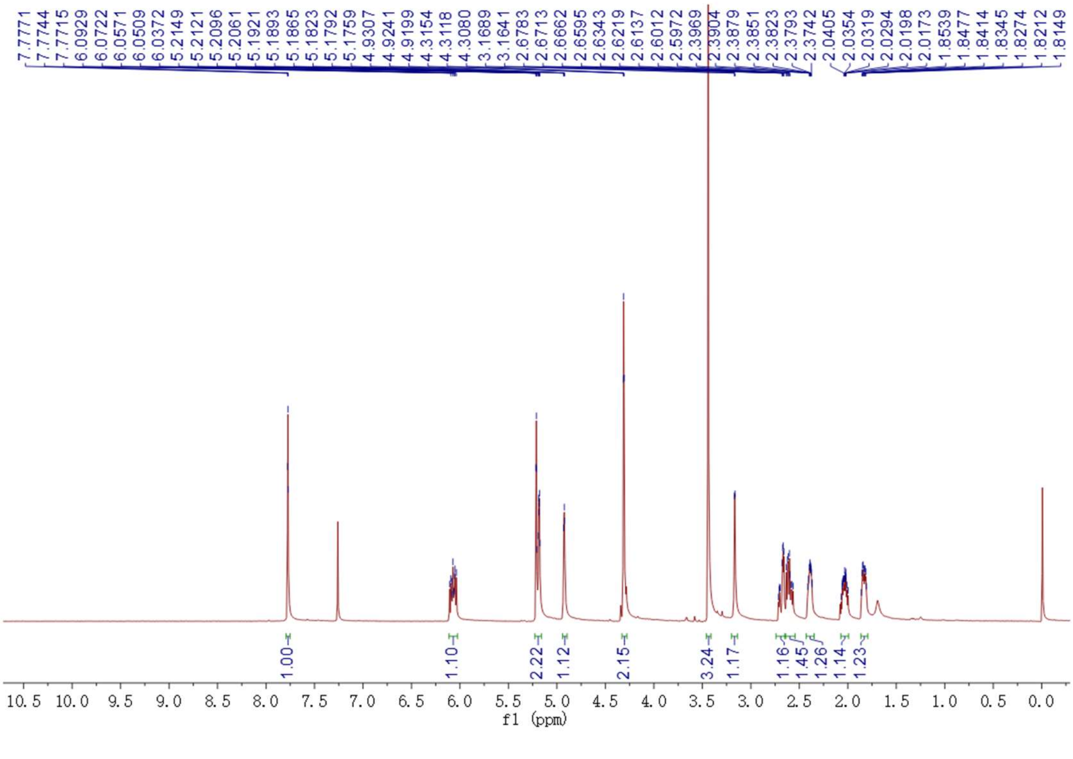

Fig. S73. ^{13}C NMR spectrum of 12 (125 MHz, $CDCl_3$)

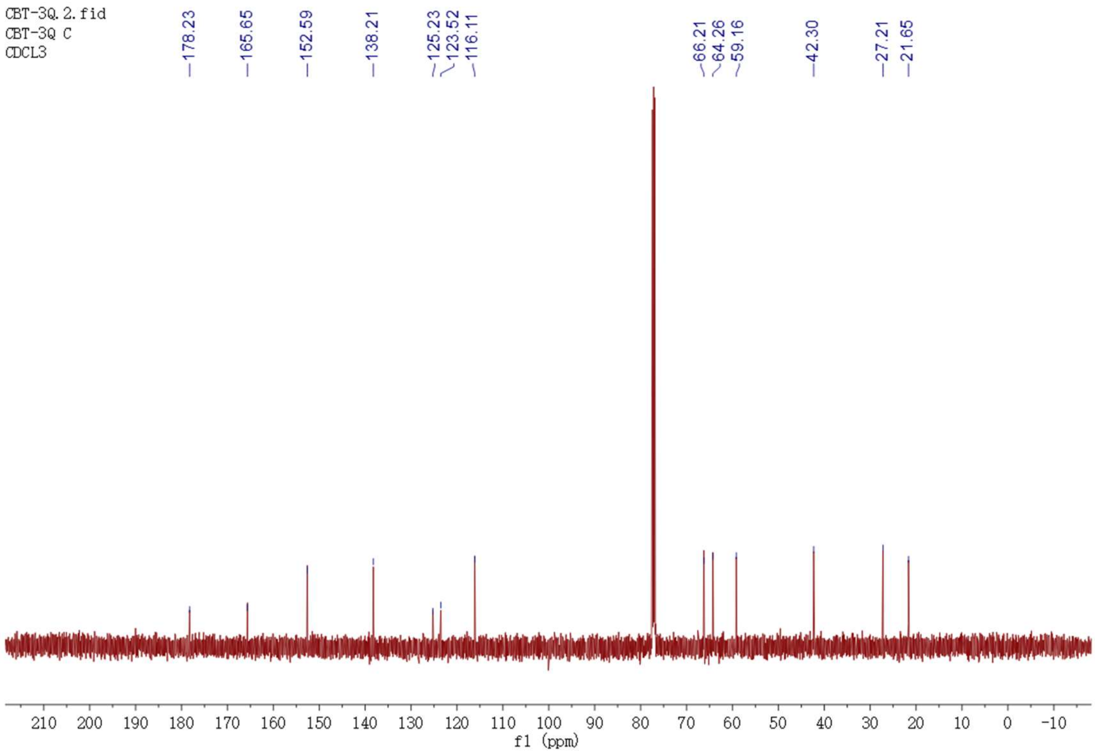

Fig. S74. HSQC spectrum of 12

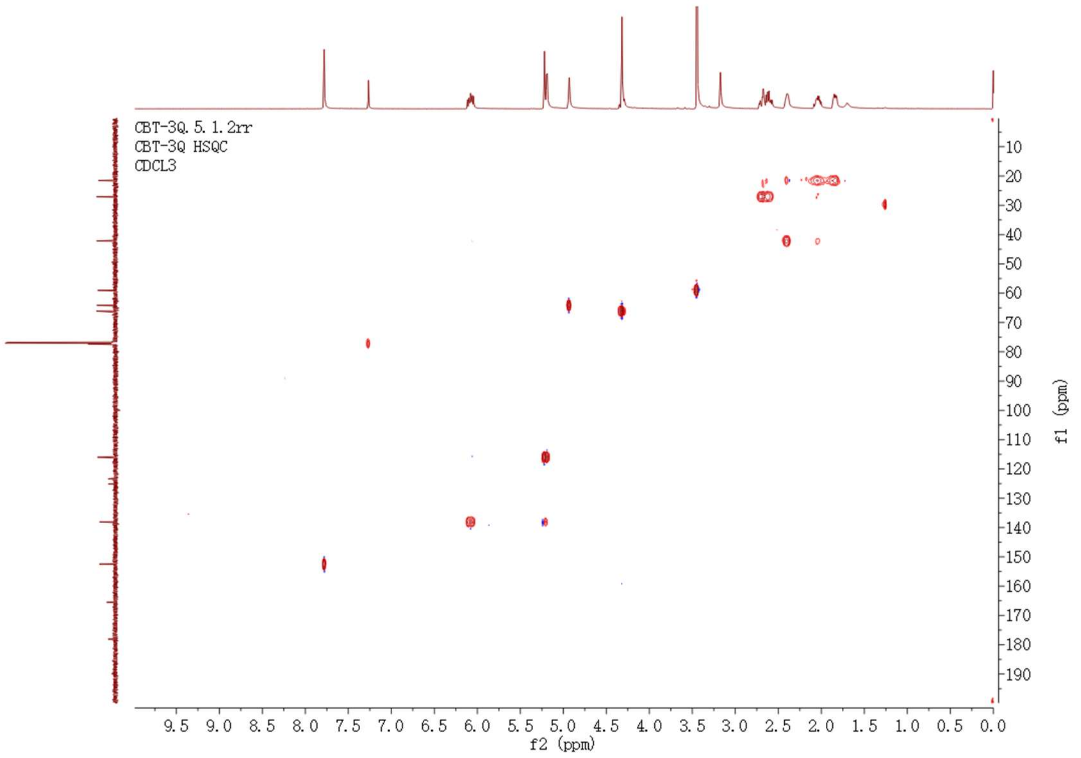

Fig. S75. ^{1}H-^{1}H COSY spectrum of 12

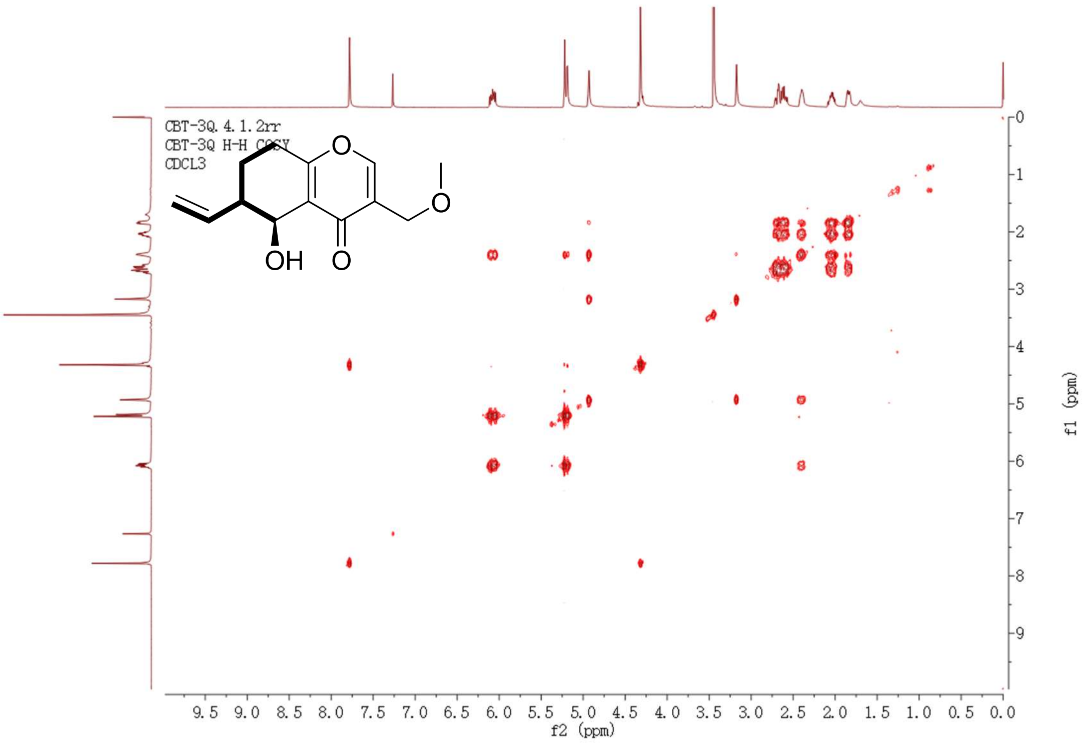

Fig. S76. HMBC spectrum of 12

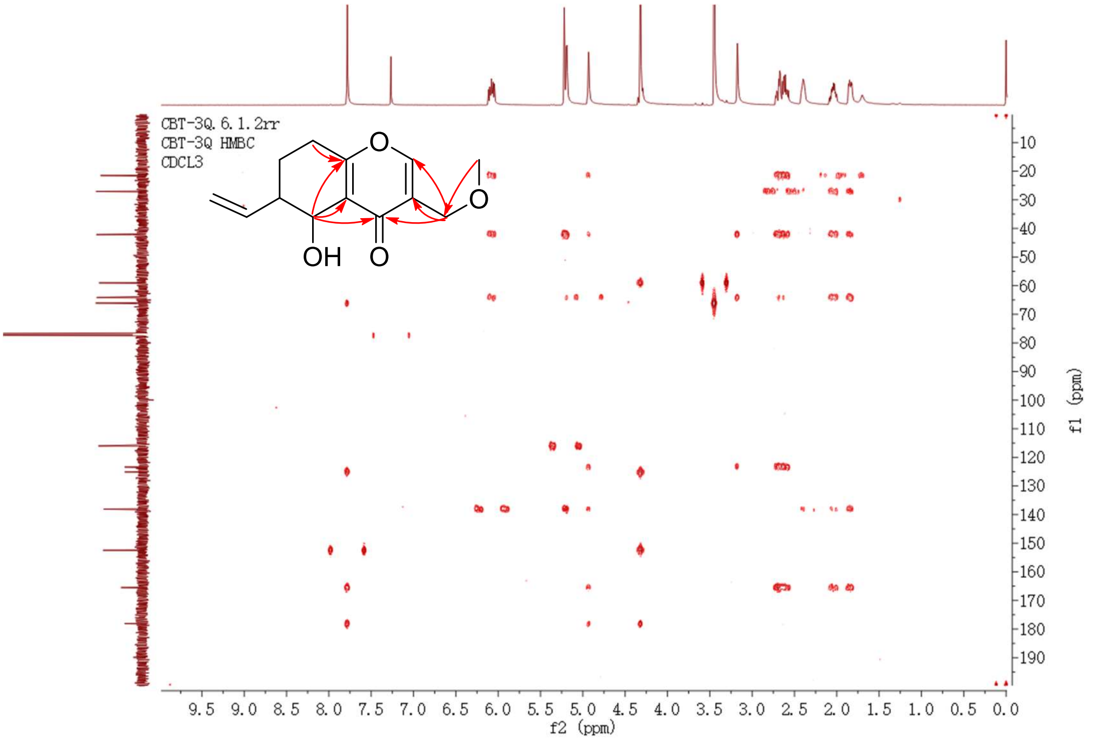

Fig. S77. ROESY spectrum of 12

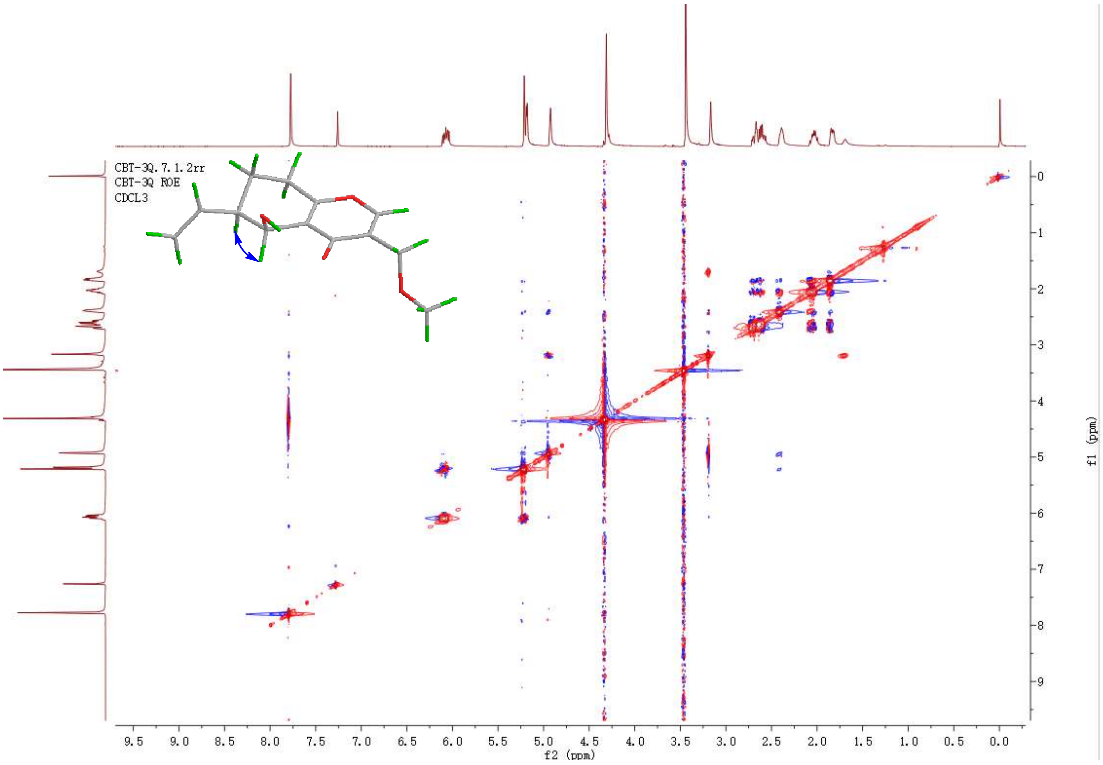

Fig. S78. HRESIMS report of 12

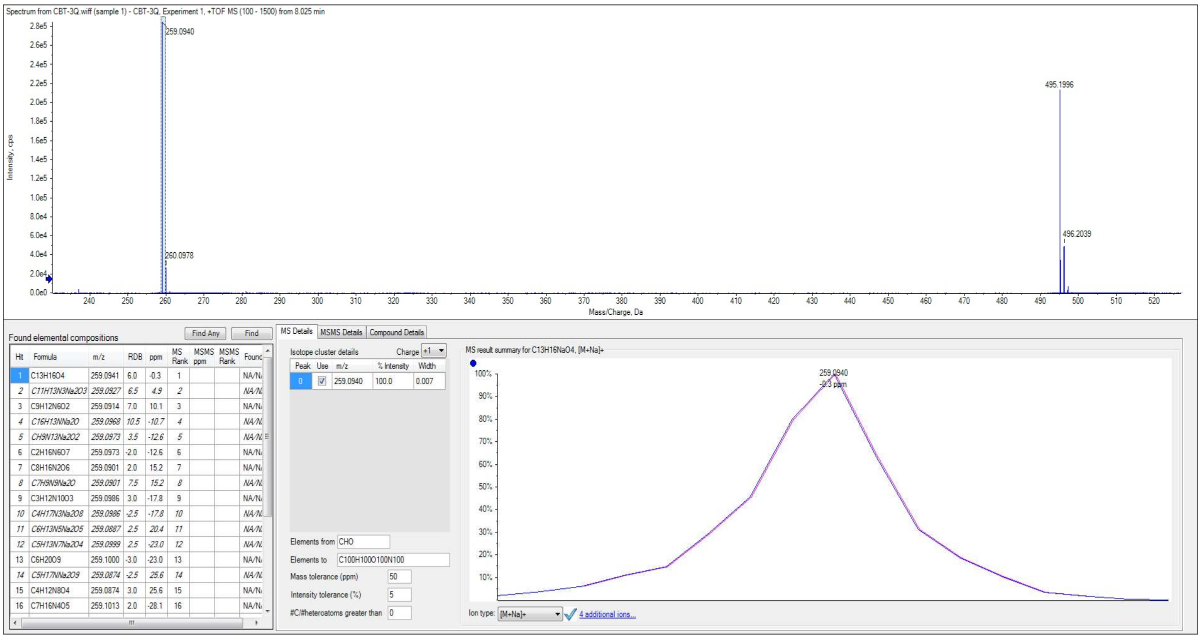

Fig. S79. ^{1}H NMR spectrum of 13 (500 MHz, $CDCl_3$)

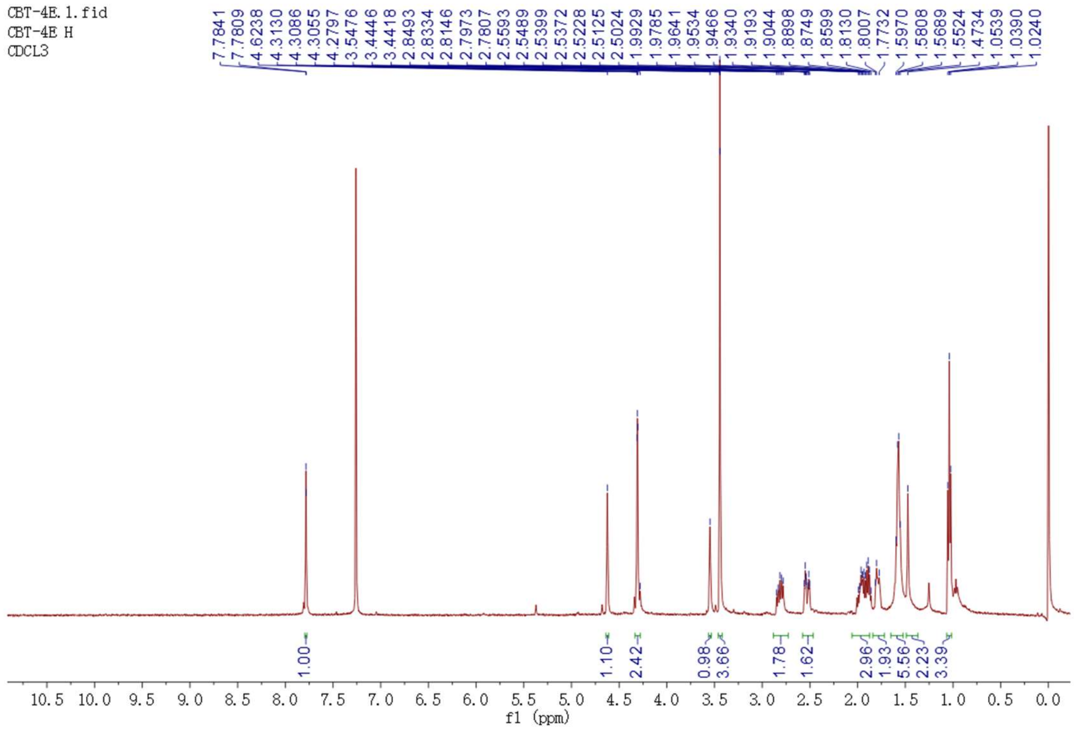

Fig. S80. ^{13}C NMR spectrum of 13 (125 MHz, $CDCl_3$)

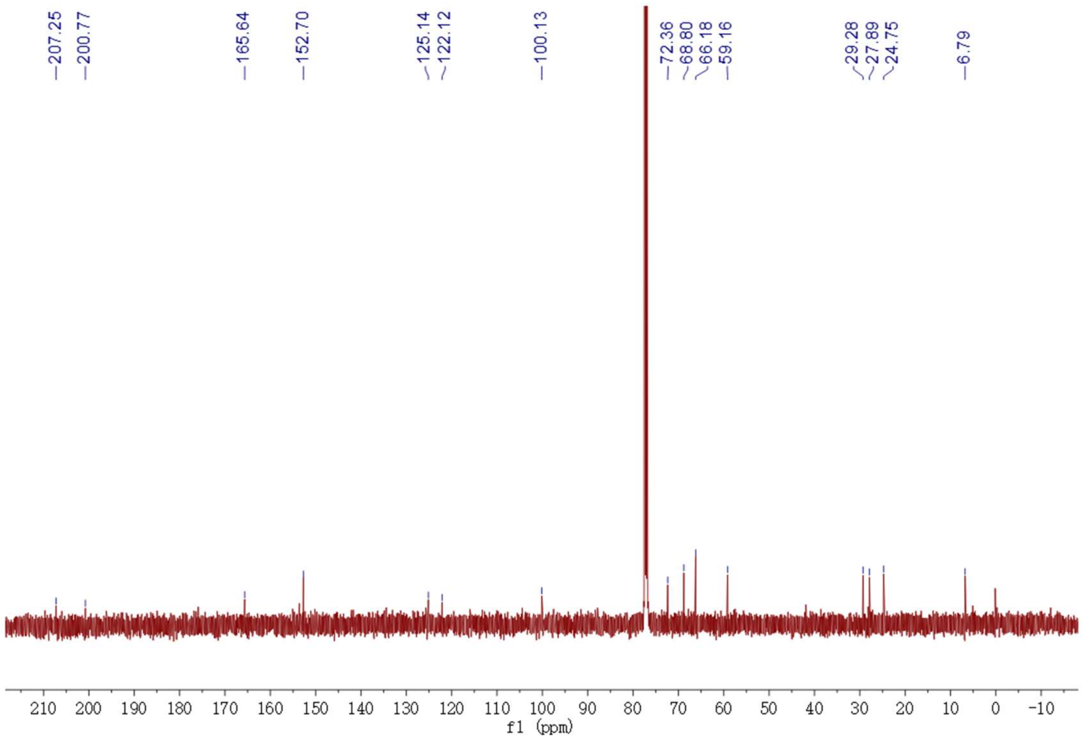

Fig. S81. HSQC spectrum of 13

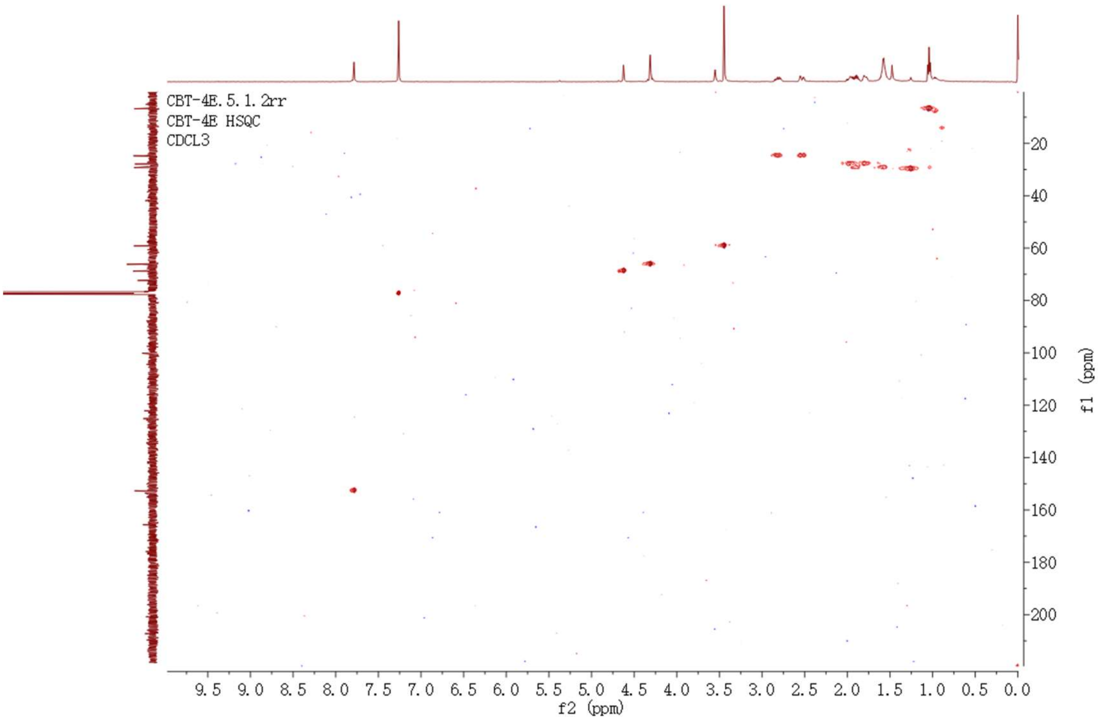

Fig. S82. HMBC spectrum of 13

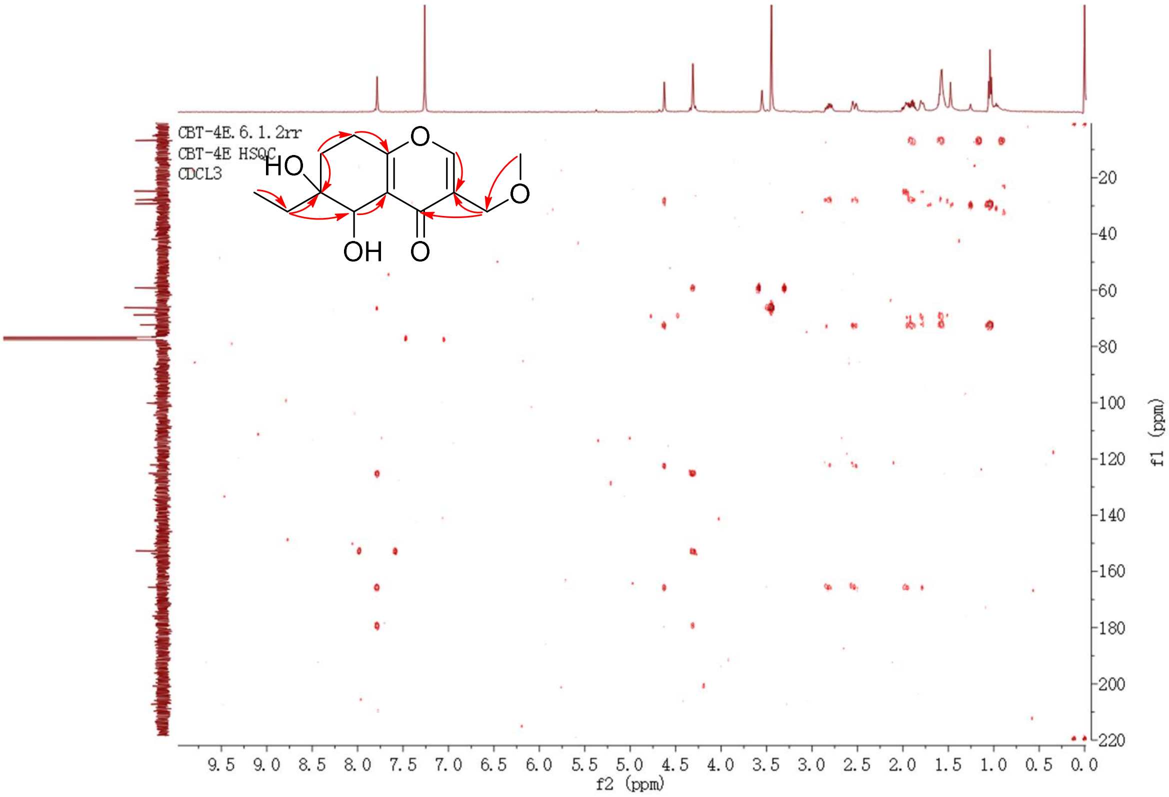

Fig. S83. ROESY spectrum of 13

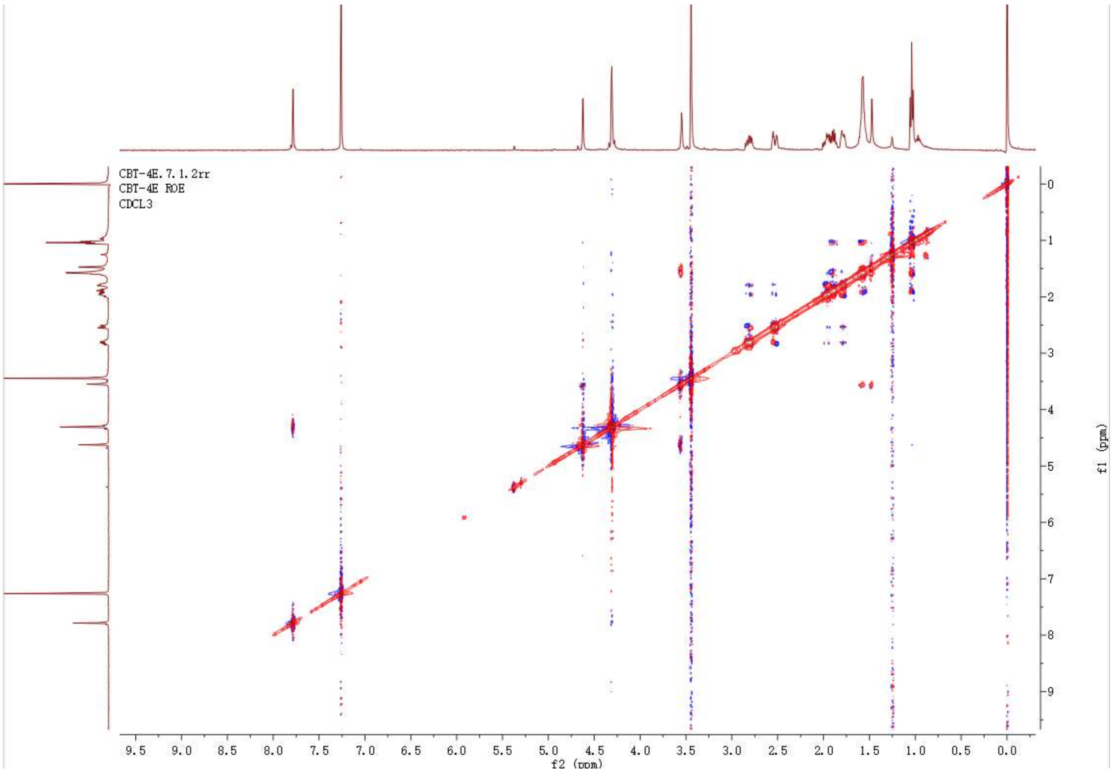

Fig. S84. HRESIMS report of 13

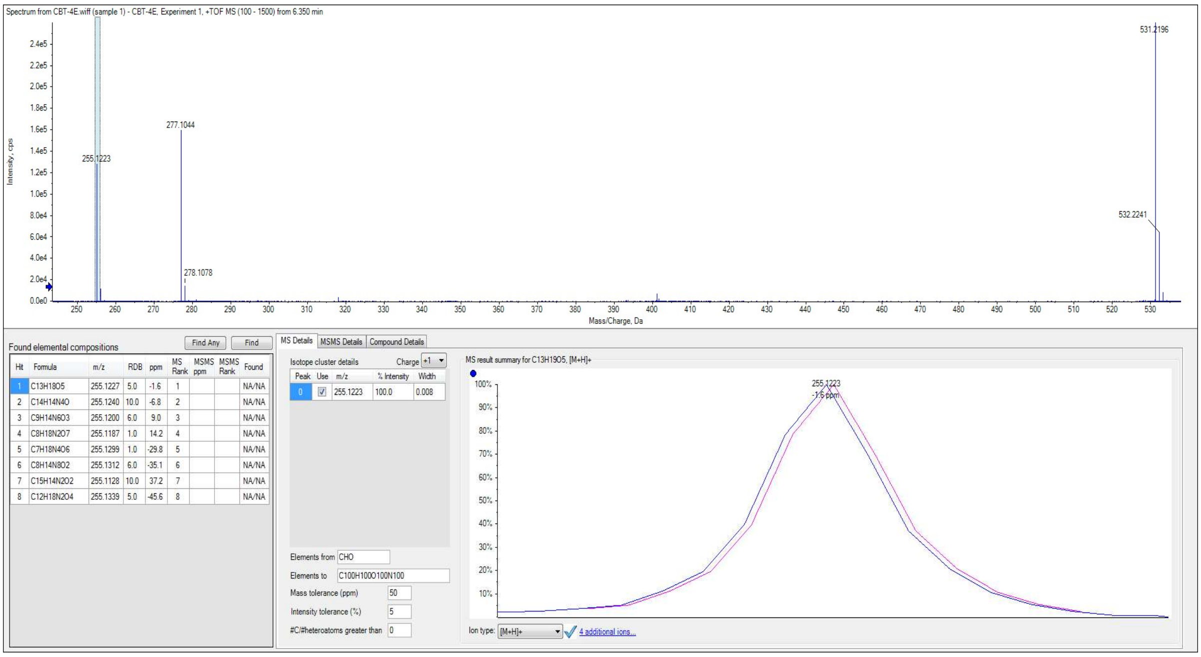

Fig. S85. ^{1}H NMR spectrum of 14 (500 MHz, CDCl₃)

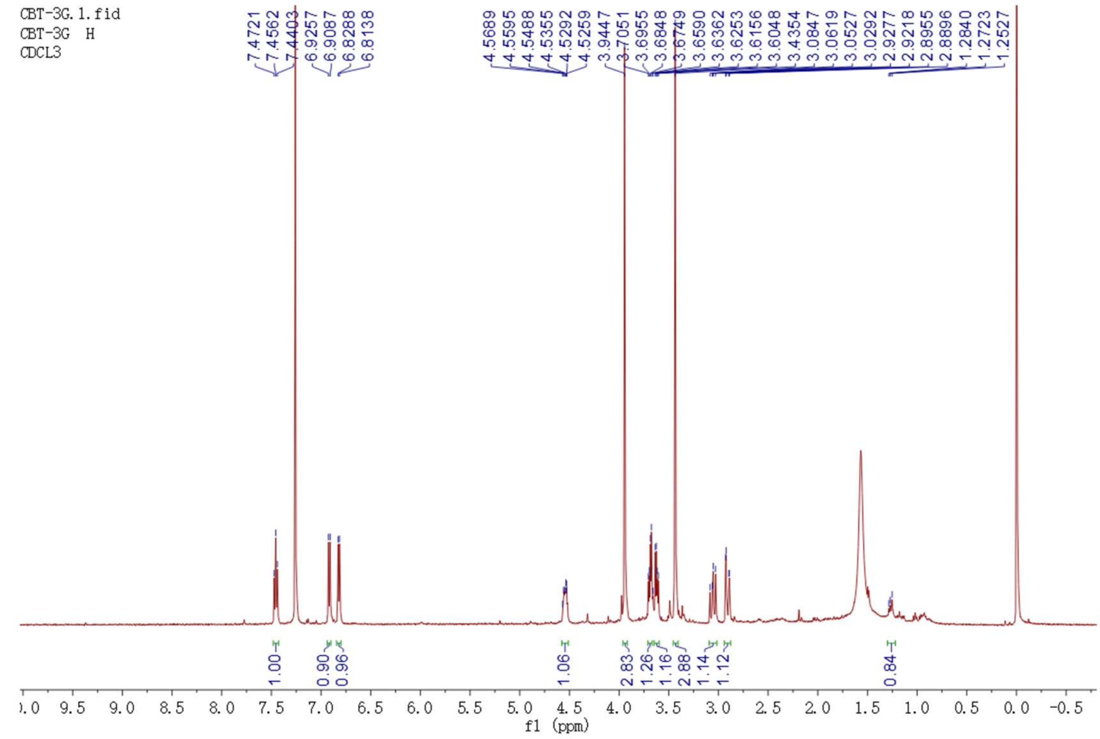

Fig. S86. ^{13}C NMR spectrum of 14 (125 MHz, CDCl₃)

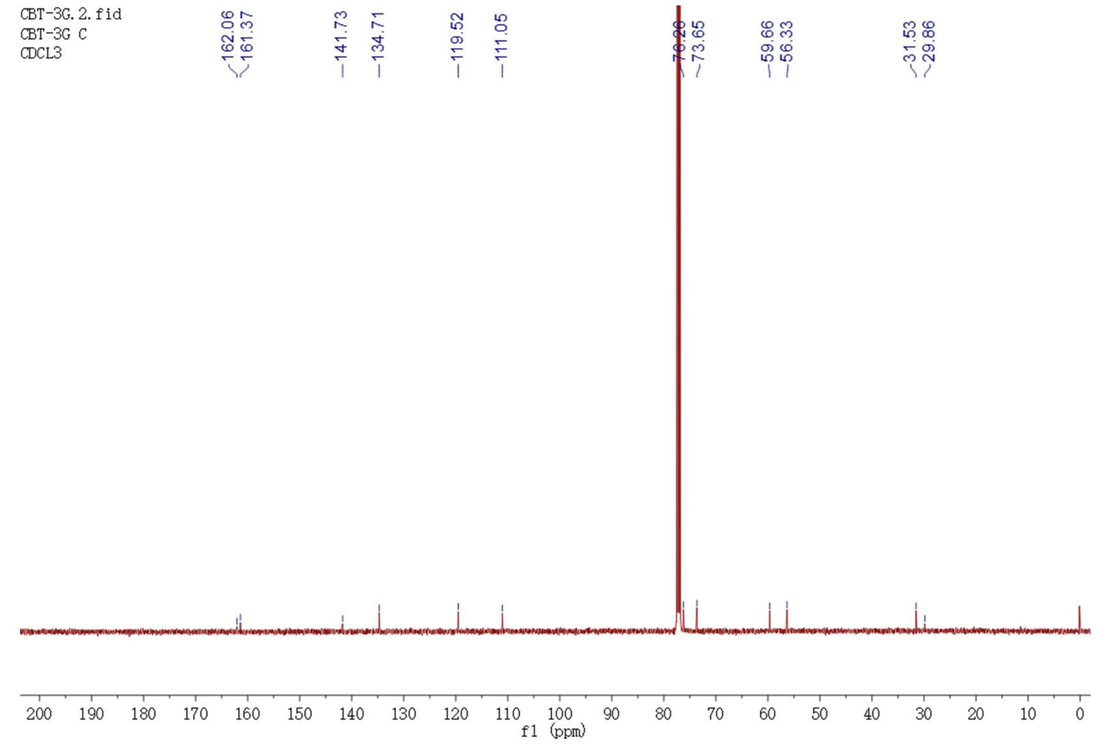

Fig. S87. HSQC spectrum of 14

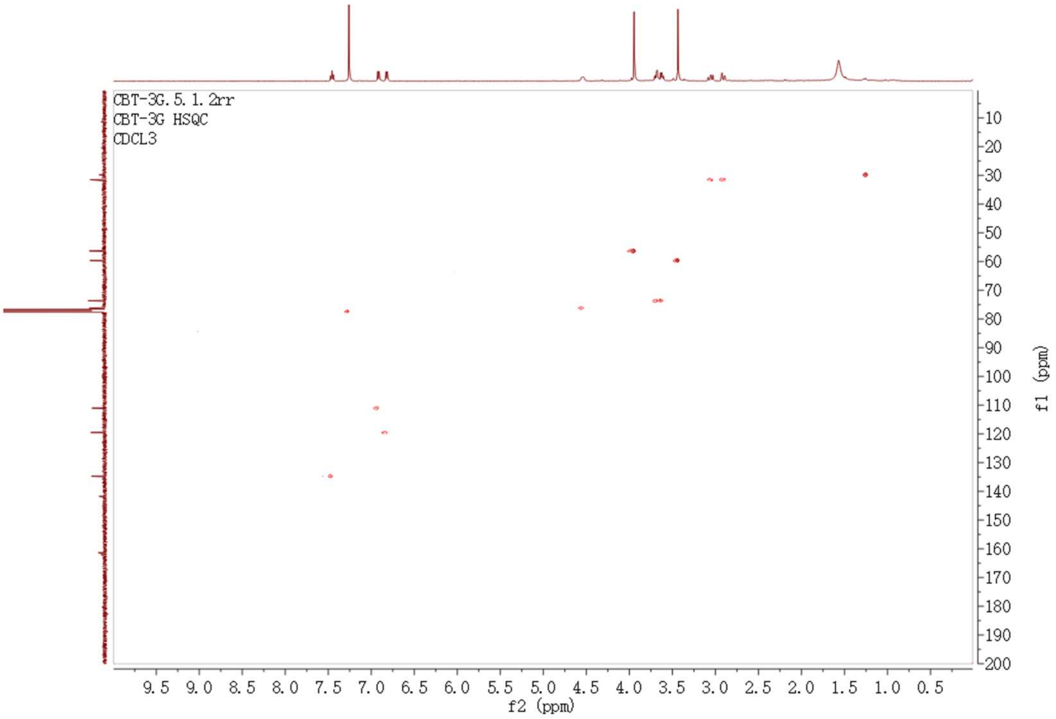

Fig. S88. HMBC spectrum of 14

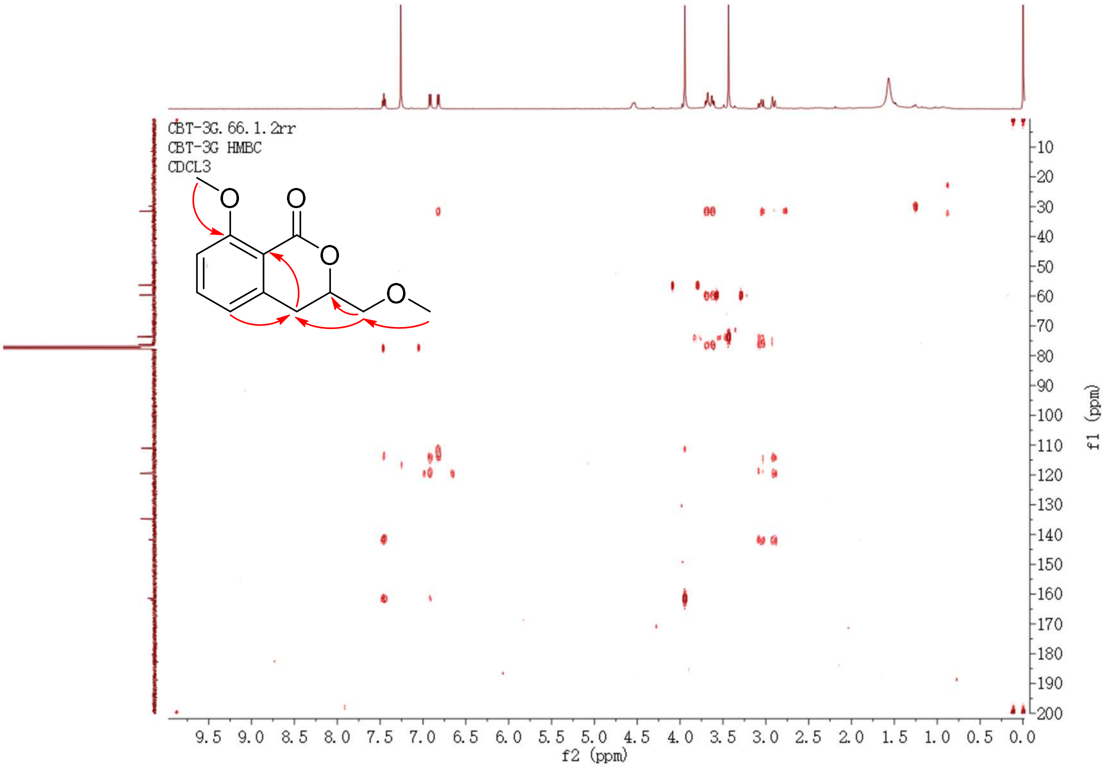

Fig. S89. HRESIMS report of 14

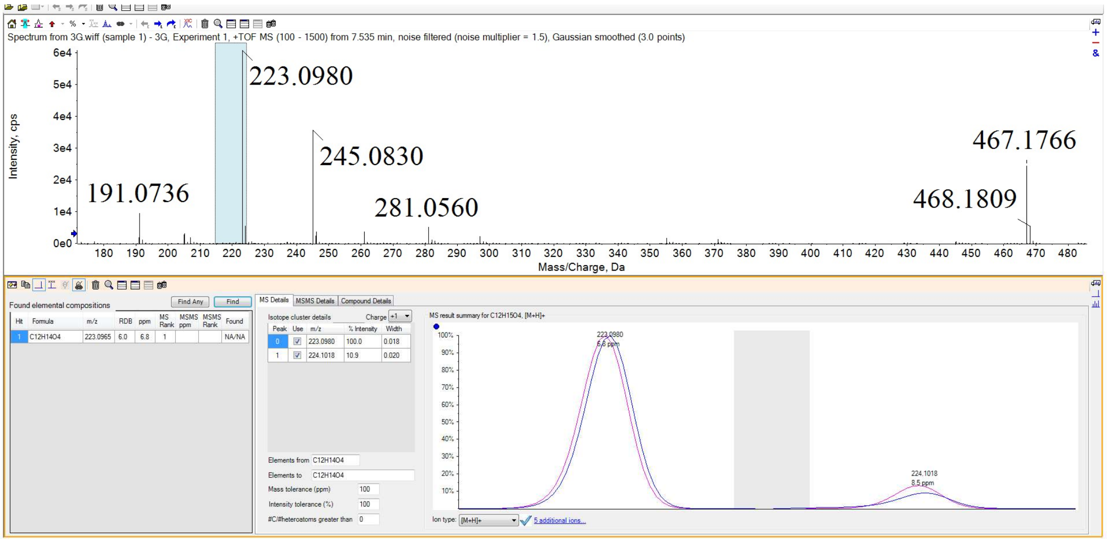

Fig. S90. Comparison of chemical shifts of pyranoses in compounds 2, 16, 3, and 17

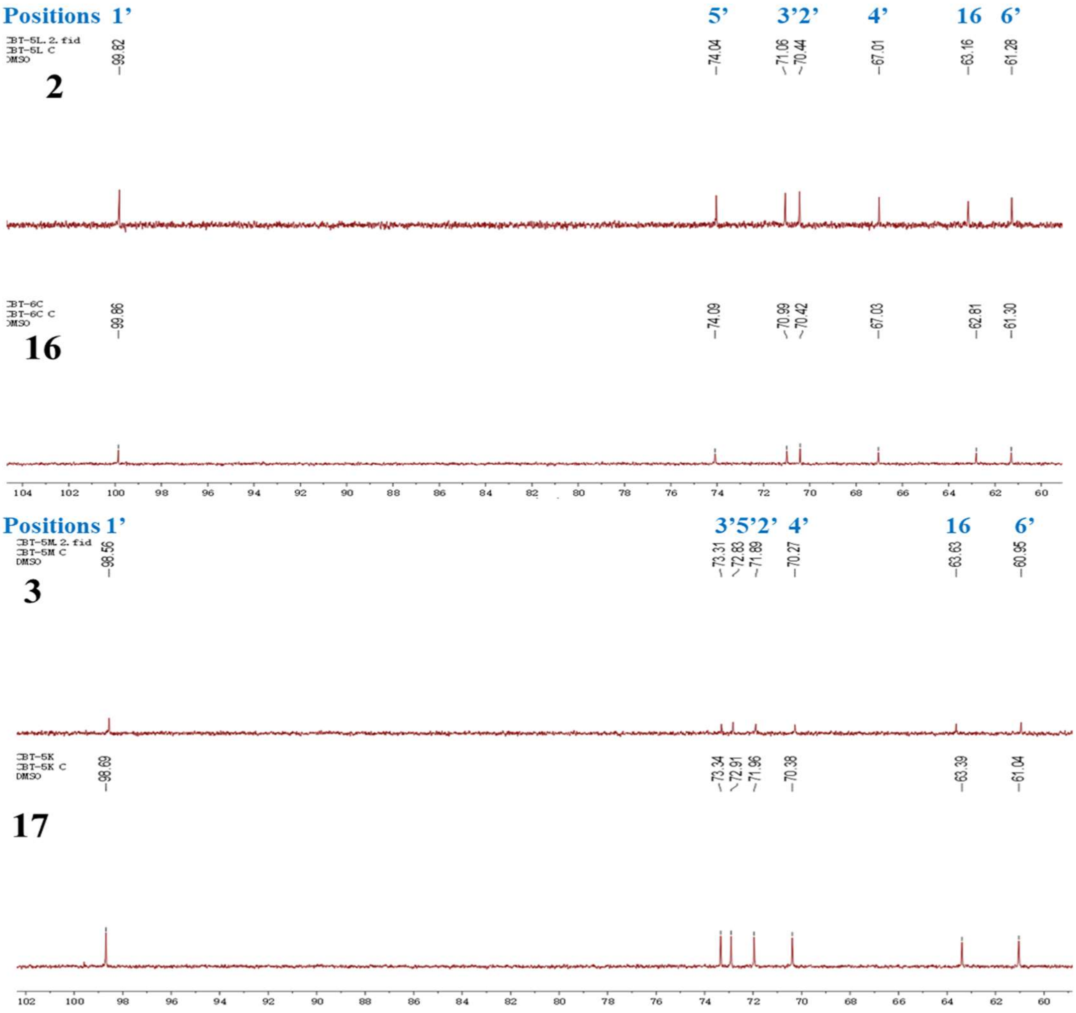

3. Comparison of 1D NMR data between 4 and 18

Table S2. ^{1}H (500 MHz) and ^{13}C NMR (125 MHz) data of **4** and **18** (cyclo(*N*-methyl-*L*-Phe-*L*-Val-*D*-Ile-*L*-Leu-*L*-Pro) (DMSO-d_6, *J* in Hz).

Amino acid	pos.	**4**		**18**	
		δ_C	δ_H	δ_C	δ_H
**4**: *L*–	CO	170.5		170.9	
phenylalanin	*α*	54.2	4.37, dd (15.5, 7.9)	56.5	5.07 (m)
e	*β*	36.6	2.84, m	34.4	3.02, m
**18**: *L*-N-					2.89, dd (14.2, 5.7)
methyl	*γ*	137.5		137.8	
phenylalanin	*ortho*	129.2	7.17, m	129.0	7.20, m
e	*meta*	128.0	7.24, m	128.6	7.24, m
	para	126.2	7.24, m	126.8	7.17, m
	NH		8.42, d (7.9)		
	N-CH_3			30.6	3.05, s
L–valine	CO	171.4		171.5	
	α	58.2	3.83, t (9.2)	58.1	3.95, t (9.8)
	β	26.1	1.94, m	26.9	1.98, m
	γ	19.7	0.80, d (5.0);	18.9	0.81, d (4.6);
		18.4	0.62, d (6.6)		0.71, d (6.8)
	NH		8.35, d (9.2)	20.2	8.12, d (9.5)
D–isoleucine	CO	170.2		170.8	
	α	55.7	4.19, dd (8.5, 7.1)	55.7	4.23, m
	β	37.8	1.52, m	38.1	1.53, m
	γ	25.6	1.27, m; 1.01, m	26.4	1.26, m; 1.00, m
	σ	11.5	0.83, d (6.2)	11.9	0.83, d (7.5)
	β–CH_3	14.4	0.72, d (6.9)	14.7	0.69, d (6.8)
	NH		7.07, d (8.5)		6.92, d (8.9)
L–leucine	CO	168.8		169.4	
	α	47.6	4.61, dd (14.5, 8.6)	47.1	4.70, m
	β	41.5	1.51, m; 1.41, m	41.7	1.40, m
	γ	24.4	1.35, m	24.6	1.40, m
	σ	22.7	0.85, d (5.4)	23.3	0.81, d (5.4)
		22.4	0.82, d (5.3)	22.7	0.78, d (4.6)
	NH		8.34, d (8.6)		8.43, d (9.4)
L–proline	CO	170.4		172.5	
	α	59.9	4.67, d (7.8)	59.2	5.07, m
	β	32.0	1.94, m; 1.61, m	30.6	1.84, m; 0.98, m
	γ	20.9	1.65, m; 1.41, m	20.9	1.63, m; 1.30, m
	σ	45.6	3.28, m	46.2	3.28, m

4. Computational details

4.1 Computational details for compound 1

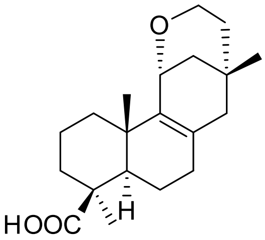

Fig. S91. Chemical structure of **1**.

A conformation search based on molecular mechanics with MMFF94s force fields was performed for **1**, giving two conformers with distributions higher than 1% (Table S3).[1,2] All these conformers were further optimized by the density functional theory method at the B3LYP/6-31G(d,p) level in the Gaussian 09 program package,[3] leading to two conformers within 3 kcal/mol energy threshold from the global minimum, respectively (Table S3) (**1a** and **1b**). The predominant conformers were subjected to theoretical calculation of ECD using time-dependent density functional theory (TDDFT) at B3LYP/6-31G(d,p) level with CPCM model in methanol based on B3LYP/6-31G(d,p) optimized conformers. The calculated ECD curves for **1** were weighted using SpecDis 1.71 with $\sigma = 0.22$ eV, and UV shift 5 nm, respectively.[4] The ECD curve of the enantiomer was generated by SpecDis by the function "enantiomeric ECD".

Conformers were subjected to theoretical calculation of NMR using GIAO B3LYP/6-31G(d) level in chloroform based on optimized conformers. The calculated ^{13}C NMR data were compared with experimental data using Excel (Table S4).

Table S3. Energy analysis for conformers of **1** at B3LYP/6-31G(d,p) level in the gas phase

Species	Gibbs free energy	ΔE (kcal/mol)	P_E%
**1a**	-1005.441422	-0.43	67.3
**1b**	-1005.440742	0.00	32.7

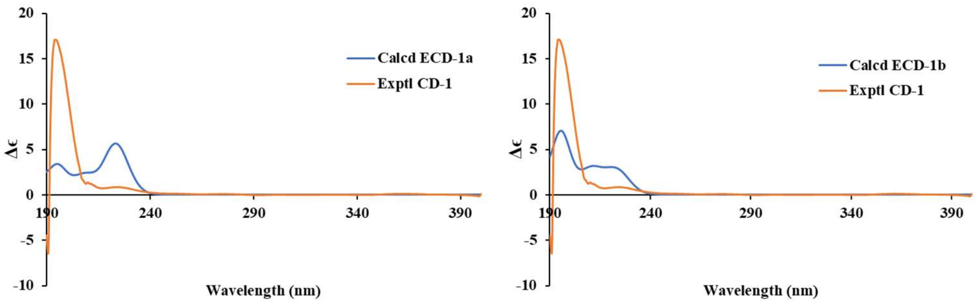

Fig. S92. Comparison of the calculated ECD spectra for **1a** ($\sigma = 0.22$ eV, and UV shift 5 nm) and **1b** ($\sigma = 0.22$ eV, and UV shift 5 nm) with its experimental CD spectrum in MeOH

Table S4. NMR calculation details of **1**

No.	Shielding Value	Exp	Corr	Δ
1	152.89	36.0	36.1	-0.1
2	168.76	19.3	19.2	0.1
3	151.18	37.8	37.9	-0.1
4	143.43	43.7	46.1	-2.4
5	136.95	53.3	53.0	0.3
6	167.53	20.5	20.5	0.0
7	156.78	32.0	31.9	0.1
8	57.23	134.7	137.6	-2.9
9	58.92	133.3	135.8	-2.5
10	148.49	38.2	40.7	-2.5
11	122.62	66.9	68.2	-1.3
12	150.31	40.1	38.8	1.3
13	159.81	27.6	28.7	-1.1
14	146.13	44.3	43.2	1.1
15	149.86	40.9	39.3	1.6
16	132.42	58.5	57.8	0.7
17	158.28	31.8	30.3	1.5
18	161.15	28.7	27.3	1.4
19	21.66	181.2	175.3	5.9
20	170.7	18.2	17.2	1.0
MAE				2.0

4.2 Computational details for compound 3

A conformational search based on molecular mechanics using MMFF94s force fields was conducted for **3**, resulting in five conformers with distributions exceeding 1%. All of these conformers underwent further optimization via density functional theory at the B3LYP/6-31G(d,p) level in the Gaussian 16 software package, yielding two conformers within a 3 kcal/mol energy threshold from the global minimum (Table S5), respectively. The predominant conformers were subjected to theoretical ECD calculations using TDDFT at the B3LYP/6-31G(d,p) level with the CPCM model in methanol based on the B3LYP/6-31G(d,p) optimized conformers. The calculated ECD curve for **3** was weighted using SpecDis 1.71.

Table S5. Energy analysis for conformers of **3** at B3LYP/6-31G(d,p) level in the gas phase

Species	Gibbs free energy	ΔE (kcal/mol)	P_E%
**3-1**	-1617.316036	0.50	30.2
**3-2**	-1617.316825	0.00	69.8

4.3 Computational details for compounds 5 and 6

A conformational search based on molecular mechanics using MMFF94s force fields was conducted for **5a** and **6a**, resulting in three and six stable conformers with distributions exceeding 1%. All of these conformers underwent further optimization via density functional theory at the B3LYP/6-31G(d,p) level in the Gaussian 16 software package, yielding conformers three and six within a 3 kcal/mol energy threshold from the global minimum (Tables S6 and S7), respectively. The predominant conformers were subjected to theoretical ECD calculations using TDDFT at the

B3LYP/6-31G(d,p) level with the CPCM model in methanol, based on the B3LYP/6-31G(d,p) optimized conformers. The calculated ECD curves for **5a** and **6a** were weighted using SpecDis 1.71, respectively. The ECD curve for the enantiomers **5b** and **6b** was generated by SpecDis using the "enantiomeric ECD" function (Fig. S94).

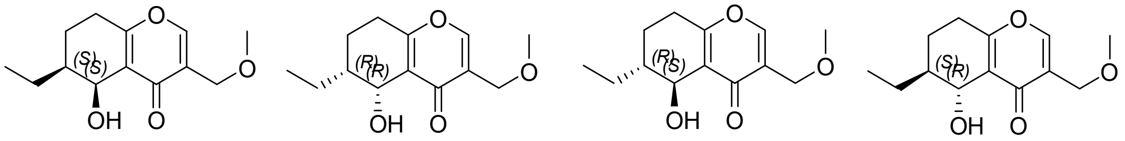

5a **5b** **6a** **6b**

Fig. S93. Possible structures of **5** and **6**.

Table S6. Energy analysis for conformers of **5a** at B3LYP/6-31G(d,p) level in the gas phase

Species	Gibbs free energy	*ΔE* (kcal/mol)	P_E%
**5a-1**	-806.839998	-0.35	58.2
**5a-2**	-806.839447	0.00	32.4
**5a-3**	-806.838278	0.73	9.4

Table S7. Energy analysis for conformers of **6a** at B3LYP/6-31G(d,p) level in the gas phase

Species	Gibbs free energy	*ΔE* (kcal/mol)	P_E%
**6a-1**	-806.841834	0.00	62.6
**6a-2**	-806.840864	0.61	22.4
**6a-3**	-806.837768	2.55	0.8
**6a-4**	-806.840294	0.97	12.3
**6a-5**	-806.837560	2.68	0.7
**6a-6**	-806.838084	2.35	1.2

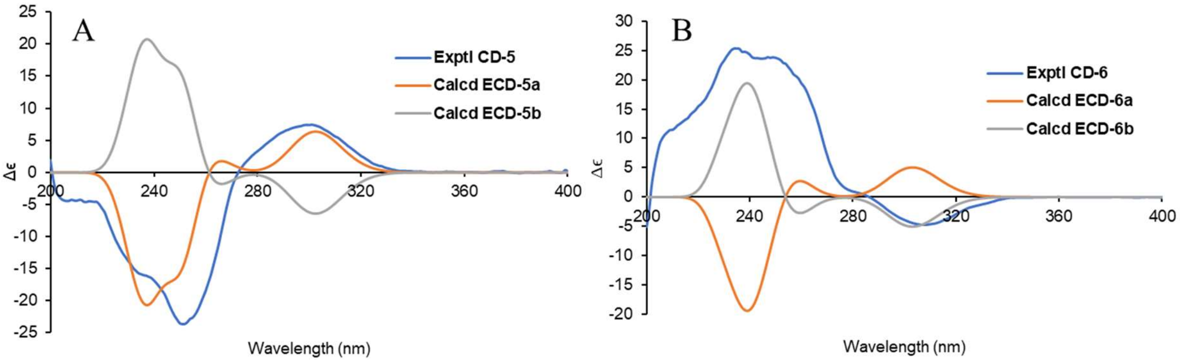

Fig. S94. Comparison of the calculated ECD spectra with experimental ones in MeOH; (A) **5** (σ = 0.20 eV, UV shift 7 nm); (B) **6** (σ = 0.22 eV, UV shift 9 nm).

4.4 Computational details for 12

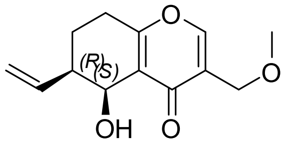

Fig. S95. Chemical structure of **12**.

A conformation search based on molecular mechanics with MMFF94s force fields was performed for **12**, giving three stable conformers with higher than 1% distributions.[1,2] All these conformers were further optimized by the density functional theory method at the B3LYP/6-31G(d,p) level in

the Gaussian 16,[3] leading to two conformers within a 3 kcal/mol energy threshold from the global minimum (Table S8), respectively. The predominant conformers of **12** were subjected to theoretical calculation of ECD using TDDFT at B3LYP/6-31G(d,p) level with CPCM model in air based on B3LYP/6-31G(d,p) optimized conformers. The calculated ECD curve for **12** was weighted using SpecDis.[4] The ECD curve of the enantiomers was generated by SpecDis by the function "enantiomeric ECD".

Conformers of **12** were subjected to theoretical calculation of NMR using GIAO B3LYP/6-31G(d) level in chloroform based on optimized conformers. The calculated ^{13}C NMR data were compared with experimental data in Excel (Table S9).

Table S8. Energy analysis for conformers of **12** at B3LYP/6-31G(d,p) level in the gas phase.

Species	Gibbs free energy	ΔE (kcal/mol)	$P_E\%$
**12-1**	-805.652711	1.18	12.1
**12-2**	-805.654584	0.00	87.9

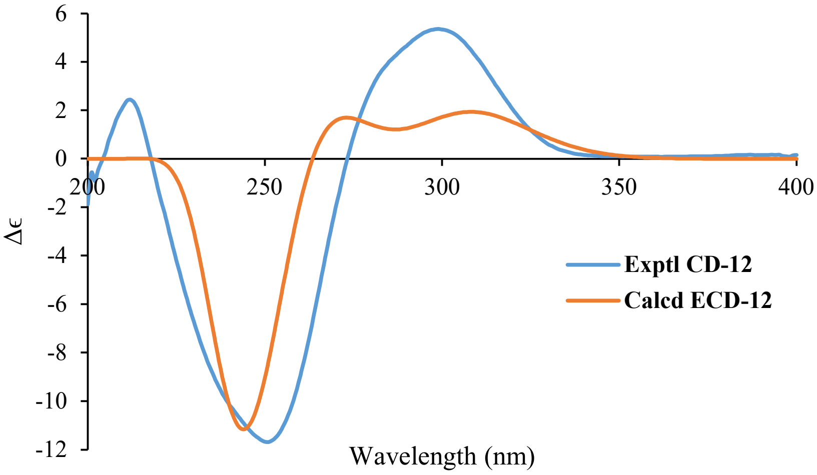

Fig. S96. Comparison of the calculated ECD spectrum for **12** (σ = 0.30 eV, UV shift 6 nm) with the experimental spectrum of **12** in MeOH.

Table S9. NMR calculation details of **12**.

No	Shielding value	Exp	Corr	Δ
2	44.21	152.6	151.7	0.9
3	66.33	125.2	127.8	-2.6
4	21.52	178.2	176.2	2.0
4a	67.47	123.5	126.6	-3.1
5	122.79	64.3	66.9	-2.6
6	144.39	42.3	43.6	-1.3
7	163.69	21.6	22.8	-1.2
8	161.19	27.2	25.5	1.7
8a	30.79	165.6	166.2	-0.6
9	123.63	66.2	66.0	0.2
10	56.39	138.2	138.6	-0.4
11	80.63	116.1	112.4	3.7
12	132.99	59.2	55.9	3.3
MAE				1.8

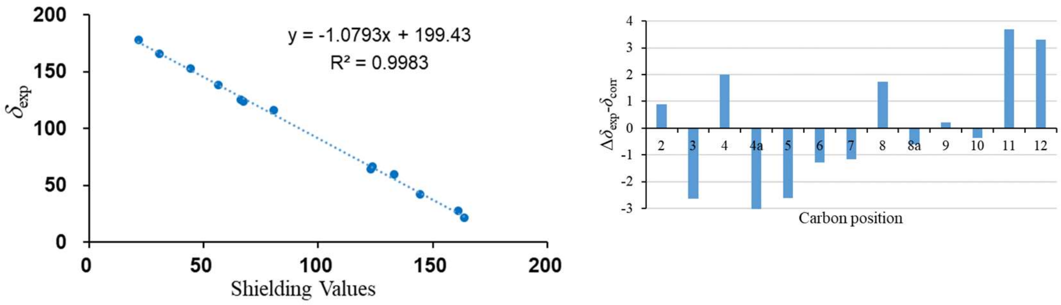

Fig. S97. Comparison of the calculated ^{13}C NMR data with the experimental ones of **12** in $CDCl_3$.

4.5 Computational details for 13

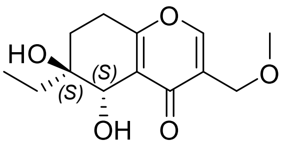

13a

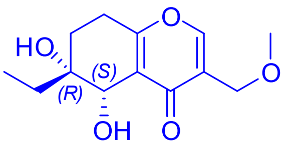

13b

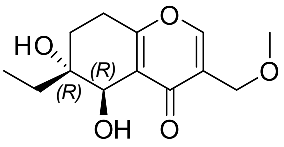

13c

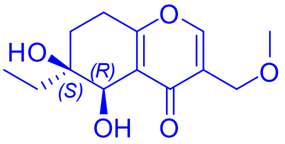

13d

Fig. S98. Possible structures of **13**.

A conformation search based on molecular mechanics with MMFF94s force fields was performed for **13a** and **13b**, giving seven and ten stable conformers with distributions higher than 1%.[1,2] All these conformers were further optimized by the density functional theory method at the B3LYP/6-31G(d,p) level in the Gaussian 16 program package,[3] leading to seven and nine conformers within a 3 kcal/mol energy threshold from the global minimum (Tables S10 and S11), respectively. Conformers of **13a** and **13b** were subjected to theoretical calculation of NMR using GIAO B3LYP/6-31G(d) level in chloroform based on optimized conformers. The calculated ^{13}C NMR data were compared with experimental data in Excel (Tables S12 and S13).

The predominant conformers of **13b** were subjected to theoretical calculation of ECD using TDDFT at B3LYP/6-31G(d,p) level with CPCM model in air based on B3LYP/6-31G(d,p) optimized conformers. The calculated ECD curve for **13b** was weighted using SpecDis.[4] The ECD curve of the enantiomers was generated by SpecDis by the function "enantiomeric ECD".

Table S10. Energy analysis for conformers of **13a** at B3LYP/6-31G(d,p) level in the gas phase

Species	Gibbs free energy	ΔE (kcal/mol)	P_E%
**13a-1**	-882.051202	0.00	63.2
**13a-2**	-882.048985	1.39	6.0
**13a-3**	-882.048917	1.43	5.6
**13a-4**	-882.049666	0.96	12.4
**13a-5**	-882.049164	1.28	7.3
**13a-6**	-882.047728	2.18	1.6
**13a-7**	-882.048562	1.66	3.9

Table S11. NMR calculation details of **13a**

No	Shielding value	Exp	Corr	Δ
2	43.88	152.7	151.1	1.6
3	66.45	125.1	127.2	-2.1
4	20.50	178.6	175.8	2.8
5	114.82	68.8	75.9	-7.1
6	117.45	72.4	73.1	-0.7
7	160.02	27.9	28.0	-0.1
8	162.72	24.8	25.2	-0.4
9	123.64	66.2	66.6	-0.4
10	162.56	29.3	25.3	4.0
11	180.81	6.8	6.0	0.8
12	132.86	59.2	56.8	2.4
4a	69.77	122.1	123.6	-1.5
8a	30.74	165.6	165.0	0.6
MAE				2.6

Table S12. Energy analysis for conformers of **13b** at B3LYP/6-31G(d,p) level in the gas phase

Species	Gibbs free energy	ΔE (kcal/mol)	P_E%
**13b-1**	-882.056425	0.00	58.4
**13b-2**	-882.055144	0.80	15.0
**13b-3**	-882.054904	0.95	11.7
**13b-4**	-882.054063	1.48	4.8
**13b-5**	-882.054184	1.41	5.4
**13b-6**	-882.050846	3.50	0.2
**13b-7**	-882.053203	2.02	1.9
**13b-8**	-882.052709	2.33	1.1
**13b-9**	-882.051149	3.31	0
**13b-10**	-882.052936	2.19	1.5

Table S13. NMR calculation details of **13b**

No	Shielding value	Exp	Corr	Δ
2	43.81	152.7	151.3	1.4
3	66.88	125.1	126.8	-1.7
4	19.85	178.6	176.8	1.8
5	118.57	68.8	71.9	-3.1
6	119.45	72.4	70.9	1.5
7	160.22	27.9	27.6	0.3
8	163.36	24.8	24.2	0.6
9	123.78	66.2	66.3	-0.1
10	157.13	29.3	30.9	-1.6
11	180.04	6.8	6.5	0.3
12	133.10	59.2	56.4	2.8
4a	70.40	122.1	123.1	-1.0
8a	29.30	165.6	166.8	-1.2
MAE				1.6

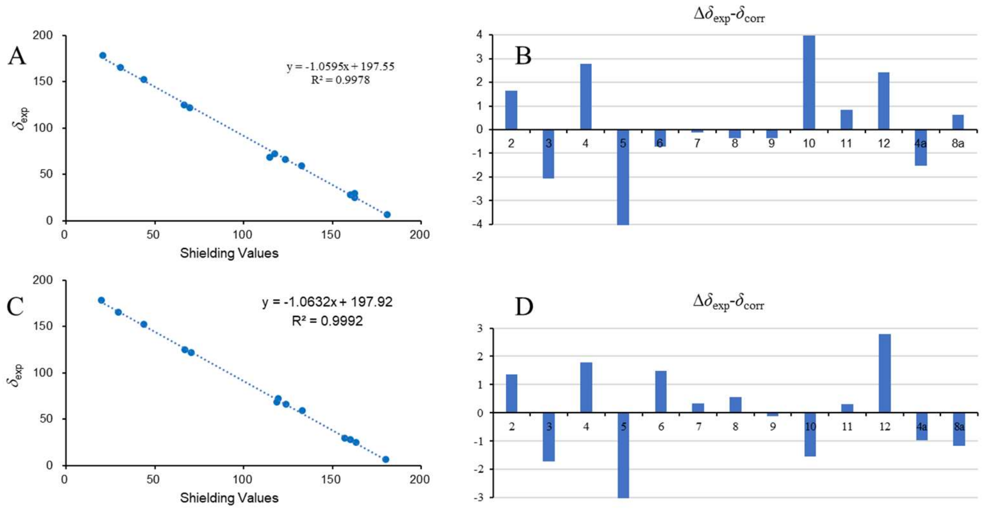

Fig. S99. Comparison of the calculated ^{13}C NMR data with the experimental ones of **13a** (A/B) and **13b** (C/D) in $CDCl_3$.

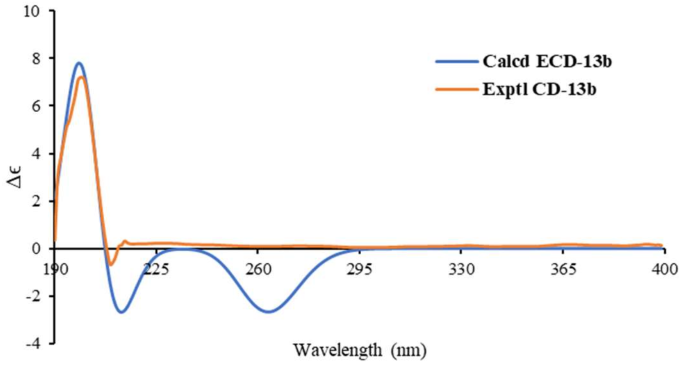

Fig. S100. Comparison of the calculated ECD spectrum for **13b** (σ = 0.20 eV, UV shift -10 nm) with the experimental spectrum of **13** in MeOH

4.6 Computational details for 14

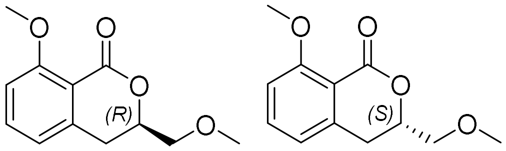

Fig. S101. Possible structures of **14**.

A conformation search based on molecular mechanics with MMFF94s force fields was performed for 3*R*-**14,** giving seven conformers with distributions higher than 1%.[1,2] All these conformers were further optimized by the density functional theory method at the B3LYP/6-311G+(d,p) level in the Gaussian 16,[3] leading to seven conformers within a 3 kcal/mol energy threshold from the global minimum (Table S14). Optimized conformers of 3*R*-**14** were subjected to theoretical calculation of optical rotations using the Ground State at B3LYP/6-31G(d,p) level with the CPCM model in methanol. The average value of 3*R*-**14** was weighted using Excel.

Table S14. Energy analysis and calculated rotations for conformers of (3*R*)-**14**

Species	Gibbs free energy	ΔE (kcal/mol)	$P_E\%$	Calculated [α]	Experimental [α]
(3*R*)-**14**-1	-766.381030	0.00	49.0	-27.33	
(3*R*)-**14**-2	-766.379092	1.22	6.3	0.96	
(3*R*)-**14**-3	-766.379951	0.68	15.6	271.90	
(3*R*)-**14**-4	-766.377746	2.06	1.5	-275.55	
(3*R*)-**14**-5	-766.379640	0.87	11.2	-79.93	
(3*R*)-**14**-6	-766.378921	1.32	5.2	63.67	
(3*R*)-**14**-7	-766.379633	0.88	11.1	-51.87	
Averaged [α]				13.54	96.2

References

(1) Goto, H.; Osawa, E.; *J. Am. Chem. Soc.* 1989, *111*, 8950–8951.

(2) Goto, H.; Osawa, E.; *J. Chem. Soc., Perkin Trans. 2*, 1993, 187–198.

(3) Frisch, M. J.; Trucks, G. W.; Schlegel, H. B.; Scuseria, G. E.; Robb, M. A.; Cheeseman, J. R.; Scalmani, G.; Barone, V.; Mennucci, B.; Petersson, G. A.; Nakatsuji, H.; Caricato, M.; Li, X.; Hratchian, H. P.; Izmaylov, A. F.; Bloino, J.; Zheng, G.; Sonnenberg, J. L.; Hada, M.; Ehara, M.; Toyota, K.; Fukuda, R.; Hasegawa, J.; Ishida, M.; Nakajima, T.; Honda, Y.; Kitao, O.; Nakai, H.; Vreven, T.; Montgomery, J. A.; Jr., Peralta, J. E.; Ogliaro, F.; Bearpark, M.; Heyd, J. J.; Brothers, E.; Kudin, K. N.; Staroverov, V. N.; Keith, T.; Kobayashi, R.; Normand, J.; Raghavachari, K.; Rendell, A.; Burant, J. C.; Iyengar, S. S.; Tomasi, J.; Cossi, M.; Rega, N.; Millam, J. M.; Klene, M.; Knox, J. E.; Cross, J. B.; Bakken, V.; Adamo, C.; Jaramillo, J.; Gomperts, R.; Stratmann, R. E.; Yazyev, O.; Austin, A. J.; Cammi, R.; Pomelli, C.; Ochterski, J. W.; Martin, R. L.; Morokuma, K.; Zakrzewski, V. G.; Voth, G. A.; Salvador, P.; Dannenberg, J. J.; Dapprich, S.; Daniels, A. D.; Farkas, O.; Foresman, J. B.; Ortiz, J. V.; Cioslowski, J.; and Fox, D. J.; Gaussian *09*, Revision B.01,Gaussian, Inc., Wallingford CT, 2010.

(4) Bruhn, T.; Schaumlöffel, A.; Hemberger, Y.; Bringmann, G. *Spec Dis*, version 1.71, University of Würzburg, Germany, 2012
